# Supplementary material for: Effects of psoriasis and psoralen exposure on the somatic mutation landscape of the skin
Source: Nat Genet. 2023 Oct 26;55(11):1892–900. doi: 10.1038/s41588-023-01545-1 (PMC10632143; doi:10.1038/s41588-023-01545-1)
Supplement: Supplementary file 1 — A more extensive description of the methods used in this study, extended results and discussion and R-markdown notebooks describing the key analyses supporting the manuscript. [file 41588_2023_1545_MOESM1_ESM.pdf]

nature genetics

Article

https://doi.org/10.1038/s41588-023-01545-1

Effects of psoriasis and psoralen exposure on the somatic mutation landscape of the skin

In the format provided by the authors and unedited

Supplementary Note 1 - Extended methods

1. Human tissue attainment and processing 2
    1.1 Tissue donation and fixation 2
    1.2 Laser-capture microdissection of epidermis 2

2. DNA sequencing 3
    2.1 Whole exome sequencing 3
    2.2 Whole genome sequencing 3

3. Somatic mutation calling 4
    3.1 Mutation calling in whole-exomes 4
        3.1.1 Substitution calling with CaVEMan 4
        3.1.2 Indel calling with cgpPindel 5
        3.1.3 Binomial filtering of somatic mutation calls 5
        3.1.4 Sensitivity of the substitution and indel calls from whole-exome data 5
        3.1.4 Structural variant calling in the exome data with ASCAT 6
    3.2 Mutation calling in whole-genomes 6

4. Identification of SNV clusters by hierarchical Dirichlet processing 7

5. Inference of phylogenetic trees 7

6. Mutational signatures 8
    6.1 Extraction of mutational signatures by hierarchical Dirichlet processing 8
    6.2 Characterization of the PUVA signature 9

7. Mutation burden estimation 11
    7.1 Adjusting the burden for coverage and VAF 11
    7.2 Linear mixed effect models to test for an effect of disease duration 12

8. Selection and driver analyses 13
    8.1 Exome-wide driver discovery 13
    8.2 Pathway-level selection 14
    8.3 Fraction of mutated cells 15

Methods references 15

1. Human tissue attainment and processing

1.1 Tissue donation and fixation

Round punch biopsies, 4 mm in diameter, from lesional and adjacent non-lesional skin were donated by psoriasis patients presenting to the Department of Dermatology, UKSH Kiel between 2017 and 2019. Biopsies were taken under sterile conditions under local anesthesia and the emerging holes were closed with seam stitching and bandaged. All donors gave informed consent for genetic research of the material and the study was approved by the research ethics committee of Christian-Albrechts University in Kiel (A100/12), the National Health Service (NHS) Research Ethics Committee (Yorkshire & The Humber - South Yorkshire Research Ethics Committee, REC ID 20/YH/0244, IRAS ID 286843) and by the Wellcome Trust Sanger Institute Human Materials and Data Management Committee (approval number 20/0085).

Biopsies were fixed in RNAlater (AM7021, ThermoFisher) upon collection following the manufacturer's instructions and frozen. One half of each biopsy was used in this study and one half retained for use in future projects.

1.2 Laser-capture microdissection of epidermis

Skin biopsies were processed using a Tissue Tek VIP 6 AI tissue processor (Sakura Finetek, Leiden, Netherlands) and embedded in paraffin. The blocks were sectioned into 10 µm thick sections using an Accu-Cut SRM 200 microtome (Sakura Finetek). Sections were fixed to 4 µm PEN membrane slides (11600288, Leica). Slides were stained with haematoxylin and eosin and imaged using a NanoZoomer 2.0-HT slide scanner (Hamamatsu Photonics, Hamamatsu, Japan). Samples of epidermis were dissected from this material using laser capture microdissection microscopy (LMD7000, Leica). Cells were lysed using Arcturus PicoPure DNA extraction kit (Applied Biosystems) according to the manufacturer's instructions.

The volume of the microbiopsies was determined by adding together the size estimates of the cuts from the LCM software and multiplying this by the thickness of the sections (10 µm). The same histological features from serial sections were often cut into the same well to increase the DNA-yield of the samples (a practice referred to as z-stacking). The surface area of the samples was determined by measuring the width of the samples along the basal membrane and multiplying this with the section thickness and the number of sections (z-stacks) separating the first and last sections dissected into the same well. When microdissecting, occasionally, individual microdissections do not drop to the bottom of wells but either fall outside a well or get stuck to the side of a well. Those dissections do not contribute any DNA to the

sample but are nevertheless part of the volume and surface area estimation. The size estimates should therefore be considered upper bound estimates.

To assess the sensitivity of the mutation calling, we cut 18 technical duplicate samples (see section 3.1.4). These were cut either from histological sections directly adjacent to the original samples (where the same histological features were clearly visible) or using a "braid sampling" strategy, where alternate z-stacks were cut into different wells of the sample collection plate.

2. DNA sequencing

2.1 Whole-exome sequencing

1207 microbiopsies (Supplementary Table 2) from 111 individuals were whole-exome sequenced on Illumina NovaSeq 6000® machines using 150bp paired-end reads and the Agilent SureSelect Human All Exon V5 bait set (S04380110). Paired-end reads were aligned to the human reference genome (build hg38) using BWA-MEM[1]. PCR duplicates were marked using biobambam[2] and duplicate statistics were calculated using Picard (v.1.131) (http://broadinstitute.github.io/picard/). Sample contamination estimates were calculated using VerifyBamID (v1.1.3)[3] and the on-target coverage was calculated using samtools (v.1.11) depth command, considering only reads with base quality and mapping quality greater than 30. The median on target coverage for all samples was 56X (range: 18-190) for the dataset as a whole but 58X and 46X for microbiopsies from lesional and non-lesional skin, respectively. The difference in coverage is due to the greater volume of the microbiopsies from lesional skin (see Extended Data Figure 1), which results in greater library complexity and translates to lower PCR duplicate rate and higher coverage. We cut columns of cells and the thicker epidermis of lesional skin results in a greater sample volume for the same surface area covered.

2.2 Whole-genome sequencing

Whole-genome sequencing was performed on 16 microbiopsies from 3 donors (patients 18,21 and 34) which showed the signature of PUVA exposure in the WES data (see main text). The sequencing was carried out in two pools of 8. Samples from patients 18 and 21 were multiplexed together in one run on an Illumina NovaSeq 6000® machine and samples from patient 34 in a second run. The median coverages were 23.3X, 25.5X and 22.9X for microbiopsies from patients 18, 21 and 34, respectively. The specific microbiopsies were selected for whole-genome sequencing on the basis of relatively high levels of clonality and limited relatedness on the phylogenetic trees constructed from the WES data. Higher clonality results in a greater sensitivity for calling mutations when sequencing to 20-30X coverage. The limited sharing of mutations allows more independent mutation events to be identified.

3. Somatic mutation calling

3.1 Mutation calling in whole-exomes

Substitution and indel calling in the whole-exomes was carried out in three steps: discovery, initial filtering of the call set and genotyping of all mutations called in an individual in all samples from that sample donor. Further filtering was applied after genotyping somatic mutations as described below.

3.1.1 Substitution calling with CaVEMan

Substitutions were called using CaVEMan (v.1.15.1) (Cancer Variants through Expectation Maximization) (https://cancerit.github.io/CaVEMan/)[4]. Mutations were called against an unmatched normal with the copy number options manually set to 10 and 2 for the mutant and wild-type copy numbers, respectively. The samples were compared against a normal panel consisting of 75 unrelated normal samples to remove common SNPs. Mutations were further filtered if the reads reporting the mutations had a median alignment score lower than 140 or if >50% of the reads were clipped.

All mutations passing these filters in any sample from a donor were next genotyped in all samples from that donor. Mutations could thus be called in a microbiopsy if they failed the criteria above assuming they had passed the filtering criteria in another microbiopsy from the same individual (for example, a mutation in a clone that dominates in one microbiopsy and extends into an adjacent microbiopsy but at lower levels could still be called in the latter sample). We used the bam2R() function of the deepSNV package (v. 1.40.0) in R[5] to generate pileups of all sites mutated in any sample using only mapped reads that had base quality and mapping quality greater than 30 and which were mapped in a proper pair, were not PCR duplicates, were the primary alignment and which passed platform quality check (sam flags 3847 see https://broadinstitute.github.io/picard/explain-flags.html). After these filters, we required a coverage of at least 4X at the site and at least three reads reporting the alternate allele in at least one sample from a donor to call a mutation.

Adjacent substitutions called in the same sample were merged into a double-base substitution call if the number of reads reporting the reference and alternative alleles were not significantly different (Fisher test).

3.1.2 Indel calling with cgpPindel

Indels were called using cgpPindel (v.3.5.0) (https://github.com/cancerit/cgpPindel)[6] using the same unmatched normal sample that was used to call substitutions. We generated pileups of the indel calls in the same way as described above for substitutions and required a coverage of at least 4X and at least three reads reporting the alternate allele in at least one sample from a donor to call a mutation as before.

3.1.3 Binomial filtering of somatic mutation calls

To filter rare germline variants not removed by the comparison with the normal panel we applied an exact binomial test of the number of reads reporting each mutation, as previously described[7]. Heterozygous germline variants are expected to be present at a VAF of 0.5 in every sample from a patient. For each mutation, we compared the number of reads reporting the reference and alternate alleles across all samples from that patient. We tested the hypothesis that the read counts for the variants were drawn from a binomial distribution with a probability of success of 0.5, or 0.95 for mutations on the sex chromosomes in men. We applied Benjamini-Hochberg correction for multiple testing and excluded mutations with $q>10^{-3}$. We also used binomial filtering to remove erroneous mutation calls. Recurrent sequencing artefacts will be randomly distributed across samples and can be modelled as being drawn from a binomial distribution. In contrast, true somatic mutations will have a high VAF in some samples whilst being completely absent from others. The latter are best represented by a beta-binomial with a high overdispersion. For every mutation call, we calculated the maximum likelihood overdispersion parameter (ρ) in a grid-based way (ranging the value of ρ from 10^{-6} to $10^{-0.05}$), like previously described[7]. Calls with $\rho<0.1$ were filtered as likely artifactual.

3.1.4 Sensitivity of the substitution and indel calls from whole-exome data

To estimate the sensitivity of the mutation calls we sequenced 18 technical duplicate samples and compared the mutations called in the original samples to those in the technical duplicates. The technical duplicates constitute the same histological features (rete ridges) as the original samples.
Assuming the same sensitivity in both samples, a maximum likelihood estimate for the sensitivity when mutations not present in either sample go unobserved is:

$$S = \frac{2\ \times n_2}{n_1\ +\ 2\times\ n_2}$$

Where n_2 is the number of mutations called in both samples and n_1 is the sum of mutations called in only one sample. Using this equation, we estimated a median sensitivity of 89% in the technical duplicates. This estimate should be considered to be a lower bound because:

1. The sensitivity depends on the coverage, which is uneven for the members of a pair.

2. Although the technical duplicates were cut from adjacent histological sections to the original samples, they may comprise slightly different combinations of keratinocyte clones compared with the original samples. Some mutations may therefore be truly unique to each sample. This does not apply to samples where alternate z-stacks were cut into different wells of the sample collection plate (referred to as "braid sampling" in Supplementary Figure 1D).

3.1.4 Structural variant calling in the exome data with ASCAT

The B-allele fractions and coverages were calculated at all common SNP sites (as defined by dbSNP build 150 for GRCh38p7) located within the exome panel using ConstructASCATFiles(https://github.com/MathijsSanders/ConstructASCATFiles). Normalised LogR and BAF values were generated by carrying out quality control and PCA analysis of the coverage using PREASCAT (https://github.com/MathijsSanders/PREASCAT). Nominated matched normal samples in Supplementary Table 1 were used as germline controls. Structural variants were called using Allele-Specific Copy Number Analysis of Tumors (ASCAT[8]; https://github.com/VanLoo-lab/ascat). A penalty score of 100 was used for segmentation. 101 microbiopsies were excluded from this analysis either due to the inability of ASCAT to find an optimal solution or if the goodness-of-fit for the optimal solution was <90%. We removed from the call list all segments smaller than 1 Mb in size, segments on chromosome X, segments within the extended MHC region (Mb 20-38 on chr6) and recurrent artifactual calls on Mb19 of chr14. We further removed segments that were also called in the matched normal of the sample or were called in microbiopsies from both lesional and non-lesional skin (which never showed a clonal relationship in the SNV data) or were called in all samples except 0, 1 or 2 from the same individual (these being likely germline in origin). Finally, we removed microbiopsies for which over 10 structural variants were called and which also had purity of less than 0.4, as we considered these likely problematic.

3.2 Mutation calling in whole-genomes

For the 16 microbiopsies that were whole-genome sequenced we used CaVEMan and cgpPindel to call and filter small variants in the same way as described above. However, in this case calling was performed against a matched normal sample chosen because it was phylogenetically unrelated to the index sample. This was possible because samples were whole-genome sequenced only after whole-exome sequencing and was preferable because the low number of samples per patient (and the lower aggregate coverage) reduces the accuracy of the binomial filtering of germline variants described above.

We used ascatNgs (internal version.4.5.0, https://github.com/cancerit/ascatNgs)[9] to estimate the copy number and purity of the samples. Those estimates were used by CaVEMan, rather than the copy number options being manually pre-set as described for the whole-exomes above.

4. Identification of SNV clusters by hierarchical Dirichlet processing

We implemented a nonparametric Bayesian hierarchical Dirichlet process (HDP) to cluster autosomal single base substitutions with similar variant allele fractions (VAFs). The full mathematical and implementation details of the model are described in a previous publication[10]. Briefly, clones of cells are present across different microbiopsies and this manifests as clusters of mutations that are found at similar VAFs. For every mutation, we have two vectors, one containing the number of reads reporting the alternate allele and another containing the total sequencing depth at each microbiopsy. We assume that each mutation can be assigned to exactly one cluster but the number of clusters is unknown. We aim to estimate the number of clusters present across all the microbiopsies dissected from a patient, the location of each cluster in the n-dimensional VAF hypercube and the allocation of mutations to each cluster.

We model the data using an N-dimensional Dirichlet process (NDP) clustering model, where the distribution of clone sizes and numbers follows a Dirichlet process. This has the advantage that there is no need to pre-specify the number of clusters present. Instead, mutations are moved around the clusters and in each sampling iteration, there is a defined probability that a mutation will initiate a new cluster which was not present in previous iterations. Clusters can also cease to exist if all member mutations are assigned to other clusters. Thus, the number of clusters varies throughout the sampling chain.

We ran the Gibbs sampler for 25,000 iterations, dropping the first 15,000 as burn-in. We used the ECR algorithm[11], implemented in the R package label.switching (v1.8), to resolve the label-switching problem associated with mixture models. To avoid overly complex solutions, we imposed an upper limit of 100 clusters per patient. We kept for downstream analysis only those clusters that were present at a minimum VAF of 0.05 in at least one microbiopsy and had a minimum of 10 unique mutations allocated to them.

5. Inference of phylogenetic trees

Each cluster of single-base-substitutions identified by the NDP algorithm represents a branch of the phylogenetic tree for that patient. We applied the statistical piegonhole principle to infer phylogenetic relationships between clusters. Given clusters A and B, if the combined mutant cell fraction (CF) of both is >100% (VAF > 0.5) within the same microdissections and B consistently shows a lower CF than A, then that is strong evidence that B is nested within A, that is mutation cluster B represents a sub-clone of clone A. If the combined mutant cell fraction is ≤100%, only weak evidence of nesting exists. If B is found at a higher VAF than A in some microdissections but at lower VAF in others, the clusters are interpreted as being independent clones without nesting. We treated each tip of the phylogenetic tree for each patient as a clone. The length of the branches from the root (germline) was used in the mutation burden calculations, as described below.

Some clusters are present at VAFs too low for the pigeonhole principle to be incontrovertible. In these cases, there is a risk that the cluster don't represent a single clone as assumed above, but a mixture of clones present at similar VAFs. We reconstructed the phylogenetic trees after pruning away branches where there is doubt about the validity of the pigeonhole principle. We retained nested clusters only if the sum of the cellular fraction estimates exceeds 1. Un-nested clusters (i.e branches of the phylogenetic tree consisting of a single cluster) were retained if the median VAF of the cluster is larger than 0.3.

6. Mutational signatures

6.1 Extraction of mutational signatures by hierarchical Dirichlet processing

To extract mutational signatures and estimate the exposure of each signature, we used a second hierarchical Dirichlet process[12], as implemented in the hdp (v.0.1.5) R package (https://github.com/nicolaroberts/hdp). HDP performs comparably to signature extraction methods that use non-negative matrix factorization but has the advantage of being able to model the relationships between samples and to simultaneously discover new signatures and quantifying the exposure to known signatures. Note that this is a separate implementation from the model used to cluster single base substitutions described above.

In this case, we sought to identify clusters of mutations corresponding to mutational signatures. The data was organised into a tree structure where the root contained all the mutations in the dataset. This node had as children one node that represented the most-recent common ancestor of all the patients and additionally frozen pseudo-count nodes for signatures that are to be used as priors in the model. The pseudo-nodes contained 10,000 pseudocounts each. We used signatures 1, 5, 2, 13, 7a, 7b, 7c, 7d, 17a, 17b, 18 and 38 as priors. During the Dirichlet process, mutations from the dataset may join the pseudocount clusters but the pseudocounts are frozen such that they are unable to leave the initial cluster. The patient ancestor node had as children one node for each of the patients and each patient node had as children one node for each branch of the phylogenetic tree (see the R-code accompanying this manuscript).

The hyperparameters for the α clustering parameter (α and β) were both set to 1. The model was initialised with 13 data clusters (number of priors plus one, parameter 'initcc'). After the initiation, an MCMC algorithm (Gibbs sampler) probabilistically moves each individual mutation to a cluster with a high proportion of mutations in the same category and/or a high proportion of mutations in that branch and/or parent node. The first 100,000 iterations of the Gibbs sampler were not collected (parameter 'burnin') but after that we sampled the posterior 200 times (parameter 'n') every 2000 Gibbs iterations (parameter 'space'). We sampled the concentration parameter three times (parameter 'cpiter') after each Gibbs sampling. We ran 20 chains using different random seeds and combined the results for signature extraction. Any clusters with a cosine similarity greater than 0.9 were merged while clusters with no

significant data categories or sample exposures were combined into a null signature, representing the fraction of the data that is unexplained by the model.

The model extracted nine signature components in addition to the Unassigned component (Extended Data Figures 2 and 3). Among these were components corresponding to COSMIC signatures SBS7b, SBS1/5, SBS2, SBS7c and SBS13, all of which were included as priors in the model. A novel component characterised by mutations at TpA sites accounted for just under 11% of the mutations in the dataset. This is the signature we attribute to psoralen exposure, which in the context of psoriasis is likely to occur during treatment with psoralens and high-dose UV-A (PUVA), see the main text. Finally, three additional components were extracted, Unknown components N1-3 in Supplementary Figure 2. These together accounted for 2.6% of the mutations in the dataset. They may represent individual variation in repair of UV-damage or they may be artefacts of the signature extraction model. We do not have sufficient confidence in these components to draw conclusions from them and have added them to the Unassigned component for subsequent analyses.

The model did not extract the UV-related SBS7a and SBS7d. The Unassigned component has a dominant T>C peak that likely corresponds to the prominent peak from SBS7d (Extended Data Figure 2). The SBS7b component may be a composite component consisting of a mixture of SBS7b and SBS7a which could not be separated by the model.

6.2 Characterisation of the psoralen signature

From the whole-exome data, we identified a number of samples which showed a large number of mutations at TpA sites, consistent with the known mutagenic effects of psoralens[13–15]. To enable further characterization of this signature, we selected 16 microbiopsies from patients showing clear evidence of psoralen exposure for whole-genome sequencing.

To visualise the trinucleotide and pentanucleotide spectrums associated with psoralen exposure, we used the R-package MutationalPatterns[16] (v. 3.4.0) together with BSgenome (v 1.60.0). To calculate the transcriptional strand bias, we used the gene definitions from the R-package TxDb.Hsapiens.UCSC.hg38.knownGene (v 3.13.0) and the strand_occurrences() and strand_bias_test() functions from MutationalPatterns.
To test for transcription coupled damage, we carried out a similar analysis to that originally used to describe transcription coupled damage in liver cancers[17]. We divided protein coding genes into quintiles by ascending expression in sun-exposed skin from the GTEx dataset[18] (v8). We extracted the transcriptional start site (TSS) and the strand of each gene from Gencode (v27) and defined ten 1kb bins upstream and downstream of the TSSs. We pooled T>[ACG] and A>[CGT] mutations at TpA or ApT sites from all whole-genome sequenced samples. If the gene is on the (-) strand, the transcribed strand is the reference and we counted the number of T>[ACG] mutations overlapping each 1kb bin. If the gene is on the (+) strand, the transcribed strand is the complement of the reference and we counted the number of A>[CGT] mutations. This was reversed for the untranscribed strand. We observed a drop in the mutation rate on the transcribed strand upstream of the TSSs, indicating transcription coupled repair. However, we also found an increased mutation burden on the untranscribed strand, indicative of transcription coupled

damage of this strand. To test the statistical significance of the increased mutation burden, we fit two linear models with and without a parameter indicating if each position was upstream or downstream of the TSSs and used a likelihood ratio test to test if the fit of the model was improved. Figure 3c shows the mutation rate in each 1kb bin relative to the -10kb bin, the intergenic bin furthest from the TSS.

To test the effect of gene expression levels on PUVA mutagenesis we again used expression data from sun-exposed skin from the GTEx dataset[18] (v8). We split protein coding genes into 10 equally sized bins by ascending levels of expression. We used the Bedtools[19] intersect function (v. 2.18) to count the number of mutations overlapping genes in each bin. Figure 3d shows the relative mutation rate in each bin compared to the lowest expression bin.

To assess the potential functional effects of the psoralen signature relative to other major signatures in the skin (Figure 3f), we used the context_potential_damage_analysis() and signature_potential_damage_analysis() functions of the MutationalPatterns package in R. Considering only the genes previously reported to be under selection in squamous cell carcinomas and/or normal skin, we counted the number of trinucleotide changes expected to give rise to the different types of mutations (Synonymous, Missense, Nonsense or Splice site). For each of the major signatures in the skin (Psoralen, SBS1, SBS5, SBS7a and SBS7b), we normalized the expected fraction of mutations falling in each annotation class by the fractions that would be expected given either a uniform mutation rate or SBS7b. We note that while this analysis gives a hint of how damaging a signature might be, it is only based on a trinucleotide mutational context and other important features such as gene expression, strandedness or the extended nucleotide context are not taken into account. The genes we used in this analysis are *AJUBA, ARID2, ASXL1, CASP8, CDKN2A, FAT1, KMT2D, NOTCH1, NOTCH2, NOTCH3, PPM1D, RB1, RBM10, TP53, TP63*.

The replication strand is dependent on the replication origins, which for the human genome are only partially known and likely vary to some extent between cell types. An analysis of replication strand bias requires replication timing data and must assume that the position of the replication origins are at least partially conserved between the cell type of interest and the cell type in which the timing of replication was established. We used two external sources of replication timing data. The results reported in Supplementary Figure 4 use data from a previous analysis of replication timing[17] that focused on "timing transition regions", which are highly conserved across cell types and species of eukaryotes[20,21]. As a second independent validation, we used replication timing data from the ENCODE project[22] that is provided with the MutationalPatterns package and obtained similar results.

To assess the effect of replication timing on psoralen-related mutagenesis, we used the same replication timing data as for the replication strand analysis described above. The genome was split into four bins in ascending order of replication timing. For the regions in each bin, we estimated the psoralen mutation rate by counting the number of T>[ACG]/A>[CGT] mutations overlapping the regions and dividing this by the number of TpA/ApT sites in each bin. The mutation overlap was determined using the Bedtools[19] intersect function (v. 2.18).

To see if psoralen exposure was associated with increased levels of clonal spread, we defined patients as psoralen exposed if at least one microbiopsy from the patient had over 100 mutations attributed to the psoralen signature. We then compared the VAFs between all microbiopsies dissected from exposed patients and microbiopsies dissected from non-exposed patients using a Mann Whitney U test. To also test for a greater expansion of clones across microbiopsies, we generated spatial relationship matrices for lesional and non-lesional biopsies from which more than one microbiopsy was sequenced. From the histological images, we manually measured the distance between the centres of the microdissections. This data is made available through the Mendeley repository referred to in the main text. For all pairs of microbiopsies dissected from the same skin biopsy, we plotted the fraction of mutations the two microbiopsies shared in common as a function of the distance between the pair (Figure 3f). We tested if microbiopsy-pairs from psoralen-exposed patients were enriched among the set of pairs that were both distant (separated by >500 micrometres) and which shared a high fraction of their mutations (>10%) using a Chi-square test. The cut-offs described in this paragraph were arbitrarily chosen but the results of both of these tests hold true across a range of threshold values.

7. Mutation burden estimation

7.1 Adjusting the burden for coverage and VAF

The number of mutations detected depends partially on both the sequencing coverage and clonal composition of the microbiopsy. In this study, we required a minimum coverage of 4X of a site and at least 3 reads supporting the alternative allele to call a mutation. Higher coverage enables the detection of more sub-clonal variants while greater polyclonal composition means the available reads are split between more clones. Any comparison of the mutation rate must take this technical variability into account.

To adjust the mutation burden of each cluster for coverage and VAF, we estimated the sensitivity for calling a true mutation belonging to the cluster in each microbiopsy, S_{bi}. The total sensitivity of the cluster, S_c, is one minus the sensitivity of the cluster in each microbiopsy, S_{bi}, or formally:

$$S_c = 1-(1-S_{b1})*(1-S_{b2})*...*(1-S_{bi})*...*(1-S_{bn})$$

The microbiopsies in which a cluster is present were sequenced to varying depths and the clusters are present at different cell fractions across different microbiopsies. To estimate the sensitivity for mutations in a given cluster in a microbiopsy, we fitted a truncated binomial distribution to the VAF distribution of the mutations assigned to the cluster in each microbiopsy (this is different from the estimated median VAF, as low VAF mutations may be missed at low coverage). We next simulated 100,000 mutation call attempts by drawing the coverage of each call from a Poisson distribution with the lambda parameter set as the median on-target coverage of that microbiopsy and multiplying that with the VAF estimate from the truncated binomial. The product represents the number of reads that report the alternative allele in

the simulation, and the estimated sensitivity is the fraction of simulations in which this value was greater than four.

7.2 Linear mixed effect models to test for an effect of disease duration

We used linear mixed effect models to compare the mutation burdens of lesional and non-lesional skin. We used as response variables the estimates of the mutation burdens of SBS7 and SBS1/5 after correcting for VAF and coverage as described above. The models include fixed effects for age and the anatomical location of the biopsy from which the clone is derived. Our observations are not independent. Cell clones from the same biopsy are likely to have correlated levels of UV-exposure and some correlation is also likely to exist between biopsies taken from the same patient. To model this, we include random effects for patient and biopsy, with the effect of biopsy being nested within that of the patient.

Most embryonic mutations will be filtered as germline and so at birth the exonic mutation count of the skin is close to zero. Rather than including a random intercept in our models, we constrain the intercepts to zero. The biological interpretation being that no (exonic) somatic mutations are present in the skin at birth.

Thus, the basic model, M_0, of mutation burden in clone i attributed to signature, S, is:

$$M_0: Burden_{S,i} = \beta_1 \times Age_i + \beta_2 \times Location_i + (\beta_3 + \beta_4) \times Patient_i Biopsy_i + \epsilon_i$$

Where β_1 is the fixed effect of age, β_2 is the fixed effect of anatomical location of the sample, $\beta_3 \sim N(0, \sigma_p)$ is the random effect of patient and $\beta_4 \sim N(0, \sigma_{pb})$ is the nested random effect for biopsy within a person. $\epsilon_i \sim N(0, \sigma_e)$ is a normally distributed error term.

We wish to know if psoriasis affects the mutation burden of keratinocytes. The ideal predictor variable would be some measure of 'inflammation exposure' which might comprise both the number of flare ups and the severity of each flare up. We lack this information for our patients, many of whom have had psoriasis for decades and been managed by different dermatologists during that time. Instead, we use disease duration as a proxy for disease exposure, setting the disease duration of non-lesional samples to zero before fitting the model. We note that in the event that some of the non-lesional samples are from areas of the skin which have been previously affected by the disease, our estimate of the disease effect would be smaller and this approach is therefore conservative.

To test for an effect of disease duration on the mutation burden of signature S, we define a second model, M_1, which includes a fixed effect for disease duration, β_5 .

$$M_1: Burden_{S,i} = \beta_1 \times Age_i + \beta_2 \times Location_i + \beta_5 \times DiseaseDuration + (\beta_3 + \beta_4) \times Patient_i Biopsy_i + \epsilon_i$$

We use a likelihood ratio test to see if adding disease duration significantly improves the fit of the model. The null hypothesis is $H_0 : \beta_5 = 0$ versus $H_1 : \beta_5 \neq 0$.
We calculate a likelihood ratio statistic, D, as

$$D = -2 \times ln(\frac{L(M_0)}{L(M_1)}) = 2 \times [LL(M_1) - LL(M_0)]$$

Where $L(M_i)$ and $LL(M_i)$ are the likelihoods and the log-likelihoods of the two models, respectively. Under the null hypothesis, D follows a chi-squared distribution with one degree of freedom, giving a P-value for the disease duration effect.

8. Selection and driver analyses

We used the dNdScv software (v. 0.0.1.0)[23] (https://github.com/im3sanger/dndscv) to identify genes enriched in non-synonymous mutations, indicative of positive selection. The ratio of non-synonymous (dN) over synonymous (dS) mutation rate (dN/dS) is a widely used measure of evolutionary pressures. dNdScv is a maximum-likelihood implementation of this method, specifically developed for somatic mutation data. dN/dS ratios for each gene (or groups of genes together) are calculated using a substitution model that takes into account the sequence composition of the genes, the trinucleotide context of the observed mutations, and transcriptional strand biases. Mutation densities vary substantially depending on chromatin state, replication timing and expression levels of genes. This is modelled by dNdScv using epigenetic covariates calculated from the ENCODE[24] project and the Cancer Genome Atlas (TCGA). We refer to the original dNdScv publication for an extensive description of the method.

Double-base substitutions (DBS) represent a small problem to dndscv, because the same mutation event can affect more than one codon and give rise to two annotation types of mutations (for example a missense and a synonymous change). DBSs are by default combined with indels in a negative-binomial model which estimates the mutation frequency across the genome without taking the sequence context into account. This typically has only a small effect on the model, as the number of DBSs is small. In the current dataset however, UV-light exposure has resulted in a large number of DBS mutations. We therefore fitted separate negative binomials for indels and for DBSs and combined the P-values with those obtained from analysing single-base substitutions using Fisher's method (see Supplementary Code 4, an R-markdown file that accompanies this manuscript).

8.1 Exome-wide driver discovery

We first calculated dN/dS ratios across all coding genes using mutations identified in either lesional or non-lesional skin. Mutations shared between microbiopsies dissected from the same patient were collapsed into single mutation events to avoid counting them multiple times. This assumes that mutations are much more likely to be observed across multiple samples from an individual because the samples

share a cellular origin than the same (hotspot) site being independently mutated multiple times. For the purposes of identifying selection, the former explanation is the more conservative.

As described in the main text, this revealed positive selection of mutations in nine genes. While not all genes have been identified in studies of normal skin, they all are likely all under selection in normal squamous epithelial tissues and not specific to psoriasis. We confirmed that the results are robust against the use of covariates, inclusion of hypermutator samples (which add many passengers and comparatively few drivers) and the inclusion of microbiopsies from non-lesional skin (Supplementary Code 4).

We performed restricted hypothesis testing of 27 genes in which mutations have been reported to be under selection in either the normal skin or normal oesophagus[25–28] but identified no additional genes under selection. We also used the sitednds() function of the dndscv package to look for recurrently mutated sites and mutation hotspots but this did not reveal selection in any additional genes.

8.2 Pathway-level selection

To search for an enrichment of mutations affecting particular gene sets or pathways relevant to psoriasis we defined eleven gene sets *a priori* as follows.

A list of genes previously found to be recurrently mutated either in normal skin or in squamous cell carcinomas was compiled by taking the union of all genes found to be under positive selection in Martincorena et al[25] or Fowler et al[28]. To this list, we added *CDKN2A*, *MLL2*, and *CASP8*, which are recurrently mutated in cutaneous squamous cell carcinomas but have not been reported to be under positive selection in the normal skin[25].

A second list of genes reported to be recurrently mutated in basal cell carcinomas was obtained from Bonilla et al[29]. From this list, we excluded *TP53*, *NOTCH1* and *NOTCH2* so mutations in these genes would not drive enrichment of the pathway, which otherwise contains many genes involved in Sonic Hedgehog signalling.

The third list consisted of genes implicated in psoriasis pathogenesis through GWAS[30]. Assigning genes to GWAS signals is an on-going problem within the field of human genetics. In this work, we used the Open Targets Genetics portal[31] to assign a gene to each GWAS locus. For loci where any gene achieved an L2G score of >0.5, we used that gene but otherwise used the gene closest to the variant with the lowest P-value in the GWAS analysis.

The fourth list contained genes identified as under positive selection in any of three recent papers describing somatic evolution in IBD affected colonic mucosa[7,32,33]. The three papers identify distinct patterns of selection in the IBD-affected colon compared with the normal colon, characterised by selection of immune-related genes, including *PIGR*, *ZC3H12A*, *NFKBIZ* and other genes in the IL-17 and toll-like receptor pathways. We included this gene set to test the hypothesis that similar selection forces might be operating across different types of epithelia under conditions of chronic inflammation. *PIGR* was excluded from the list, as this gene is not expressed in the skin.

Lists five through eleven are TNF signalling, IFNg signalling, IL12/23 signalling, IL36/MyD88 signalling, TLR signalling, IL-17 signalling and class I MHC mediated antigen processing and presentation. These pathways

have all been strongly implicated in psoriasis pathogenesis[34]. We used the Reactome database[35] to decide which genes to include in the definition of each pathway.

As described in the main text, the mutational signatures of UV-light and psoralen exposure dominate the dataset. Both have an effect of sequence context which extends beyond the trinucleotide model used by dNdScv. Using the trinucleotide model has previously been shown to result in arbitrarily low estimates of the number of driver mutations in melanomas[23]. To account for this, we implemented a pentanucleotide model which takes into account two base pairs on either side of the mutated base. For each of the gene sets described above, and using this model, we extracted the global dN/dS ratios for missense and nonsense variants separately. We corrected for 22 statistical tests (11 gene sets, missense and nonsense) using Benjamini-Hochberg correction. We believe the effects of the pentanucleotide model are especially important when aggregating mutation counts across multiple genes and that implementing this on a per-gene level would be unlikely to affect our results.

8.3 Fraction of mutated cells

We compared the fraction of cells that carry mutations in any of the nine genes that showed a significant enrichment of mutations between lesional and non-lesional skin. The fraction of cells carrying a mutation can be estimated from the fraction of reads that report the mutation in each sample (the variant allele fraction, VAF). We calculated the fraction of mutated cells separately in lesional and non-lesional biopsies from each individual by multiplying twice the VAF of each mutation in each microbiopsy by the volume of the microbiopsy and dividing that by the total volume of microbiopsies dissected from that skin biopsy. For clones that carry more than one mutation in the same gene, we only counted the mutation with higher VAF. When the second mutation was present in a sub-clone, we used only the mutation present in the parent clone.

Methods references

1. Li, H. Aligning sequence reads, clone sequences and assembly contigs with BWA-MEM. *arXiv [q-bio.GN]* (2013).
2. German Tischler, S. L. biobambam: tools for read pair collation based algorithms on BAM files. *Source Code Biol. Med.* **9**, 13 (2014).
3. Jun, G. *et al.* Detecting and estimating contamination of human DNA samples in sequencing and array-based genotype data. *Am. J. Hum. Genet.* **91**, 839–848 (2012).
4. Jones, D. *et al.* cgpCaVEManWrapper: Simple Execution of CaVEMan in Order to Detect Somatic Single Nucleotide Variants in NGS Data. *Curr. Protoc. Bioinformatics* **56**, 15.10.1–15.10.18 (2016).
5. Gerstung, M., Papaemmanuil, E. & Campbell, P. J. Subclonal variant calling with multiple samples and prior knowledge. *Bioinformatics* **30**, 1198–1204 (2014).
6. Raine, K. M. *et al.* cgpPindel: Identifying Somatically Acquired Insertion and Deletion Events from Paired End Sequencing. *Curr. Protoc. Bioinformatics* **52**, 15.7.1–15.7.12 (2015).

7. Olafsson, S. *et al.* Somatic Evolution in Non-neoplastic IBD-Affected Colon. *Cell* **182**, 672–684.e11 (2020).
8. Van Loo, P. *et al.* Allele-specific copy number analysis of tumors. *Proc. Natl. Acad. Sci. U. S. A.* **107**, 16910–16915 (2010).
9. Raine, K. M. *et al.* ascatNgs: Identifying Somatically Acquired Copy-Number Alterations from Whole-Genome Sequencing Data. *Curr. Protoc. Bioinformatics* **56**, 15.9.1–15.9.17 (2016).
10. Brunner, S. F. *et al.* Somatic mutations and clonal dynamics in healthy and cirrhotic human liver. *Nature* **574**, 538–542 (2019).
11. Papastamoulis, P. label.switching: An R Package for Dealing with the Label Switching Problem in MCMC Outputs. *J. Stat. Softw.* **69**, 1–24 (2016).
12. Teh, Y. W., Jordan, M. I., Beal, M. J. & Blei, D. M. Hierarchical Dirichlet Processes. *J. Am. Stat. Assoc.* **101**, 1566–1581 (2006).
13. Zhen, W. P., Buchardt, O., Nielsen, H. & Nielsen, P. E. Site specificity of psoralen-DNA interstrand cross-linking determined by nuclease Bal31 digestion. *Biochemistry* **25**, 6598–6603 (1986).
14. Sage, E., Drobetsky, E. A. & Moustacchi, E. 8-Methoxypsoralen induced mutations are highly targeted at crosslinkable sites of photoaddition on the non-transcribed strand of a mammalian chromosomal gene. *EMBO J.* **12**, 397–402 (1993).
15. Esposito, F., Brankamp, R. G. & Sinden, R. R. DNA sequence specificity of 4,5’,8-trimethylpsoralen cross-linking. Effect of neighboring bases on cross-linking the 5'-TA dinucleotide. *J. Biol. Chem.* **263**, 11466–11472 (1988).
16. Blokzijl, F., Janssen, R., van Boxtel, R. & Cuppen, E. MutationalPatterns: comprehensive genome-wide analysis of mutational processes. *Genome Med.* **10**, 33 (2018).
17. Haradhvala, N. J. *et al.* Mutational Strand Asymmetries in Cancer Genomes Reveal Mechanisms of DNA Damage and Repair. *Cell* **164**, 538–549 (2016).
18. Urbut, S. M., Wang, G., Carbonetto, P. & Stephens, M. Flexible statistical methods for estimating and testing effects in genomic studies with multiple conditions. *Nat. Genet.* **51**, 187–195 (2019).
19. Quinlan, A. R. & Hall, I. M. BEDTools: a flexible suite of utilities for comparing genomic features. *Bioinformatics* **26**, 841–842 (2010).
20. Ryba, T. *et al.* Evolutionarily conserved replication timing profiles predict long-range chromatin interactions and distinguish closely related cell types. *Genome Res.* **20**, 761–770 (2010).
21. Rhind, N. & Gilbert, D. M. DNA replication timing. *Cold Spring Harb. Perspect. Biol.* **5**, a010132 (2013).
22. Dunham, I. *et al.* An integrated encyclopedia of DNA elements in the human genome. *Nature* **489**, 57–74 (2012).
23. Martincorena, I. *et al.* Universal Patterns of Selection in Cancer and Somatic Tissues. *Cell* **171**, 1029–1041.e21 (2017).
24. Roadmap Epigenomics Consortium *et al.* Integrative analysis of 111 reference human epigenomes. *Nature* **518**, 317–330 (2015).
25. Martincorena, I. *et al.* Tumor evolution. High burden and pervasive positive selection of somatic mutations in normal human skin. *Science* **348**, 880–886 (2015).
26. Martincorena, I. *et al.* Somatic mutant clones colonize the human esophagus with age. *Science* **362**, 911–917 (2018).

27.Yokoyama, A. *et al.* Age-related remodelling of oesophageal epithelia by mutated cancer drivers. *Nature* **565**, 312–317 (2019).
28.Fowler, J. C. *et al.* Selection of Oncogenic Mutant Clones in Normal Human Skin Varies with Body Site. *Cancer Discov.* **11**, 340–361 (2021).
29.Bonilla, X. *et al.* Genomic analysis identifies new drivers and progression pathways in skin basal cell carcinoma. *Nat. Genet.* **48**, 398–406 (2016).
30.Tsoi, L. C. *et al.* Large scale meta-analysis characterizes genetic architecture for common psoriasis associated variants. *Nat. Commun.* **8**, 15382 (2017).
31.Ghoussaini, M. *et al.* Open Targets Genetics: systematic identification of trait-associated genes using large-scale genetics and functional genomics. *Nucleic Acids Res.* **49**, D1311–D1320 (2021).
32.Nanki, K. *et al.* Somatic inflammatory gene mutations in human ulcerative colitis epithelium. *Nature* **577**, 254–259 (2020).
33.Kakiuchi, N. *et al.* Frequent mutations that converge on the NFKBIZ pathway in ulcerative colitis. *Nature* **577**, 260–265 (2020).
34.Lowes, M. A., Suárez-Fariñas, M. & Krueger, J. G. Immunology of psoriasis. *Annu. Rev. Immunol.* **32**, 227–255 (2014).
35.Fabregat, A. *et al.* The Reactome Pathway Knowledgebase. *Nucleic Acids Res.* **46**, D649–D655 (2018).

Supplementary Note 2 – Supplementary results and discussion

Mutation burden estimates

In the main text, we describe our strategy for using linear mixed effect models to jointly estimate the effects of age and disease duration on the total mutation burden of keratinocyte clones (excluding psoralen mutations) and on the burden of SBS1/5 specifically. We estimate mutation burdens for clones of keratinocytes after clustering mutations based on variant allele fractions (VAFs) and then using the pigeonhole principle to build phylogenetic trees. The mutation burden of each clone is the combined length of the branches for each tip of the tree (after correcting for coverage and VAF, see methods).

However, some of the mutation clusters consisted of groups of mutations with VAFs too low for the pigeonhole principle to be incontrovertible. The calculations above assume that in such cases, the mutations all derive from a single sub-clone. However, there is a risk that the mutation burden represents not the burden of a single clone but the sum of the mutation burden for a collection of clones with similar cell fractions across all microbiopsies in which they are observed. This would lead to an over-estimation of the mutation rate for terminal branches of the phylogenetic trees. We performed pruning of the phylogenetic trees, retaining only branches representing nested clusters if the sum of the VAFs was greater than 1. For branches that represent single clusters (with no nesting), we pruned branches with VAF<0.3. Repeating our linear mixed effects modelling, we estimate from the pruned trees that the total mutation rate excluding psoralens is 9.6 mutations per exome per year (9.6 (6.5-12.7, 95% CI), P=2.0E-8), the UV-specific mutation rate is 9 mutations per exome per year (9.0 (5.8-12.1, 95% CI), P=1.5E-7), the age effect of SBS 1/5 is estimated to be 0.69 mutations per exome per year (0.52 (0.39-0.66, 95% CI), P=2.3E-11) while the disease duration effect is estimated to be 0.07 mutations per exome per year (0.07 (-0.03-0.18, 95% CI), P=0.17). As expected, all effects become smaller when terminal branches of the trees are pruned, but the most important difference is that the effects of disease duration on the burden of SBS1/5 is not significantly different from zero in this analysis. We note that pruning of the phylogenetic trees can only ever shorten the tips of the phylogenetic trees and likely results in an underestimation of the mutation rate. An accumulation of 9.6 mutations per exome per year in the skin should therefore be considered a conservative lower bound estimate.

Psoralen signature

In the main text, we report a mutational signature which we attribute to psoralen exposure. We show that the mutational signature has a strong transcriptional strand bias which results both from transcription coupled repair and transcription coupled damage. Transcription coupled damage has been most comprehensively described for T>C mutations in COSMIC signature SBS16, which is found

almost exclusively in the liver and may result from alcohol consumption[1]. SBS16 is thought to result from the chemical modification of adenine residues which likely occurs more frequently when the DNA is in unwound form during transcription. Our data suggest that psoralen molecules similarly preferentially react with thymine in unwound DNA. Psoralens are used in experimental systems to study mutagenesis and the repair of interstrand cross-link lesions. It has been demonstrated that the repair of psoralen-related interstrand cross links does not depend on the Fanconi anaemia pathway but rather depends on the DNA glycosylase NEIL3, which facilitates the removal of the lesion without the formation of a double-strand break[2,3]. In agreement with this, we find no effect of psoralens on the burden of indels or structural variants, as might be expected if the cross-links required double strand breaks to resolve.

Structural variants lead to LOH of driver mutations

We used the ASCAT software[4,5] to call large (>1Mb) deletions, duplications and copy number neutral loss-of-heterozygosity (LOH) events in 1104 microbiopsies where ASCAT goodnes-of-fit exceeded 90%. After filtering variants (Methods), 95 duplications, 294 deletions and 46 LOH events were called, with most events affecting only small segments of chromosomes (see figure below). Events affecting whole chromosomes were only detected in two of the 1104 microbiopsies. A single gain of chromosome 12 was observed in one microbiopsy and a loss of chromosome 19 in another. We observed nine and three LOH events affecting *NOTCH1* and *FAT1*, respectively. Of these, eight and two events made putative driver mutations in these genes homozygous (see below). LOH events that render mutations in *NOTCH1* and *FAT1* homozygous have been previously reported both in the normal skin and in the normal esophagous[6-9].

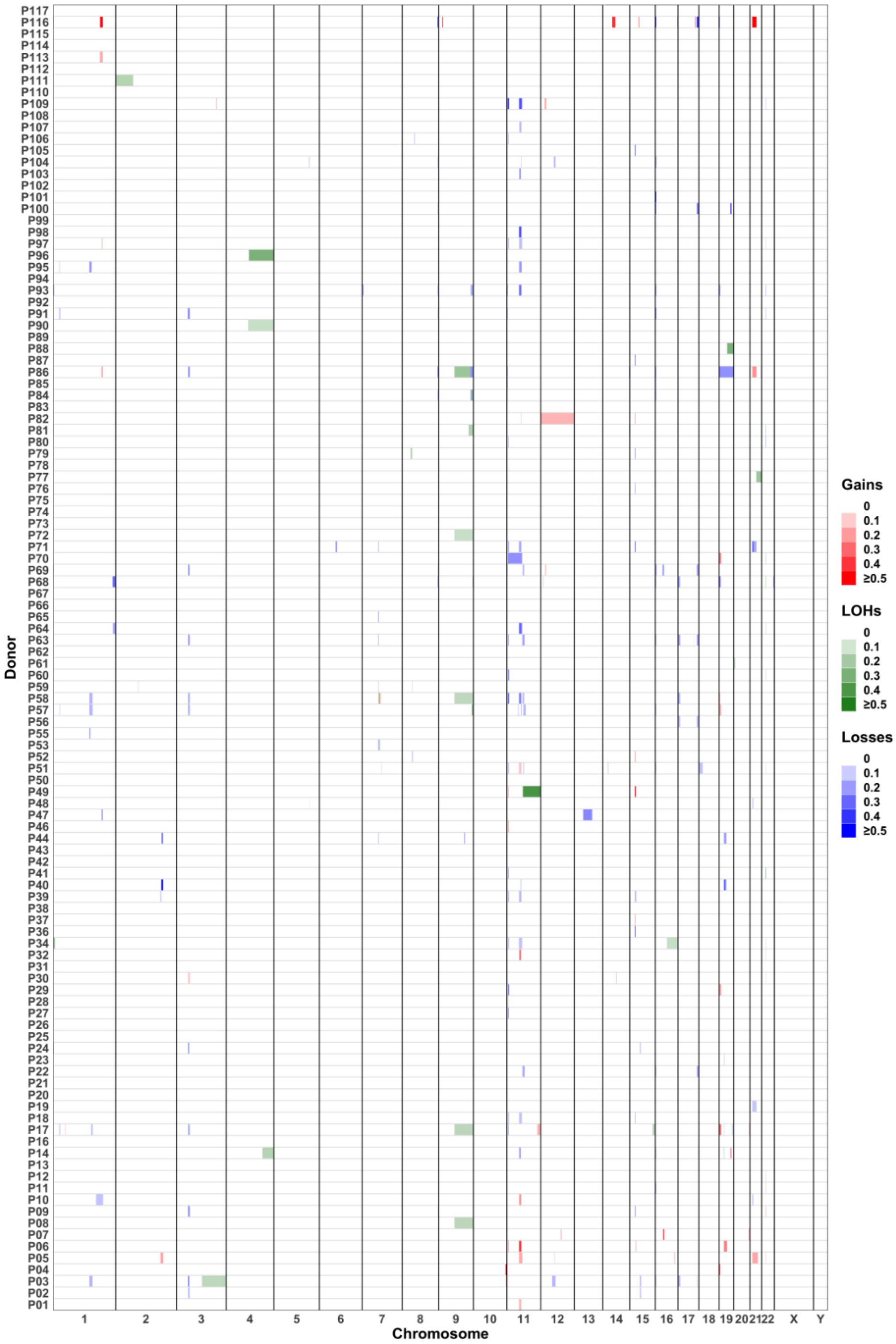

Supplementary Note Figure 1: Structural variants identified in microbiopsies from each patient. The intensity of the colours increases with the fraction of microbiopsies from each patient in which the variant is called.

Selection

As described in the main text, we found evidence of positive selection of mutations in nine genes. Of these, *NOTCH1*, *FAT1*, *PPM1D, TP53* and *NOTCH2* have been previously shown to be under positive selection in normal skin in studies that have used deep sequencing of targeted gene panels[6,7], while *GXYLT1*, *CHEK2*, *ZFP36L2* and *EEF1A1* have not been reported in these studies. Although these latter genes have not been reported before in studies of the skin, we hypothesise that these mutations do

not enhance fitness specifically in the context of psoriasis but may do so more generally across epithelial tissues.

Two of the genes, *GXYLT1* and *ZFP36L2* plausibly assert their effects through perturbation of the Notch signalling pathway, the most commonly mutated pathway in normal skin[6]. *GXYLT1* encodes a xylosyltransferase that adds xylose to O-glucose-modified residues on epidermal growth factor-like repeats, which are found on the extracellular domains of all Notch proteins and are where missense mutations cluster in *NOTCH1* (Extended Data Fig. 5). We are not aware that *GXYLT1* has been found to be recurrently mutated in keratinocyte cancers and it may be positively selected in normal skin without promoting cancer formation. *ZFP36L2* is an RNA binding protein of the tristetraprolin family of post-transcriptional regulators. It targets the 3' untranslated regions of multiple mRNAs, including those of *NOTCH1*[10,11]. Consistent with a general role in squamous epithelia, mutations in *ZFP36L2* have been reported to be under positive selection in normal oesophagus[9], a squamous epithelial tissue which has a similar selection landscape to the skin. *CHEK2* encodes checkpoint kinase 2, a cell cycle checkpoint regulator and a well-established tumour suppressor. Like *ZFP36L2*, it has been reported to be recurrently mutated in normal oesophagus[9] and selection of mutations in *CHEK2* seems unlikely to be a specific feature of psoriasis. Finally, *EEF1A1* encodes the alpha subunit of the elongation factor-1 complex. Unlike *GXYLT1*, *ZFP36L2* and *CHEK2*, it was included in an extended panel of genes tested for evidence of selection in a subset of normal skin samples in a previous study[6], but did not reach significance. *EEF1A1* did reach significance in our previous analysis of normal urothelium from bladder cancer patients[12], suggesting it too may be generally selected for in normal epithelial tissues and may not be specific to psoriasis.

References

1. Letouzé, E. *et al.* Mutational signatures reveal the dynamic interplay of risk factors and cellular processes during liver tumorigenesis. *Nat. Commun.* **8**, 1315 (2017).

2. Semlow, D. R., Zhang, J., Budzowska, M., Drohat, A. C. & Walter, J. C. Replication-Dependent Unhooking of DNA Interstrand Cross-Links by the NEIL3 Glycosylase. *Cell* **167**, 498–511.e14 (2016).

3. Li, N. *et al.* Cooperation of the NEIL3 and Fanconi anemia/BRCA pathways in interstrand crosslink repair. *Nucleic Acids Res.* **48**, 3014–3028 (2020).

4. Van Loo, P. *et al.* Allele-specific copy number analysis of tumors. *Proc. Natl. Acad. Sci. U. S. A.* **107**, 16910–16915 (2010).

5. Raine, K. M. *et al.* ascatNgs: Identifying Somatically Acquired Copy-Number Alterations from Whole-Genome Sequencing Data. *Curr. Protoc. Bioinformatics* **56**, 15.9.1–15.9.17 (2016).

6. Martincorena, I. *et al.* Tumor evolution. High burden and pervasive positive selection of somatic mutations in normal human skin. *Science* **348**, 880–886 (2015).

7. Fowler, J. C. *et al.* Selection of Oncogenic Mutant Clones in Normal Human Skin Varies with Body Site. *Cancer Discov.* **11**, 340–361 (2021).

8. Martincorena, I. *et al.* Somatic mutant clones colonize the human esophagus with age. *Science* **362**, 911–917 (2018).

9. Yokoyama, A. *et al.* Age-related remodelling of oesophageal epithelia by mutated cancer drivers. *Nature* **565**, 312–317 (2019).

10. Hodson, D. J. *et al.* Deletion of the RNA-binding proteins ZFP36L1 and ZFP36L2 leads to perturbed thymic development and T lymphoblastic leukemia. *Nat. Immunol.* **11**, 717–724 (2010).

11. Vogel, K. U., Bell, L. S., Galloway, A., Ahlfors, H. & Turner, M. The RNA-Binding Proteins Zfp36l1 and Zfp36l2 Enforce the Thymic β-Selection Checkpoint by Limiting DNA Damage Response Signaling and Cell Cycle Progression. *J. Immunol.* **197**, 2673–2685 (2016).

12. Lawson, A. R. J. *et al.* Extensive heterogeneity in somatic mutation and selection in the human bladder. *Science* **370**, 75–82 (2020).

Supplementary Note 3:

An R-markdown notebook describing the analyses done for looking for effects of treatments on the somatic mutation landscape.

CodeS1: Treatments

Sigurgeir Ólafsson

9/19/2022

Introduction

This document describes the treatment metadata and how we looked for treatment effects on the somatic mutation landscape of the skin in psoriasis patients.

```
.libPaths("/lustre/scratch126/humgen/projects/psoriasis/R_packages_farm5_R4.1.0_install/")
library(ggplot2)
library(reshape2)
library(cowplot)
library(ggsignif)
library(ggExtra)
library(MutationalPatterns)
library(GenomicRanges)
library(BSgenome)
ref_genome <- "BSgenome.Hsapiens.UCSC.hg38"
library(ref_genome, character.only = TRUE)
library(dplyr)
library(nlme)
library(scales)

## DEFINE PLOTTING VARIABLES
##############################
BASESIZE=18

working_dir="/nfs/users/nfs_s/so11/phd/psoriasis/bsub_jupyter_lab/psoriasis/manuscript_data_and_figures/"

microd_meta <- read.table(paste(working_dir, "Microdissection_metaData.txt", sep=""), h=T)
patient_meta <- read.table(paste(working_dir, "Patient_metaData.txt", sep=""), h=T)
treatment_data <- read.table(paste(working_dir, "treatment_data_combined.txt", sep=""), h=T)

patient_meta$Shows_Psoralen_signature <- ifelse(patient_meta$anyCloneGT50PUVA=="Yes" | patient_meta$Patient.ID %i
n% c("patient89","patient56","patient105"), T, F)
patient_meta <- merge(patient_meta, treatment_data, by.x="Patient.ID", by.y="PatientID", all.x=T)
patient_meta$Sex[patient_meta$Sex=="female"] <- "Female"
patient_meta$Sex[patient_meta$Sex=="male"] <- "Male"
```

Visualize the treatment data

We see that we have no dosage information for methotrexate or topical steroids. For the phototreatments, we have information about the cumulative doses

```
puva <- patient_meta[,c("Patient.ID", "AmountPuva", "EverPuva")]
puva$Treatment <- "PUVA"
colnames(puva)[2] <- "NrSessions"
colnames(puva)[3] <- "Treated"
uvb <- patient_meta[,c("Patient.ID", "AmountUVB", "EverUVB")]
uvb$Treatment <- "UVB"
colnames(uvb)[2] <- "NrSessions"
colnames(uvb)[3] <- "Treated"

methotrexate <- patient_meta[, c("Patient.ID","EverMethotrexate")]
colnames(methotrexate)[2] <- "Treated"
methotrexate$Treatment <- "Methotrexate"

steroids <- patient_meta[, c("Patient.ID","EverSteroids")]
colnames(steroids)[2] <- "Treated"
steroids$Treatment <- "Topical Steroids"

photo <- rbind(puva, uvb)
photo$NrSessions[photo$NrSessions=="unknown"] <- "Unknown (>0)"
photo$NrSessions <- factor(photo$NrSessions, levels=c("None","Unknown (>0)", "<=50", "51-200", ">200"))

treatments <- rbind(puva[,c(1,3,4)], uvb[,c(1,3,4)],methotrexate, steroids)

treatments$Treated <- ifelse(treatments$Treated, "Treatment history", "No history")
treatments$Treated <- factor(treatments$Treated, levels=c("Treatment history", "No history"))
p1 <- ggplot(treatments, aes(x=Treatment,fill=Treated)) + geom_bar() + theme_classic(base_size = BASESIZE) +
  labs(x="", y="# Patients", fill="") +
  theme(axis.text.x = element_text(angle=90)) + scale_y_continuous(expand = c(0,0)) +
  theme(legend.position = "bottom") + scale_fill_brewer(palette = "Set1")

p2 <- ggplot(photo, aes(x=NrSessions)) + geom_bar(fill="gray") + theme_classic(base_size = BASESIZE) +
  facet_wrap(~Treatment) + labs(x="# Treatment sessions", y="# Patients") +
  theme(axis.text.x = element_text(angle=90)) + scale_y_continuous(expand = c(0,0), limits = c(0,111)) +
  scale_fill_brewer(palette = "Set2")

plot_grid(p1,p2, align="v")
```

```
## Warning: Graphs cannot be vertically aligned unless the axis parameter is set.
## Placing graphs unaligned.
```

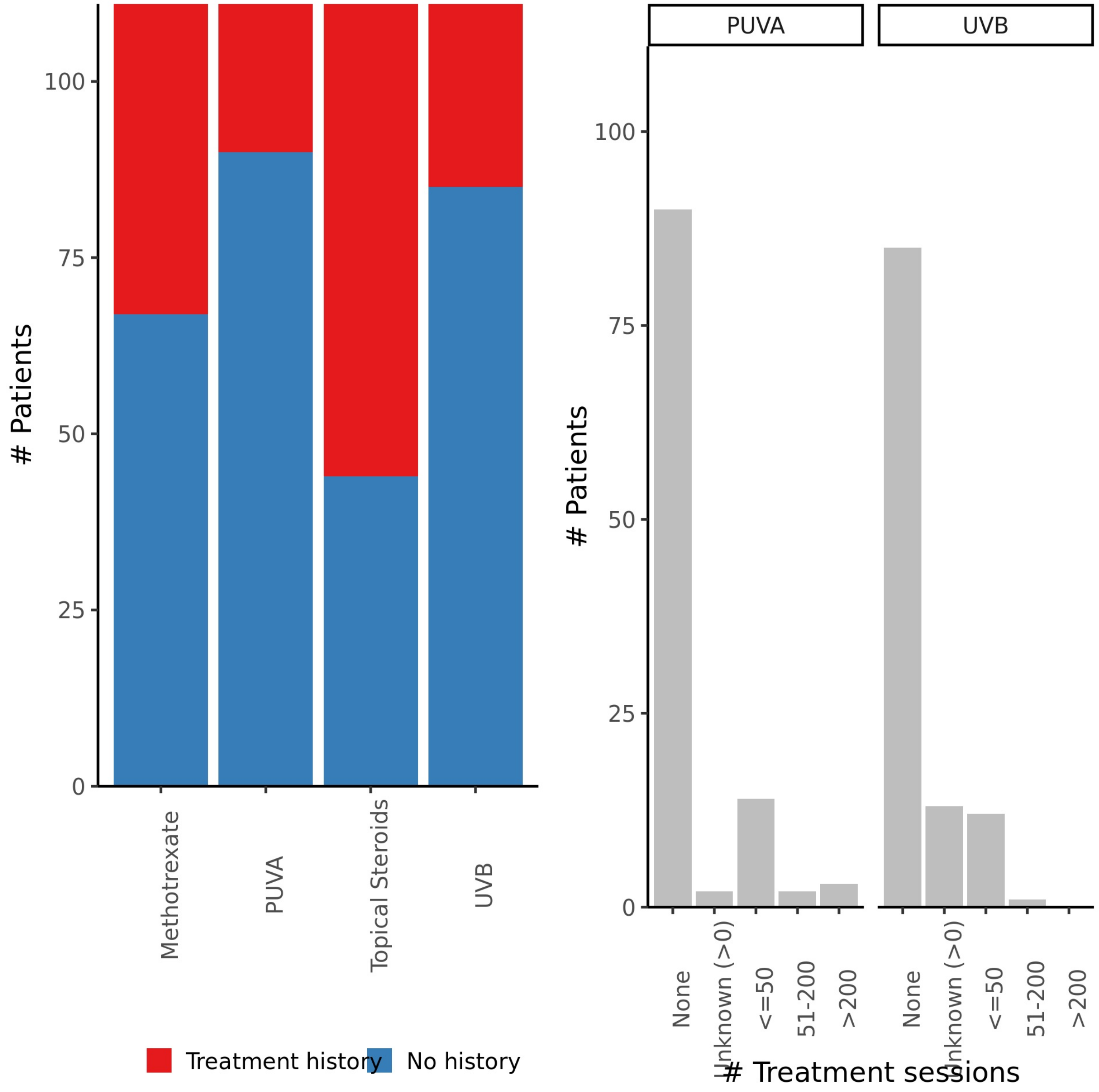

Effects of treatments on mutational spectra

Only a single unknown mutational signature was found during mutational signature extraction. We attribute this to psoralen exposure. PUVA treatment is likely a significant source of psoralen exposure in psoriasis patients. That only a single novel mutational signature was found is probably the strongest indication that other treatments don't affect the mutation spectra. However, to look further into the potential effects of the different treatments, I am going to compare the mutational spectra of patients who have or have not received the treatments. I want to pool the mutations from patients who have received different treatments. To keep any individuals with high mutation burdens from dominating the spectra though, I'm sampling 300 mutations from each patient.

PUVA

We can see the effects of the Psoralen signature on the mutation spectrum of patients with history of PUVA treatment.

```
all_mutations$puva_history <- ifelse(all_mutations$patient %in% patient_meta$Patient.ID[patient_meta$EverPuva], "
PUVA-History", "No-PUVA-History")

random_mutations <- all_mutations %>% group_by(patient) %>% sample_n(size = 300)

grange_obj = makeGRangesListFromDataFrame(random_mutations, split.field = "puva_history", keep.extra.columns = T,
ignore.strand = T, seqnames.field = "chr",
                                                start.field = "pos", end.field = "pos")

GenomeInfoDb::genome(grange_obj) = "hg38"
subs_grl <- get_mut_type(grange_obj)
mut_mat <- mut_matrix(subs_grl, ref_genome)

plot_96_profile(mut_mat)
```

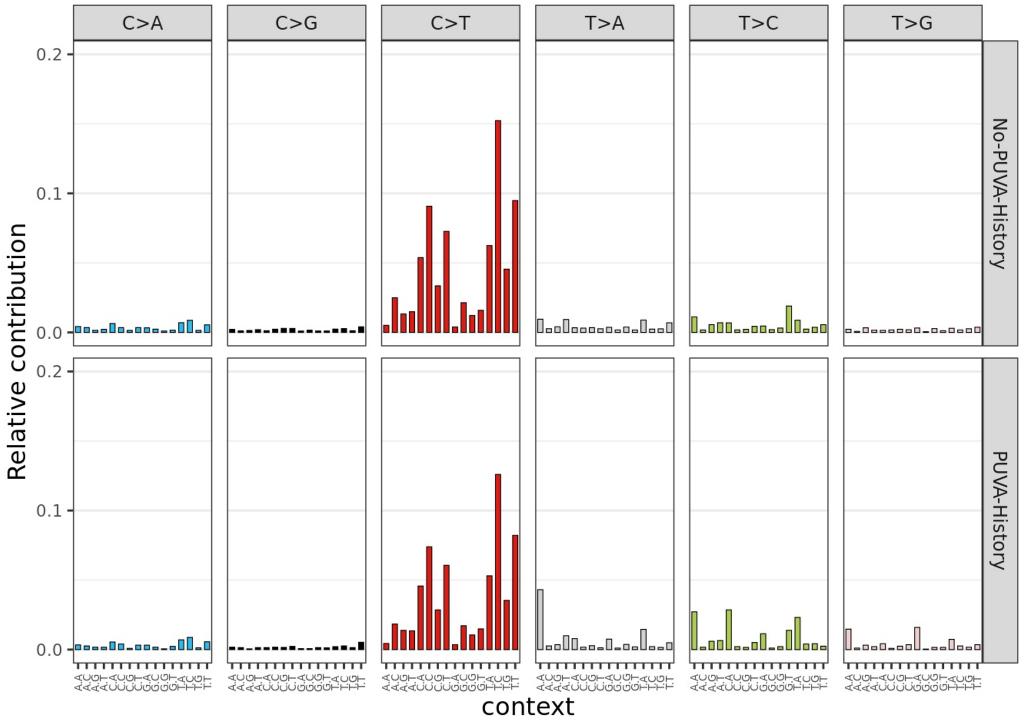


```
cos_sim(mut_mat[,1], mut_mat[,2])
```

```
## [1] 0.9677008
```

UV-B

Plot the effects of UV-B on the mutational spectrum. UV-B might be especially likely to affect the burden of the UV-signature SBS7, which is the dominant mutational signature in the dataset. We formally test for an effect of this treatment on SBS7 burden below but here show that the mutational spectra of UV-B treated and untreated patients look nearly identical. In particular, there is no hint of a difference in the C>T class.

```
all_mutations$uvb_history <- ifelse(all_mutations$patient %in% patient_meta$Patient.ID[patient_meta$EverUVB], "UV
B-History", "No-UVB-History")

random_mutations <- all_mutations %>% group_by(patient) %>% sample_n(size = 300)
grange_obj = makeGRangesListFromDataFrame(all_mutations, split.field = "uvb_history", keep.extra.columns = T, ign
ore.strand = T, seqnames.field = "chr",
                                                start.field = "pos", end.field = "pos")

GenomeInfoDb::genome(grange_obj) = "hg38"
subs_grl <- get_mut_type(grange_obj)
mut_mat <- mut_matrix(subs_grl, ref_genome)

plot_96_profile(mut_mat)
```

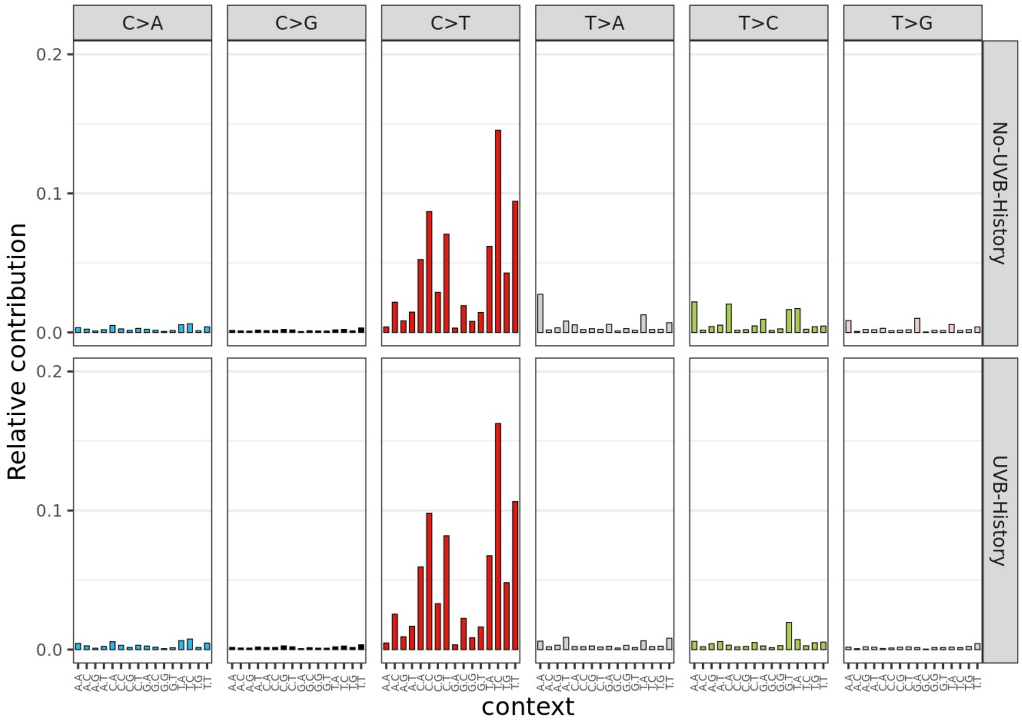


```
cos_sim(mut_mat[,1], mut_mat[,2])
```

```
## [1] 0.98688
```

Methotrexate

Methotrexate is a potentially mutagenic treatment. Below I compare the mutational spectra of patients with a history of methotrexate treatment with those that have no history of methotrexate treatment. The only differences between the two mutational spectra seem to be those associated with PUVA treatment. This might suggest that methotrexate use is correlated with PUVA treatment but that methotrexate itself has little or no effect on the mutational spectrum.

```
all_mutations$methotrexate_history <- ifelse(all_mutations$patient %in% patient_meta$Patient.ID[patient_meta$Ever
Methotrexate], "Methotrexate-History", "No-Methotrexate-History")
random_mutations <- all_mutations %>% group_by(patient) %>% sample_n(size = 300)

grange_obj = makeGRangesListFromDataFrame(all_mutations, split.field = "methotrexate_history", keep.extra.columns
= T, ignore.strand = T, seqnames.field = "chr",
                                                start.field = "pos", end.field = "pos")

GenomeInfoDb::genome(grange_obj) = "hg38"
subs_grl <- get_mut_type(grange_obj)
mut_mat <- mut_matrix(subs_grl, ref_genome)

plot_96_profile(mut_mat)
```

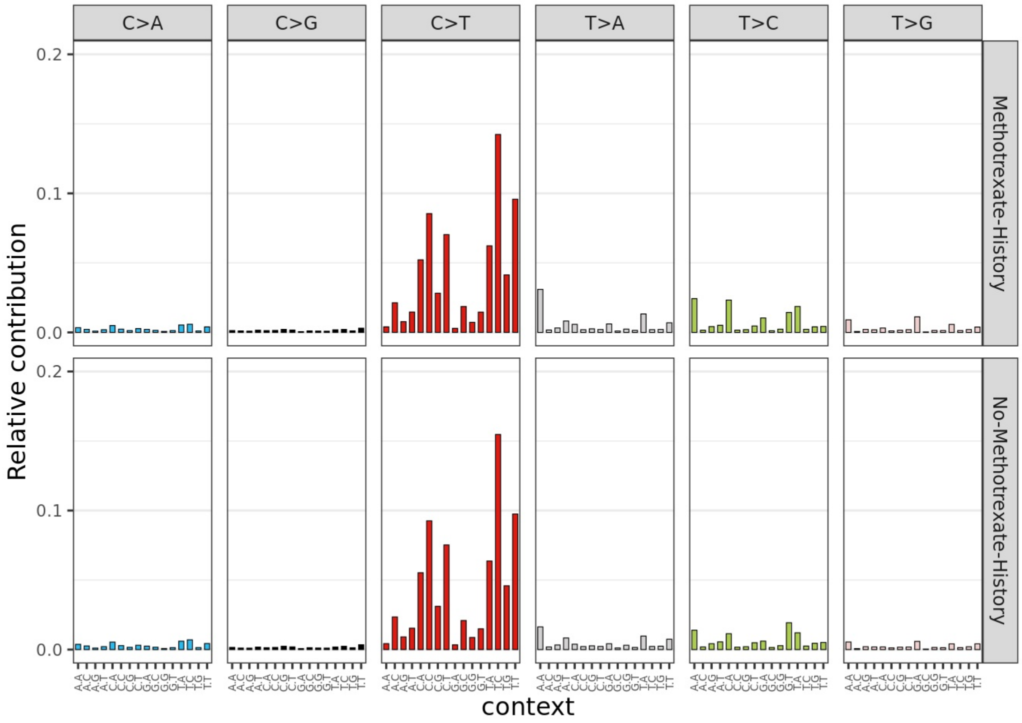


```
cos_sim(mut_mat[,1], mut_mat[,2])
```

```
## [1] 0.9933854
```

Topical steroids

Compare the mutational spectra of patients with and without a history of topical steroid use.

```
all_mutations$steroid_history <- ifelse(all_mutations$patient %in% patient_meta$Patient.ID[patient_meta$EverStero
ids], "Topical-Steroid-History", "No-topical-Steroid-History")
random_mutations <- all_mutations %>% group_by(patient) %>% sample_n(size = 300)

grange_obj = makeGRangesListFromDataFrame(all_mutations, split.field = "steroid_history", keep.extra.columns = T,
ignore.strand = T, seqnames.field = "chr",
                                                start.field = "pos", end.field = "pos")

GenomeInfoDb::genome(grange_obj) = "hg38"
subs_grl <- get_mut_type(grange_obj)
mut_mat <- mut_matrix(subs_grl, ref_genome)

plot_96_profile(mut_mat)
```

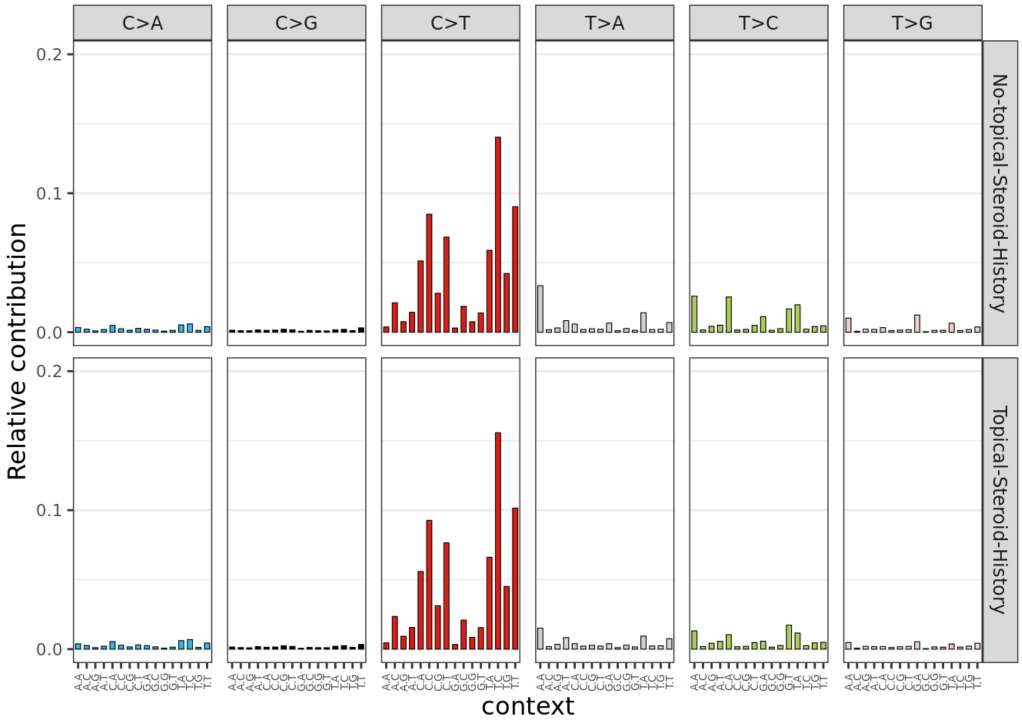

```
cos_sim(mut_mat[,1], mut_mat[,2])
```

```
## [1] 0.9896338
```

Effect of treatments on mutation burden of particular signatures

Another way to test for potential effects of treatments is to include the treatment status or dose in the linear models of mutation burden of different signatures. We can then test if including these variables improves the fit of the model.

```
working_dir="/nfs/users/nfs_s/so11/phd/psoriasis/bsub_jupyter_lab/psoriasis/manuscript_data_and_figures/Supplementary_material/"

clone_burden <- read.table(paste(working_dir, "Supplementary_Table3_clone_mutationBurden.txt", sep=""), h=T)
clone_burden <- merge(clone_burden, microd_meta[,c("SampleID", "BiopsyID", "MetaLocation",
                                                    "PatientID", "DiseaseStatus")], by.x="HighCellFrac_sample", by.y="SampleID")
clone_burden <- merge(clone_burden, patient_meta[,c("Patient.ID", "Age_at_sampling", "Disease_duration",
                                                  "Sex", "BMI", "Smoking", "PASI")], by.x="PatientID", by.y="Patient.ID")

clone_burden$Disease_duration[clone_burden$DiseaseStatus=="Non-lesional" & !is.na(clone_burden$Disease_duration)] <- 0

clone_burden <- merge(clone_burden, patient_meta[,c(1,22:27)], by.y="Patient.ID", by.x="PatientID")
clone_burden$AmountUVB <- factor(clone_burden$AmountUVB, levels=c("None","unknown", "<=50","51-200"))
clone_burden$AmountPuva <- factor(clone_burden$AmountPuva, levels=c("None","unknown", "<=50","51-200",">200"))
```

PUVA

The mutation burden of psoralen-associated mutations seems to increase with the number of PUVA cycles the patient has received. However, there are also some individuals who show a high burden of the psoralen signature without any history of PUVA treatment.

```
ggplot(clone_burden, aes(x=AmountPuva, y=PUVA)) + geom_boxplot()
```

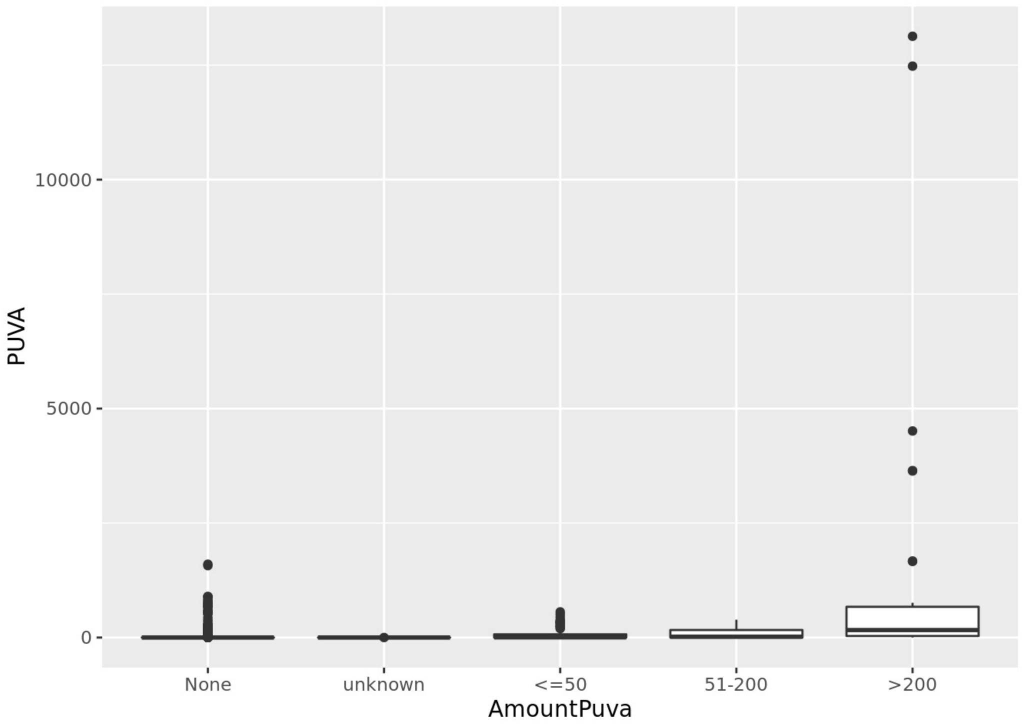


```
## Better to plot this on a log-scale
ggplot(clone_burden, aes(x=AmountPuva, y=PUVA+1, fill=AmountPuva))  +
geom_boxplot() +  theme_classic(base_size = BASESIZE) + scale_fill_brewer(palette="Set1") + theme(legend.position = "none") +
  labs(x="Number of PUVA cycles", y="Psoralen mutation burden") +
  scale_y_log10(breaks = trans_breaks("log10", function(x) 10^x),
                labels = trans_format("log10", math_format(10^.x)))
```

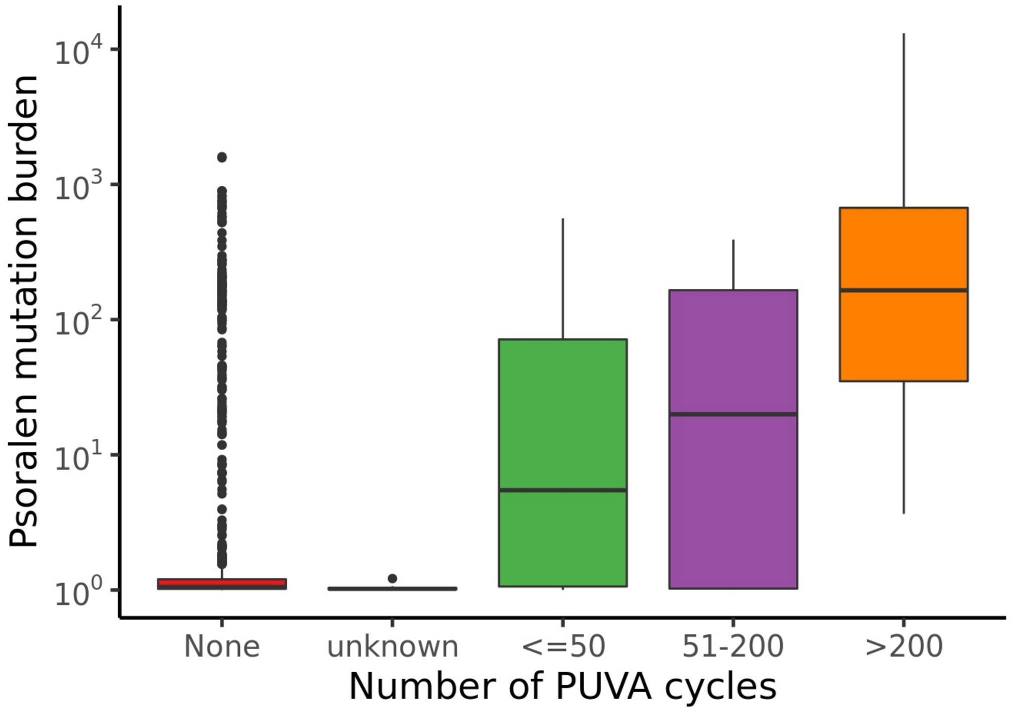


```
tapply(clone_burden$PUVA, clone_burden$AmountPuva, median, na.rm=T)
```

```
##         None      unknown         <=50       51-200         >200
##   0.05257151   0.02121345   4.47220392  18.94403196 163.64534000
```

UV-B

For UV-B, the story is less clear. Only a single individual has had >50 cycles of UV-B. This individual does show a higher-than-average burden of UV-related mutations but this is not sufficient for us to make any definitive statements about the mutagenic nature of this treatment.

```
ggplot(clone_burden, aes(x=AmountUVB, y=SBS7b, fill=AmountUVB))+
geom_boxplot() +  theme_classic(base_size = BASESIZE) + scale_fill_brewer(palette="Dark2") + theme(legend.positio
n = "none") +
  labs(x="Number of UV-B cycles", y="SBS7b mutation burden")
```

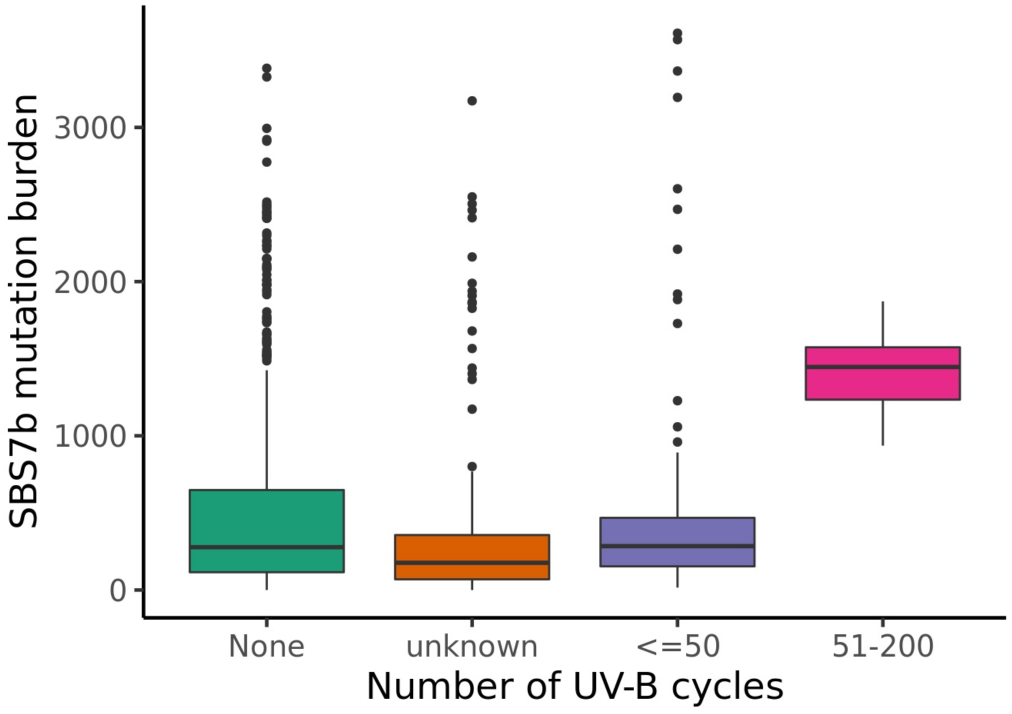


```
ggplot(clone_burden, aes(x=AmountUVB, y=SBS7b, fill=AmountUVB)) + geom_violin() +
  geom_boxplot(width=0.1) + theme_classic(base_size = BASESIZE) + scale_fill_brewer(palette="Dark2") + theme(lege
nd.position = "none") +
  labs(x="Number of UV-B cycles", y="SBS7b mutation burden")
```

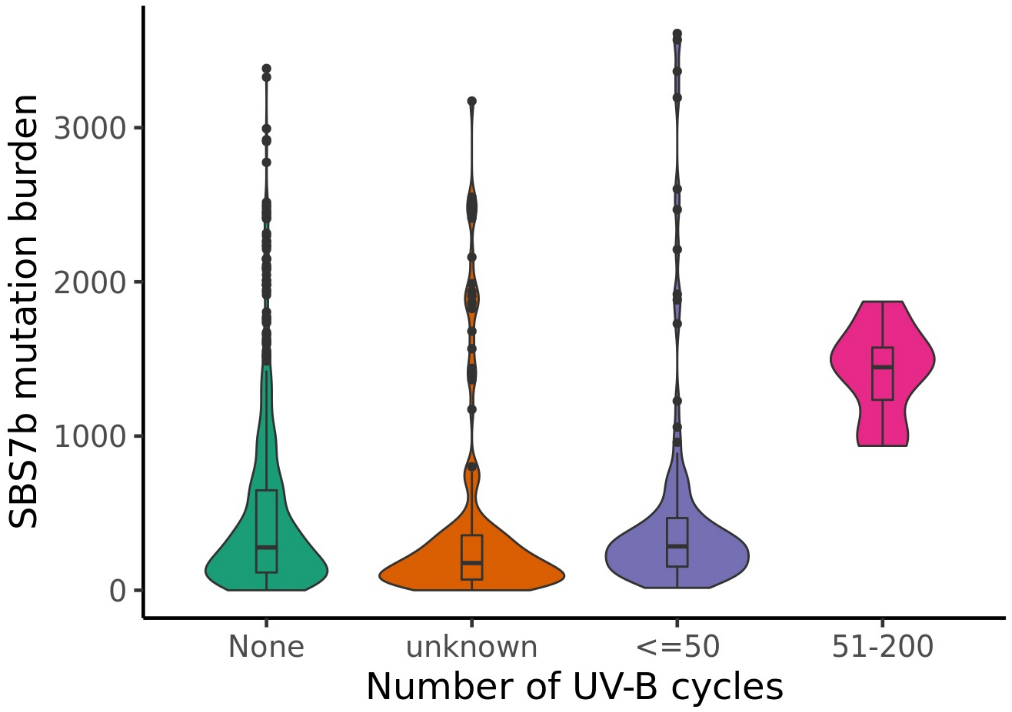


```
tapply(clone_burden$SBS7b, clone_burden$AmountUVB, median, na.rm=T)
```

```
##      None   unknown      <=50    51-200
##  277.6872  176.6552  284.5389 1446.3273
```

Methotrexate

History of treatment with methotrexate is not associated with increased mutation burden, neither of the UV-related SBS7 nor of the clock-like SBS1/5.

```
ggplot(clone_burden, aes(x=EverMethotrexate, y=SBS7b, fill=EverMethotrexate))+
geom_boxplot() +  theme_classic(base_size = BASESIZE) + scale_fill_brewer(palette="Dark2") + theme(legend.positio
n = "none") +
  labs(x="History of Methotrexate use", y="SBS7b mutation burden")
```

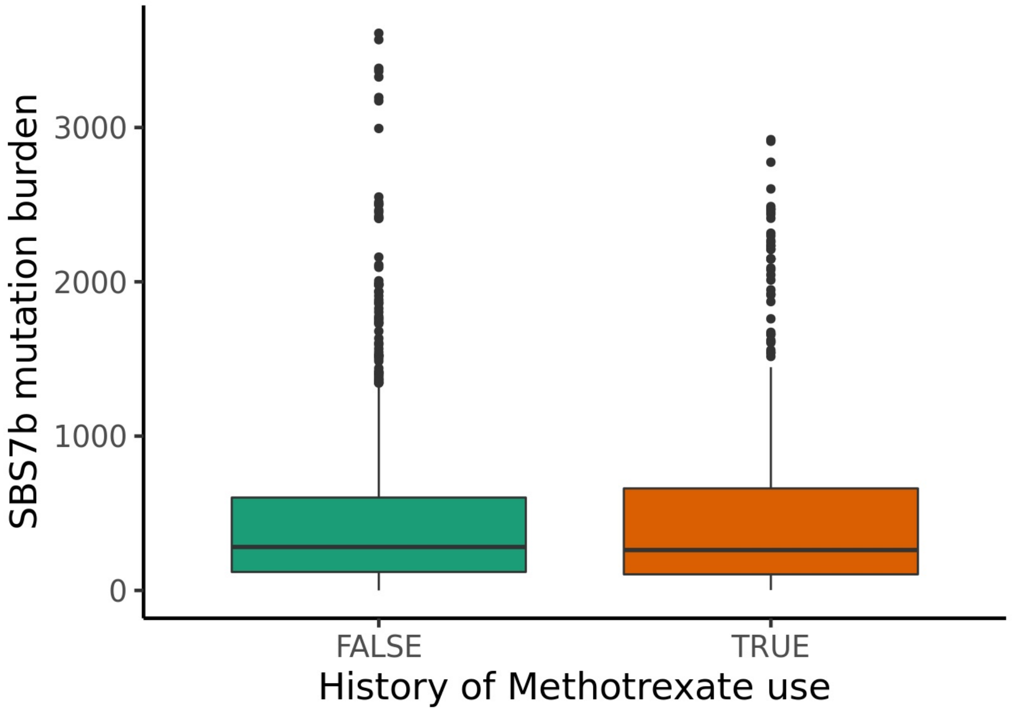


```
ggplot(clone_burden, aes(x=EverMethotrexate, y=SBS1.5, fill=EverMethotrexate))+
geom_boxplot() +  theme_classic(base_size = BASESIZE) + scale_fill_brewer(palette="Dark2") + theme(legend.positio
n = "none") +
  labs(x="History of Methotrexate use", y="SBS1/5 mutation burden")
```

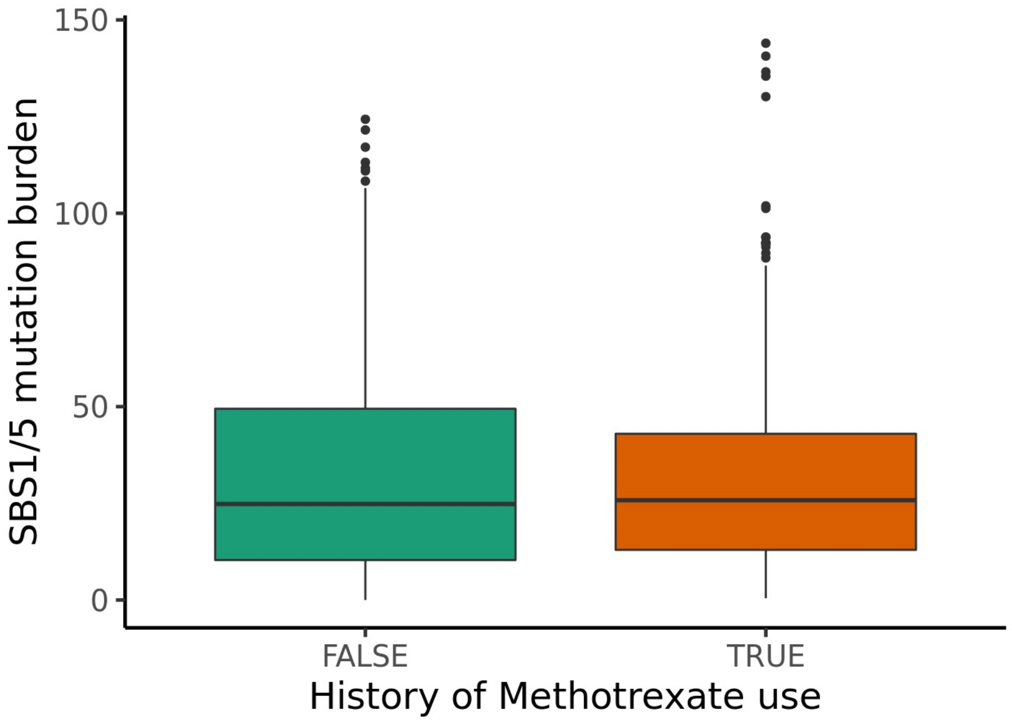

Topical steroids

History of treatment with topical steroids is not associated with increased mutation burden, neither of the UV-related SBS7 nor of the clock-like SBS1/5.

```
ggplot(clone_burden, aes(x=EverSteroids, y=SBS7b, fill=EverSteroids))+
geom_boxplot() +  theme_classic(base_size = BASESIZE) + scale_fill_brewer(palette="Dark2") + theme(legend.positio
n = "none") +
  labs(x="History of Methotrexate use", y="SBS7b mutation burden")
```

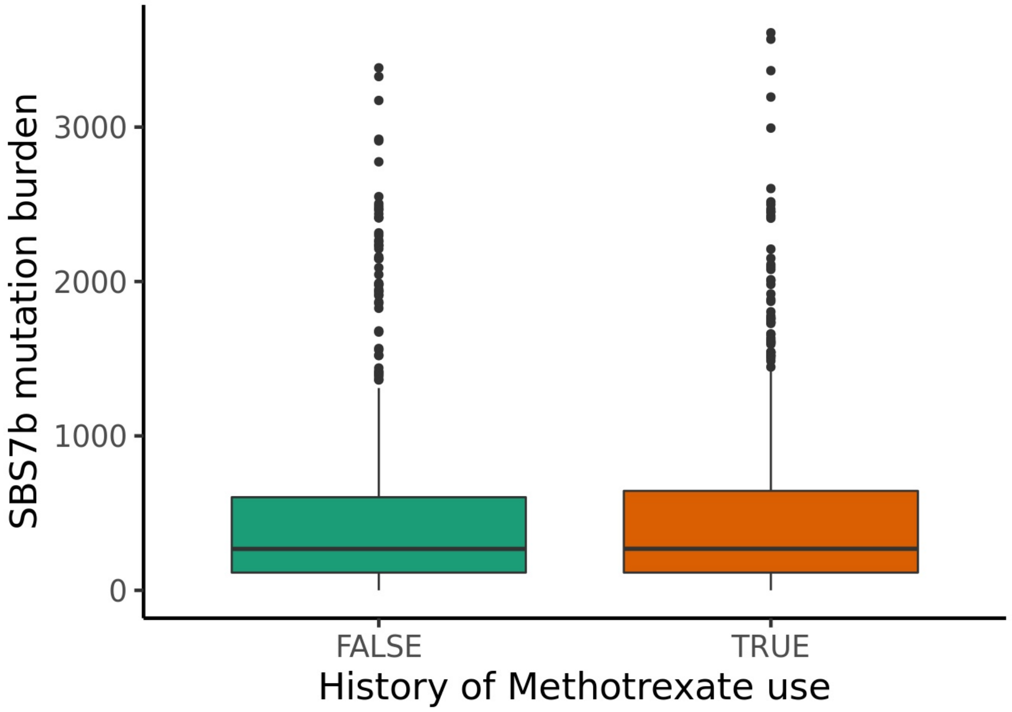


```
ggplot(clone_burden, aes(x=EverSteroids, y=SBS1.5, fill=EverSteroids))+
geom_boxplot() +  theme_classic(base_size = BASESIZE) + scale_fill_brewer(palette="Dark2") + theme(legend.positio
n = "none") +
  labs(x="History of Methotrexate use", y="SBS1/5 mutation burden")
```

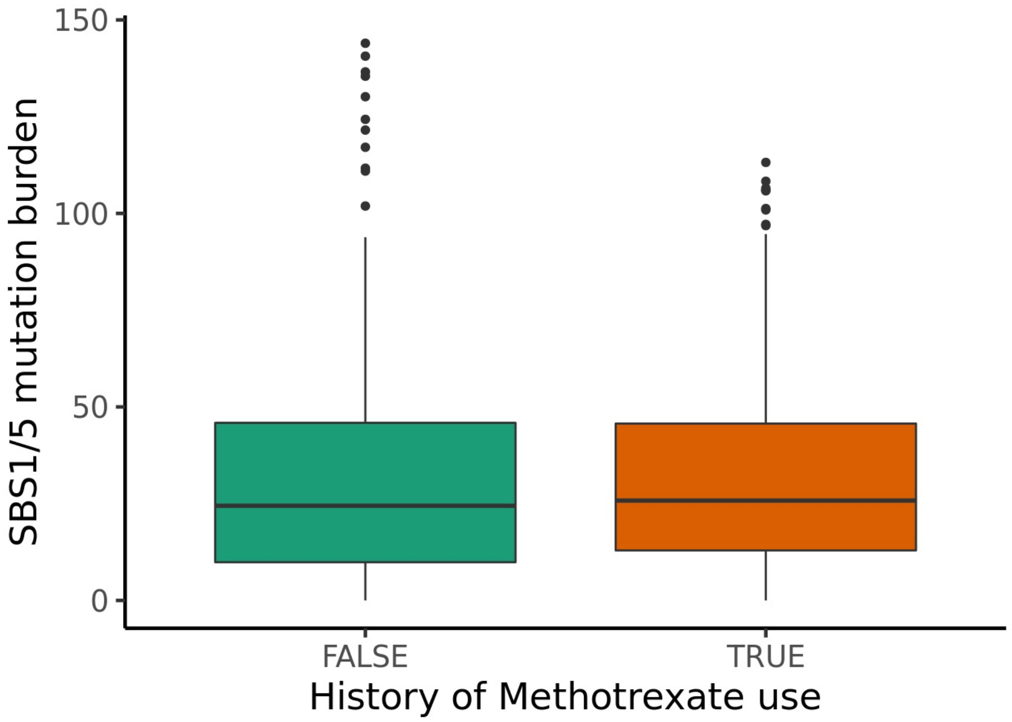

Effects of treatments on clonal composition of the tissue

```
microd_meta <- merge(microd_meta, patient_meta[, c("Patient.ID","Shows_Psoralen_signature","EverPuva","AmountPuva
","EverUVB","AmountUVB","EverMethotrexate","EverSteroids")], by.x="PatientID", by.y="Patient.ID", all.x=T)

microd_meta$AmountUVB <- factor(microd_meta$AmountUVB, levels=c("None","unknown", "<=50","51-200"))
microd_meta$AmountPuva <- factor(microd_meta$AmountPuva, levels=c("None","unknown", "<=50","51-200",">200"))

mVAFs <- data.frame(tapply(microd_meta$MedianVAF, microd_meta$PatientID, median, na.rm=T))
mVAFs$PatientID <- rownames(mVAFs)
colnames(mVAFs)[1] <- "MedianVAF"
patient_meta <- merge(patient_meta, mVAFs, by.x="Patient.ID", by.y="PatientID")
```

PUVA

There are a few ways to look at the effects of treatment on the clonal structure of the tissue. We could do a simple Wilcox test to check for higher VAFs among microbiopsies that are dissected from patients who have a history of PUVA treatment.

```
puva_vaf_test <- wilcox.test(microd_meta$MedianVAF~microd_meta$EverPuva)
puva_vaf_test
```

```
##
##  Wilcoxon rank sum test with continuity correction
##
## data:  microd_meta$MedianVAF by microd_meta$EverPuva
## W = 104968, p-value = 0.005047
## alternative hypothesis: true location shift is not equal to 0
```

However, the Wilcox test assumes independent sampling, which we haven't got. Samples taken from the same biopsy and from the same patient are likely to be correlated. Furthermore, we must take into account that patients who have received PUVA treatment are older than patients with no history of treatment. Older age often translates to higher VAF.

One way around this is to just take the median VAF of all the microbiopsies from the patient and regress this against the patient age and whether or not the psoralen signature is observed.

```
ggplot(patient_meta, aes(x=Age_at_sampling, y=MedianVAF, colour=Shows_Psoralen_signature)) + geom_point() + geom_
smooth(method="lm") + theme_classic(base_size = 18) +
  labs(y="Median VAF \n of microbiopsies", x="Patient Age", colour="Psoralen Signature") +
  theme(legend.position = "top") + scale_colour_manual(values=c("#FCBF49", "#40476D"))
```

```
## `geom_smooth()` using formula 'y ~ x'
```

```
## Warning: Removed 5 rows containing non-finite values (stat_smooth).
```

```
## Warning: Removed 5 rows containing missing values (geom_point).
```

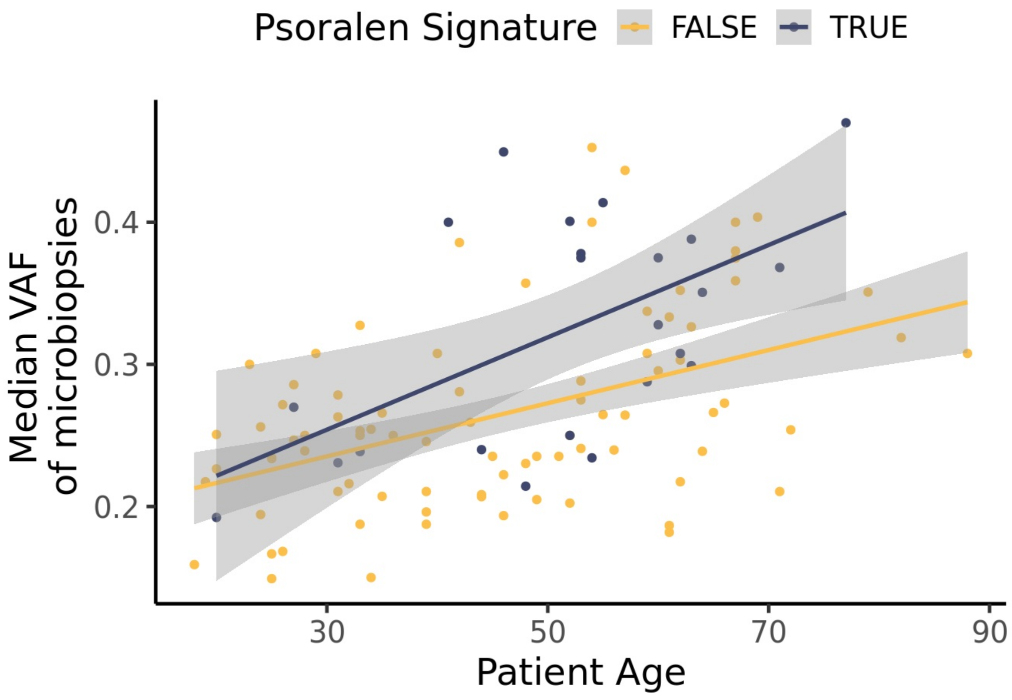


```
summary(lm(MedianVAF~Age_at_sampling + Shows_Psoralen_signature, data=patient_meta))
```

```
##
## Call:
## lm(formula = MedianVAF ~ Age_at_sampling + Shows_Psoralen_signature,
##     data = patient_meta)
##
## Residuals:
##       Min        1Q    Median        3Q       Max
## -0.114810 -0.047430  0.000359  0.039905  0.170611
##
## Coefficients:
##                              Estimate Std. Error t value Pr(>|t|)
## (Intercept)                 0.1691106  0.0183757   9.203 4.37e-15 ***
## Age_at_sampling             0.0020905  0.0003737   5.593 1.84e-07 ***
## Shows_Psoralen_signatureTRUE 0.0473183  0.0147209   3.214  0.00175 **
## ---
## Signif. codes:  0 '***' 0.001 '**' 0.01 '*' 0.05 '.' 0.1 ' ' 1
##
## Residual standard error: 0.06175 on 103 degrees of freedom
##   (5 observations deleted due to missingness)
## Multiple R-squared:  0.3186, Adjusted R-squared:  0.3053
## F-statistic: 24.08 on 2 and 103 DF,  p-value: 2.639e-09
```

Finally, we could do the same kind of linear-mixed-effects modeling as we've done for the mutation burden. We can use a likelihood ratio test to see if including a binary variable for the presence of the psoralen signature improves the fit of the model. We see that it does.

```
microd_meta <- merge(microd_meta, patient_meta, by.x="PatientID", by.y="Patient.ID")

model_vaf.null <- lme(fixed = MedianVAF.x ~ Age_at_sampling,
                      random = list(PatientID = pdSymm(form = ~ Age_at_sampling - 1), BiopsyID = pdSymm(form = ~
Age_at_sampling - 1)),
                      data = microd_meta[!is.na(microd_meta$Age_at_sampling) & !is.na(microd_meta$MedianVAF.x),],
method="ML")

model_vaf.psoralen <- lme(fixed = MedianVAF.x ~ Age_at_sampling + Shows_Psoralen_signature.x,
                      random = list(PatientID = pdSymm(form = ~ Age_at_sampling - 1), BiopsyID = pdSymm(form = ~
Age_at_sampling - 1)),
                      data = microd_meta[!is.na(microd_meta$Age_at_sampling) & !is.na(microd_meta$MedianVAF.x),],
method="ML")

anova(model_vaf.null,model_vaf.psoralen, test=T)$"p-value"[2]
```

```
## [1] 0.02836863
```

The above has simply tested a binary variable for whether or not the psoralen signature is observed. We can see however, that there does also seem to be a trend for increased clonality with greater number of PUVA cycles. This has the caveat of potentially different age distributions, as discussed above.

```
ggplot(microd_meta[!is.na(microd_meta$AmountPuva.x),], aes(x=AmountPuva.x, y=MedianVAF.x, fill=AmountPuva.x)) + g
eom_boxplot() +
  theme_classic(base_size = 18) +
  scale_fill_brewer(palette="Set1") + theme(legend.position = "none") +
  labs(x="Number of PUVA cycles", y="Median VAF of microbiopsy")
```

```
## Warning: Removed 6 rows containing non-finite values (stat_boxplot).
```

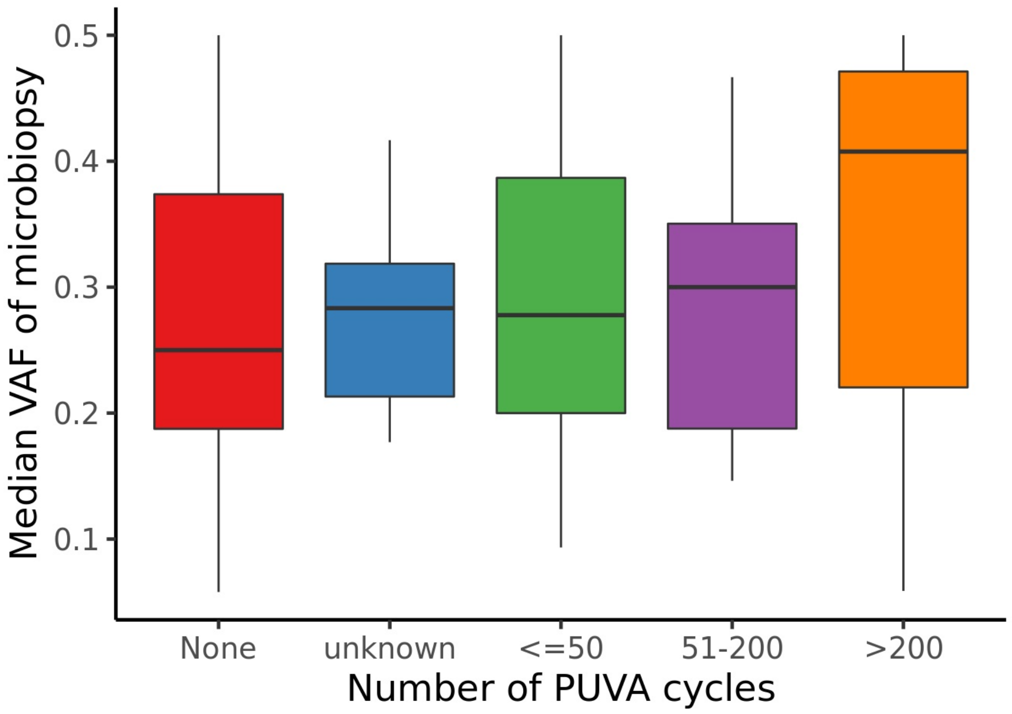

UVB

Is there evidence that treatment with UVB increases the VAFs of microbiopsies? It doesn't look like there is.

```
uvb_vaf_test <- wilcox.test(microd_meta$MedianVAF.x~microd_meta$EverUVB.x)
uvb_vaf_test
```

```
##
##  Wilcoxon rank sum test with continuity correction
##
## data:  microd_meta$MedianVAF.x by microd_meta$EverUVB.x
## W = 127561, p-value = 0.6868
## alternative hypothesis: true location shift is not equal to 0
```

```
summary(lm(MedianVAF~Age_at_sampling + EverUVB, data=patient_meta))
```

```
##
## Call:
## lm(formula = MedianVAF ~ Age_at_sampling + EverUVB, data = patient_meta)
##
## Residuals:
##       Min        1Q    Median        3Q       Max
## -0.124791 -0.047696 -0.002962  0.037881  0.177992
##
## Coefficients:
##                 Estimate Std. Error t value Pr(>|t|)
## (Intercept)     0.163842   0.020299   8.071 1.36e-12 ***
## Age_at_sampling 0.002340   0.000391   5.986 3.17e-08 ***
## EverUVBTRUE     0.015445   0.014754   1.047    0.298
## ---
## Signif. codes:  0 '***' 0.001 '**' 0.01 '*' 0.05 '.' 0.1 ' ' 1
##
## Residual standard error: 0.06444 on 103 degrees of freedom
##   (5 observations deleted due to missingness)
## Multiple R-squared:  0.2581, Adjusted R-squared:  0.2437
## F-statistic: 17.92 on 2 and 103 DF,  p-value: 2.103e-07
```

```
ggplot(microd_meta[!is.na(microd_meta$AmountUVB.x),], aes(x=AmountUVB.x, y=MedianVAF.x)) + geom_boxplot() +
  theme_classic(base_size = 18)
```

```
## Warning: Removed 6 rows containing non-finite values (stat_boxplot).
```

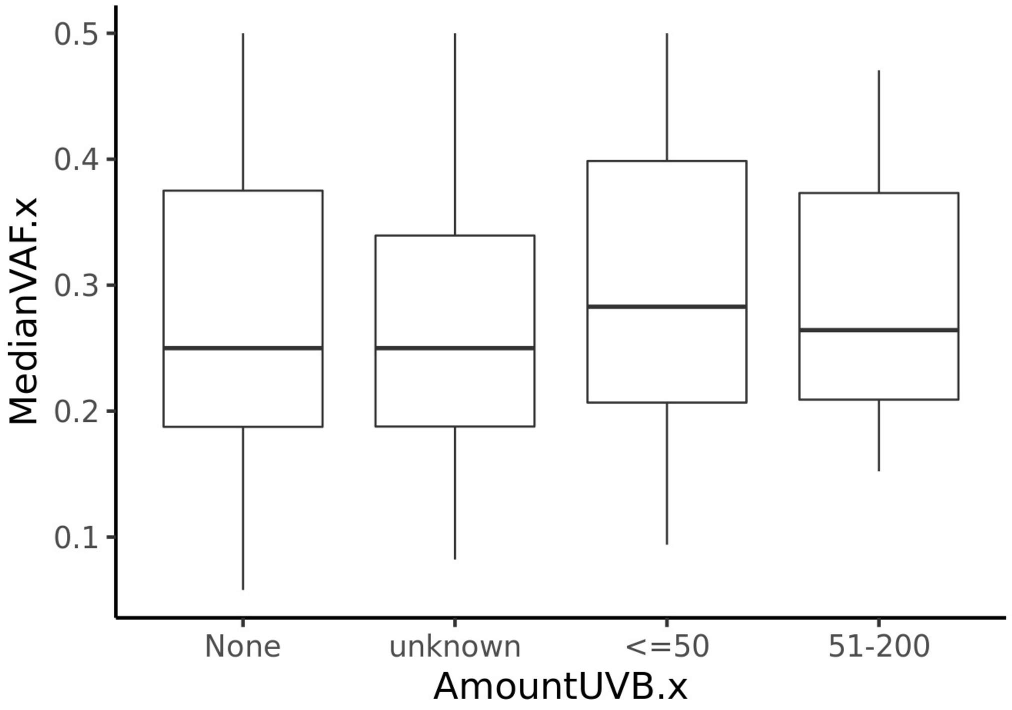

Methotrexate and topical steroids

The analysis for methotrexate and topical steroids was done in the same way as shown above for PUVA and UVB, but there was no evidence that these treatments affect the clonal structure of the epidermis.

Supplementary Note 4:

An R-markdown notebook describing the mutation burden analyses and models.

CodeS2: Burden and signature analyses

Sigurgeir Ólafsson

2/20/2022

Introduction

This document describes the clonality and burden analyses carried out as part of our manuscript “Effects of psoriasis and psoralen exposure on the somatic mutation landscape of the skin” by Sigurgeir Ólafsson et al.

This analysis uses pre-calculated summary statistics provided as supplementary tables. The mutation calls can be accessed from a Mendeley-Data repository that accompanies the manuscript and the raw sequencing data has also been made publicly available, please see the manuscript for details.

```
.libPaths("/lustre/scratch126/humgen/projects/psoriasis/R_packages_farm5_R4.1.0_install/")

library(ggplot2)
library(reshape2)
library(cowplot)
library(ggsignif)
library(nlme)

## DEFINE PLOTTING VARIABLES
#############################
BASESIZE=14

# Location colour vector
#Abdomen     Arm    Back   Flank     Leg
loc_colors <- c("#264653", "#2A9D8F","#E9C46A","#F4A261","#E76F51")

# Disease type (lesional vs non-lesional) colour vector
type_colours <- c("#FF7075", "#5DB4EA")

## Read in the meta-data
#############################

working_dir="/nfs/users/nfs_s/so11/phd/psoriasis/bsub_jupyter_lab/psoriasis/manuscript_data_and_figures/Supplemen
tary_material/"

microd_meta <- read.table(paste(working_dir, "Supplementary_Table2_microdissection_metadata.txt", sep=""), h=T)
patient_meta <- read.table(paste(working_dir, "Supplementary_Table1_patient_metadata.txt", sep=""), h=T)

biopsy_meta <- unique(microd_meta[microd_meta$ExclusionCriteria=="PASS",c("BiopsyID", "MetaLocation", "DiseaseSta
tus")])
table(biopsy_meta$MetaLocation, biopsy_meta$DiseaseStatus, useNA="always")
```

```
##
##           Lesional Non-lesional <NA>
##   Abdomen        9            8    0
##   Arm           20           21    0
##   Back          32           27    0
##   Flank         22           25    0
##   Leg           28           25    0
##   <NA>           0            0    0
```

```
table(microd_meta$MetaLocation[microd_meta$ExclusionCriteria=="PASS"], microd_meta$DiseaseStatus[microd_meta$Excl
usionCriteria=="PASS"], useNA="always")
```

```
##
##           Lesional Non-lesional <NA>
##   Abdomen       70           20    0
##   Arm          176           45    0
##   Back         294           54    0
##   Flank        186           57    0
##   Leg          220           60    0
##   <NA>           0            0    0
```

```
table(patient_meta$Sex)
```

```
##
## Female   Male
##     23     88
```

Clonality analysis

First compare the median variant allele frequencies (VAFs) of microbiopsies derrived from lesional and non-lesional skin. We see that they are near identical. Most somatic mutations are heterozygous, so in a fully clonal sample we would expect the median VAF to be 0.5. Most microbiopsies are a mix of clones and have median VAFs lower than 0.5.

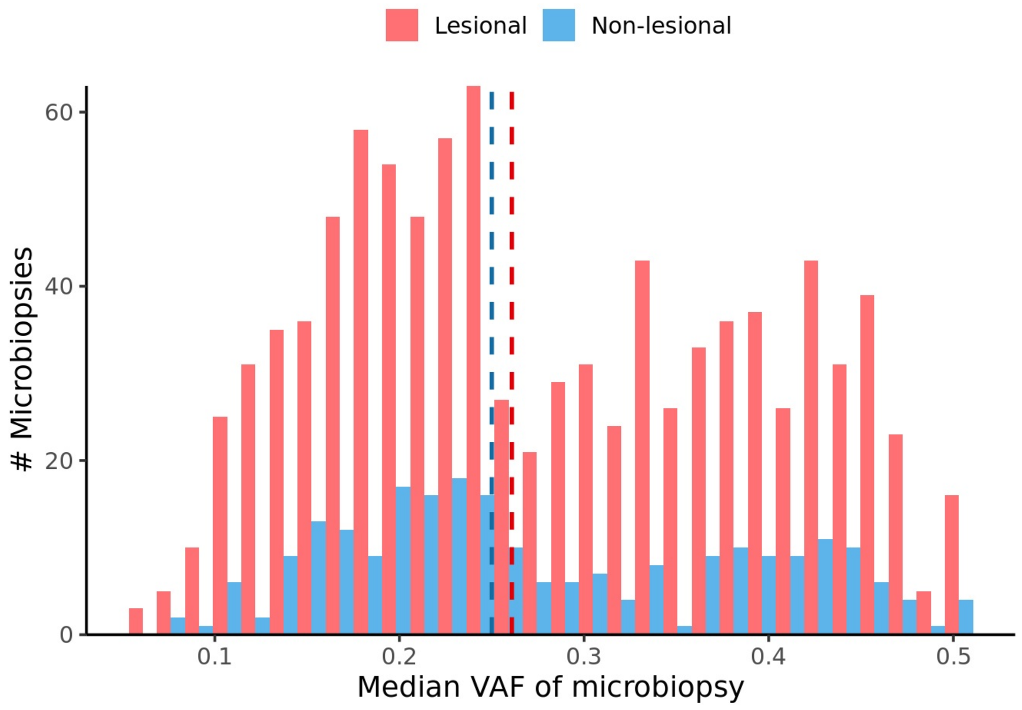

Mutation burden analyses

Since the microbiopsies tend to be a mix of cell clones, they don't give a good estimate of the per-cell mutation burden. Instead, I have computationally grouped mutations by their VAF into clusters. I have then used the pigeonhole principle to construct phylogenetic trees from the clusters. The mutation burden analyses are done on the level of the tips of the phylogenetic trees, which I refer to here as clones.

I have extracted mutational signatures for each cluster and computed the total mutation burden and the burden of each individual signature in each clone by summing accross the clusters. The scripts for doing each individual step are available on the Github page accompanying the manuscript but the below analyses will simply read in the results.

```
clone_burden <- read.table(paste(working_dir, "Supplementary_Table3_clone_mutationBurden.txt", sep=""), h=T)
clone_burden <- merge(clone_burden, microd_meta[,c("SampleID", "BiopsyID", "MetaLocation","PatientID", "DiseaseSt
atus")], by.x="HighCellFrac_sample", by.y="SampleID")
clone_burden <- merge(clone_burden, patient_meta[,c("Patient.ID", "Age_at_sampling", "Disease_duration","Sex")],
by.x="PatientID", by.y="Patient.ID")

clone_burden$Disease_duration[clone_burden$DiseaseStatus=="Non-lesional" & !is.na(clone_burden$Disease_duration)]
<- 0
```

Total mutation burden

We can first look at the total mutation burden as a function of age. Fit a linear mixed effects model with a fixed effects for age and the anatomical location of the sample and random effects for patient and for biopsy (nested within that of patient). Then add a fixed effect for disease duration (set to 0 for non-lesional samples, see above) and test if the fit of the model is improved using a likelihood ratio test.

```
model_null.lme <- lme(fixed = TotalSBS_adj ~ Age_at_sampling  + MetaLocation,
                      random = list(PatientID = pdSymm(form = ~ Age_at_sampling - 1), BiopsyID = pdSymm(form = ~
Age_at_sampling - 1)),
                      weights = varIdent(form= ~ 1 | DiseaseStatus),
                      data = clone_burden[!is.na(clone_burden$Disease_duration),], method="ML")

model_dur.lme <- lme(fixed = TotalSBS_adj ~ Age_at_sampling  + MetaLocation + Disease_duration,
                      random = list(PatientID = pdSymm(form = ~ Age_at_sampling - 1), BiopsyID = pdSymm(form = ~
Age_at_sampling - 1)),
                      weights = varIdent(form= ~ 1 | DiseaseStatus),
                      data = clone_burden[!is.na(clone_burden$Disease_duration),], method="ML")

summary(model_null.lme)
```

```
## Linear mixed-effects model fit by maximum likelihood
##   Data: clone_burden[!is.na(clone_burden$Disease_duration), ]
##        AIC      BIC    logLik
##   17032.8 17082.84 -8506.402
##
## Random effects:
##  Formula: ~Age_at_sampling - 1 | PatientID
##         Age_at_sampling
## StdDev:        7.680529
##
##  Formula: ~Age_at_sampling - 1 | BiopsyID %in% PatientID
##         Age_at_sampling Residual
## StdDev:        6.819941 1054.584
##
## Variance function:
##  Structure: Different standard deviations per stratum
##  Formula: ~1 | DiseaseStatus
##  Parameter estimates:
## Non-lesional     Lesional
##    1.0000000    0.3868207
## Fixed effects:  TotalSBS_adj ~ Age_at_sampling + MetaLocation
##                        Value Std.Error  DF   t-value p-value
## (Intercept)       -211.14474 163.37938 902 -1.292359  0.1966
## Age_at_sampling     17.32682   2.82333 100  6.137007  0.0000
## MetaLocationArm    125.51918 165.13669  92  0.760093  0.4491
## MetaLocationBack   -41.30589 156.24096  92 -0.264373  0.7921
## MetaLocationFlank  -58.97436 157.25017  92 -0.375035  0.7085
## MetaLocationLeg      2.50996 158.13177  92  0.015873  0.9874
##  Correlation:
##                   (Intr) Ag_t_s MtLctA MtLctB MtLctF
## Age_at_sampling   -0.586
## MetaLocationArm   -0.626 -0.040
## MetaLocationBack  -0.630 -0.096  0.683
## MetaLocationFlank -0.662 -0.035  0.679  0.722
## MetaLocationLeg   -0.630 -0.082  0.674  0.718  0.708
##
## Standardized Within-Group Residuals:
##         Min          Q1         Med          Q3         Max
## -4.98674062 -0.19899061 -0.04572419  0.18555987  8.88019700
##
## Number of Observations: 1100
## Number of Groups:
##               PatientID BiopsyID %in% PatientID
##                     102                     198
```

```
summary(model_dur.lme)
```

```
## Linear mixed-effects model fit by maximum likelihood
##   Data: clone_burden[!is.na(clone_burden$Disease_duration), ] 
##        AIC      BIC    logLik
##   17034.01 17089.04 -8506.003
## 
## Random effects:
##  Formula: ~Age_at_sampling - 1 | PatientID
##         Age_at_sampling
## StdDev:        7.831848
## 
##  Formula: ~Age_at_sampling - 1 | BiopsyID %in% PatientID
##         Age_at_sampling Residual
## StdDev:        6.686659 1052.549
## 
## Variance function:
##  Structure: Different standard deviations per stratum
##  Formula: ~1 | DiseaseStatus 
##  Parameter estimates:
## Non-lesional     Lesional 
##    1.0000000    0.3875551 
## Fixed effects:  TotalSBS_adj ~ Age_at_sampling + MetaLocation + Disease_duration 
##                        Value Std.Error  DF   t-value p-value
## (Intercept)       -208.45790 164.00277 902 -1.271063  0.2040
## Age_at_sampling     18.19676   3.00343 100  6.058664  0.0000
## MetaLocationArm    131.83600 165.93155  91  0.794520  0.4290
## MetaLocationBack   -32.95523 157.08583  91 -0.209791  0.8343
## MetaLocationFlank  -58.75449 157.84629  91 -0.372226  0.7106
## MetaLocationLeg      1.99564 158.75761  91  0.012570  0.9900
## Disease_duration    -2.78454   3.06423  91 -0.908725  0.3659
##  Correlation: 
##                   (Intr) Ag_t_s MtLctA MtLctB MtLctF MtLctL
## Age_at_sampling   -0.550                                   
## MetaLocationArm   -0.624 -0.024                            
## MetaLocationBack  -0.628 -0.072  0.684                     
## MetaLocationFlank -0.661 -0.033  0.678  0.721              
## MetaLocationLeg   -0.630 -0.077  0.674  0.717  0.708       
## Disease_duration  -0.012 -0.329 -0.043 -0.057  0.001 -0.003
## 
## Standardized Within-Group Residuals:
##         Min          Q1         Med          Q3         Max 
## -4.98429369 -0.20933691 -0.05395355  0.18416288  8.87382155 
## 
## Number of Observations: 1100
## Number of Groups: 
##               PatientID BiopsyID %in% PatientID 
##                     102                     198
```

```
anova(model_null.lme,model_dur.lme, test=T)$"p-value"[2]
```

```
## [1] 0.3715017
```

```
lme.ints <- intervals(model_null.lme, which="fixed")$fixed
lme.ints
```

```
##                       lower        est.     upper
## (Intercept)       -530.9170 -211.144742 108.62753
## Age_at_sampling     11.7407   17.326816  22.91293
## MetaLocationArm   -201.5608  125.519177 452.59920
## MetaLocationBack  -350.7665  -41.305890 268.15468
## MetaLocationFlank -370.4338  -58.974359 252.48512
## MetaLocationLeg   -310.6957    2.509962 315.71559
## attr(,"label")
## [1] "Fixed effects:"
```

We can plot the mutation burden as a function of the age of the patient. We note that there are huge outliers in the data. These are driven by a few clones having very high burden of the psoralen signature. The burden of the psoralen signature is not expected to increase linearly with age and so we wish to remove those mutations before proceeding further.

```
maxAge=max(clone_burden$Age_at_sampling)
ageEff=lme.ints["Age_at_sampling", "est."]
low <-lme.ints["Age_at_sampling", "lower"]
upp <- lme.ints["Age_at_sampling", "upper"]

ggplot(clone_burden, aes(y=TotalSBS_adj, x=Age_at_sampling, fill=DiseaseStatus)) + geom_point( colour="black", sh
ape=21) +
  scale_fill_manual(values=type_colours)  +
  labs(y="Total SBS", x="Age at sample donation") +
  theme_classic() + theme(legend.title = element_blank(), legend.position = "top") +
  geom_ribbon(aes(ymin=Age_at_sampling*low, ymax=Age_at_sampling*upp, x=Age_at_sampling), alpha = 0.3, show.legen
d=F) +
  geom_line(aes(y=Age_at_sampling*ageEff, x=Age_at_sampling)) +
  guides(fill = guide_legend(override.aes = list(size=2.5)))
```

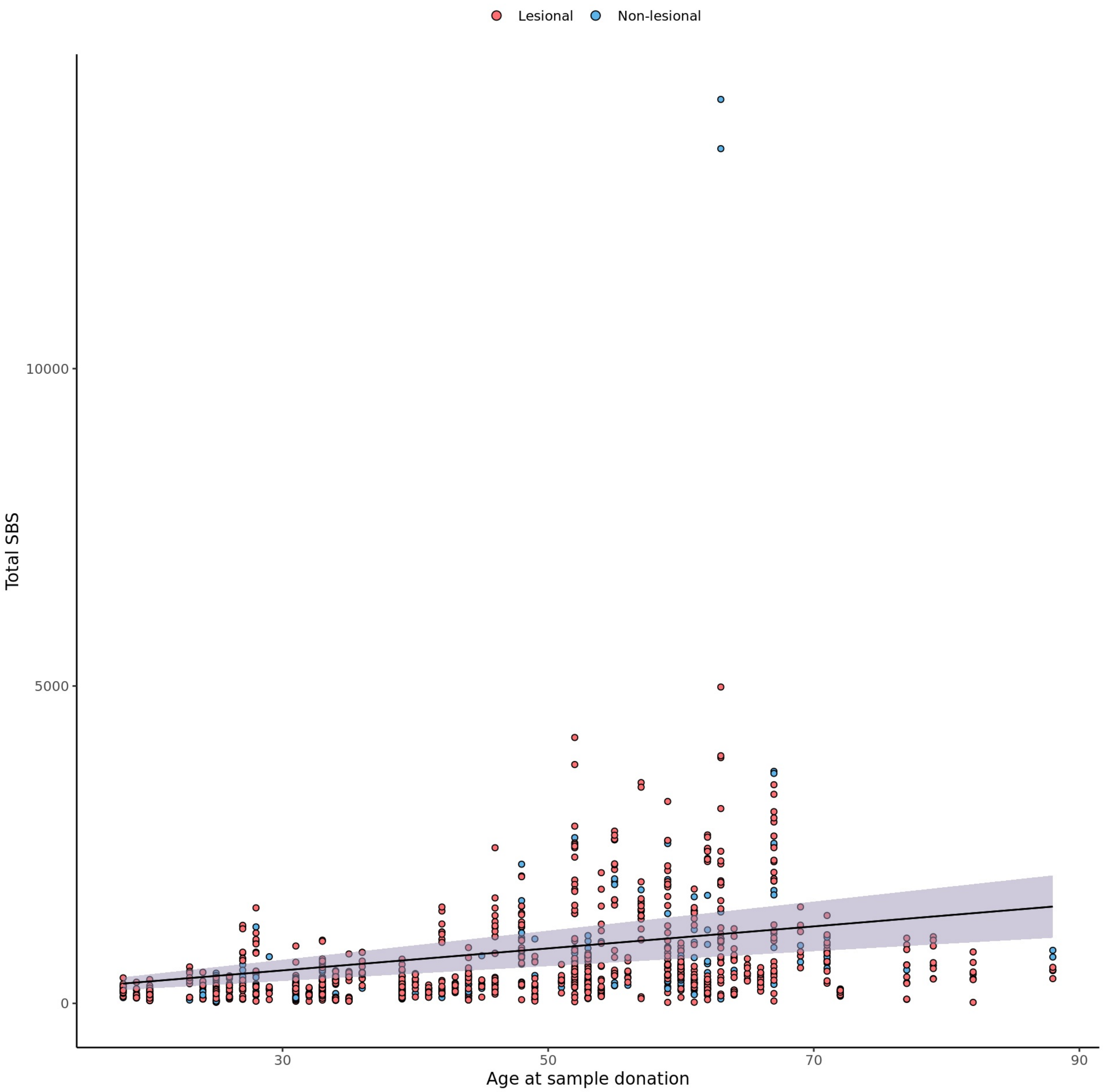

Total burden excluding Psoralen

The model above is affected by outlier samples which have a high burden of mutations attributed to the Psoralen signature. We'll get a more representative estimate of the rate at which mutations accumulate in the skin by excluding these.

There is still no significant effect of disease duration however.

```
clone_burden$noPUVA <- clone_burden$TotalSBS_adj - clone_burden$PUVA

model_noPUVA.null <- lme(fixed = noPUVA ~ Age_at_sampling  + MetaLocation,
                      random = list(PatientID = pdSymm(form = ~ Age_at_sampling - 1), BiopsyID = pdSymm(form = ~
Age_at_sampling - 1)),
                      weights = varIdent(form= ~ 1 | DiseaseStatus),
                      data = clone_burden[!is.na(clone_burden$Disease_duration),], method="ML")

model_noPUVA.dur <- lme(fixed = noPUVA ~ Age_at_sampling  + MetaLocation + Disease_duration,
                      random = list(PatientID = pdSymm(form = ~ Age_at_sampling - 1), BiopsyID = pdSymm(form = ~
Age_at_sampling - 1)),
                      weights = varIdent(form= ~ 1 | DiseaseStatus),
                      data = clone_burden[!is.na(clone_burden$Disease_duration),], method="ML")

summary(model_noPUVA.null)
```

```
## Linear mixed-effects model fit by maximum likelihood
##   Data: clone_burden[!is.na(clone_burden$Disease_duration), ]
##        AIC     BIC    logLik
##   16187.27 16237.3 -8083.634
##
## Random effects:
##  Formula: ~Age_at_sampling - 1 | PatientID
##         Age_at_sampling
## StdDev:        7.557317
##
##  Formula: ~Age_at_sampling - 1 | BiopsyID %in% PatientID
##         Age_at_sampling Residual
## StdDev:        3.651376 255.8907
##
## Variance function:
##  Structure: Different standard deviations per stratum
##  Formula: ~1 | DiseaseStatus
##  Parameter estimates:
## Non-lesional     Lesional
##     1.000000     1.321159
## Fixed effects:  noPUVA ~ Age_at_sampling + MetaLocation
##                       Value Std.Error  DF   t-value p-value
## (Intercept)       -165.31689 130.49624 902 -1.266833  0.2055
## Age_at_sampling     14.56302   2.26278 100  6.435883  0.0000
## MetaLocationArm    132.64561 129.52608  92  1.024084  0.3085
## MetaLocationBack    -0.87028 123.25857  92 -0.007061  0.9944
## MetaLocationFlank  -40.06695 122.94705  92 -0.325888  0.7452
## MetaLocationLeg     -8.44252 125.40753  92 -0.067321  0.9465
##  Correlation:
##                   (Intr) Ag_t_s MtLctA MtLctB MtLctF
## Age_at_sampling   -0.582
## MetaLocationArm   -0.646 -0.035
## MetaLocationBack  -0.649 -0.087  0.711
## MetaLocationFlank -0.674 -0.049  0.728  0.776
## MetaLocationLeg   -0.646 -0.073  0.696  0.746  0.740
##
## Standardized Within-Group Residuals:
##         Min          Q1         Med          Q3         Max
## -6.25854478 -0.25782381 -0.05141133  0.23651517  5.67628055
##
## Number of Observations: 1100
## Number of Groups:
##              PatientID BiopsyID %in% PatientID
##                    102                     198
```

```
noPUVA.ints <- intervals(model_noPUVA.null, which="fixed")$fixed
noPUVA.ints
```

```
##                        lower         est.     upper
## (Intercept)       -420.72904 -165.3168907  90.09525
## Age_at_sampling     10.08598   14.5630168  19.04006
## MetaLocationArm   -123.90183  132.6456056 389.19304
## MetaLocationBack  -245.00388   -0.8702803 243.26332
## MetaLocationFlank -283.58353  -40.0669454 203.44964
## MetaLocationLeg   -256.83249   -8.4425205 239.94745
## attr(,"label")
## [1] "Fixed effects:"
```

```
summary(model_noPUVA.dur)
```

```
## Linear mixed-effects model fit by maximum likelihood
##   Data: clone_burden[!is.na(clone_burden$Disease_duration), ]
##        AIC      BIC    logLik
##   16189.09 16244.12 -8083.545
##
## Random effects:
##  Formula: ~Age_at_sampling - 1 | PatientID
##         Age_at_sampling
## StdDev:        7.568998
##
##  Formula: ~Age_at_sampling - 1 | BiopsyID %in% PatientID
##         Age_at_sampling Residual
## StdDev:        3.635017 255.9926
##
## Variance function:
##  Structure: Different standard deviations per stratum
##  Formula: ~1 | DiseaseStatus
##  Parameter estimates:
## Non-lesional     Lesional
##     1.000000     1.320618
## Fixed effects:  noPUVA ~ Age_at_sampling + MetaLocation + Disease_duration
##                       Value Std.Error  DF   t-value p-value
## (Intercept)       -164.79375 130.64694 902 -1.261367  0.2075
## Age_at_sampling     14.71062   2.29255 100  6.416705  0.0000
## MetaLocationArm    132.08956 129.66993  91  1.018660  0.3111
## MetaLocationBack     0.91712 123.46018  91  0.007428  0.9941
## MetaLocationFlank  -41.15383 123.10015  91 -0.334312  0.7389
## MetaLocationLeg     -8.35641 125.54401  91 -0.066562  0.9471
## Disease_duration    -0.60387   1.43085  91 -0.422036  0.6740
##  Correlation:
##                   (Intr) Ag_t_s MtLctA MtLctB MtLctF MtLctL
## Age_at_sampling   -0.574
## MetaLocationArm   -0.646 -0.036
## MetaLocationBack  -0.649 -0.081  0.710
## MetaLocationFlank -0.674 -0.051  0.729  0.775
## MetaLocationLeg   -0.646 -0.072  0.696  0.746  0.740
## Disease_duration  -0.009 -0.153  0.007 -0.035  0.022 -0.001
##
## Standardized Within-Group Residuals:
##         Min          Q1         Med          Q3         Max
## -6.24342427 -0.25400058 -0.05201199  0.23939919  5.67895083
##
## Number of Observations: 1100
## Number of Groups:
##               PatientID BiopsyID %in% PatientID
##                     102                     198
```

```
intervals(model_noPUVA.dur, which="fixed")$fixed
```

```
##                         lower         est.      upper
## (Intercept)       -420.383954 -164.7937480  90.796458
## Age_at_sampling     10.176761   14.7106207  19.244481
## MetaLocationArm   -124.662960  132.0895617 388.842084
## MetaLocationBack  -243.539804    0.9171232 245.374050
## MetaLocationFlank -284.897878  -41.1538329 202.590212
## MetaLocationLeg   -256.939420   -8.3564142 240.226592
## Disease_duration    -3.437015   -0.6038699   2.229275
## attr(,"label")
## [1] "Fixed effects:"
```

```
anova(model_noPUVA.null,model_noPUVA.dur, test=T)$"p-value"[2]
```

```
## [1] 0.6732167
```

UV-associated mutation burden

UV-light is the dominant mutagen in the skin, accounting for 80% of the mutations in this dataset (and even more if PUVA isn’t considered). We may be interested in knowing the rate at which UV-associated mutations accumulate in the skin. Fit the model using only the mutation burden attributed to UV-related signatures.

```
clone_burden$UV <- clone_burden$SBS7b + clone_burden$SBS7c
model_UV.null <- lme(fixed = UV ~ Age_at_sampling  + MetaLocation,
                      random = list(PatientID = pdSymm(form = ~ Age_at_sampling - 1), BiopsyID = pdSymm(form = ~
Age_at_sampling - 1)),
                      weights = varIdent(form= ~ 1 | DiseaseStatus),
                      data = clone_burden[!is.na(clone_burden$Disease_duration),], method="ML")

model_UV.dur <- lme(fixed = UV ~ Age_at_sampling  + MetaLocation + Disease_duration,
                      random = list(PatientID = pdSymm(form = ~ Age_at_sampling - 1), BiopsyID = pdSymm(form = ~
Age_at_sampling - 1)),
                      weights = varIdent(form= ~ 1 | DiseaseStatus),
                      data = clone_burden[!is.na(clone_burden$Disease_duration),], method="ML")

summary(model_UV.null)
```

```
## Linear mixed-effects model fit by maximum likelihood
##   Data: clone_burden[!is.na(clone_burden$Disease_duration), ]
##       AIC      BIC    logLik
##   16147.1 16197.13 -8063.551
##
## Random effects:
##  Formula: ~Age_at_sampling - 1 | PatientID
##         Age_at_sampling
## StdDev:        7.647154
##
##  Formula: ~Age_at_sampling - 1 | BiopsyID %in% PatientID
##         Age_at_sampling Residual
## StdDev:        3.516487 252.6377
##
## Variance function:
##  Structure: Different standard deviations per stratum
##  Formula: ~1 | DiseaseStatus
##  Parameter estimates:
## Non-lesional     Lesional
##     1.000000     1.310479
## Fixed effects:  UV ~ Age_at_sampling + MetaLocation
##                       Value Std.Error  DF   t-value p-value
## (Intercept)       -175.84685 130.69328 902 -1.345493  0.1788
## Age_at_sampling     13.62039   2.27119 100  5.997037  0.0000
## MetaLocationArm    132.80864 129.69847  92  1.023980  0.3085
## MetaLocationBack     6.22690 123.36307  92  0.050476  0.9599
## MetaLocationFlank  -40.40738 123.03066  92 -0.328433  0.7433
## MetaLocationLeg     -1.47761 125.60020  92 -0.011764  0.9906
##  Correlation:
##                   (Intr) Ag_t_s MtLctA MtLctB MtLctF
## Age_at_sampling   -0.582
## MetaLocationArm   -0.646 -0.036
## MetaLocationBack  -0.650 -0.088  0.712
## MetaLocationFlank -0.674 -0.050  0.731  0.780
## MetaLocationLeg   -0.646 -0.074  0.697  0.747  0.741
##
## Standardized Within-Group Residuals:
##         Min          Q1         Med          Q3         Max
## -6.42862268 -0.25457082 -0.05763308  0.22646220  5.71098792
##
## Number of Observations: 1100
## Number of Groups:
##               PatientID BiopsyID %in% PatientID
##                     102                     198
```

```
summary(model_UV.dur)
```

```
## Linear mixed-effects model fit by maximum likelihood
##   Data: clone_burden[!is.na(clone_burden$Disease_duration), ]
##        AIC      BIC    logLik
##   16148.78 16203.81 -8063.388
##
## Random effects:
##  Formula: ~Age_at_sampling - 1 | PatientID
##         Age_at_sampling
## StdDev:        7.663792
##
##  Formula: ~Age_at_sampling - 1 | BiopsyID %in% PatientID
##         Age_at_sampling Residual
## StdDev:        3.491846 252.7528
##
## Variance function:
##  Structure: Different standard deviations per stratum
##  Formula: ~1 | DiseaseStatus
##  Parameter estimates:
## Non-lesional     Lesional
##     1.000000     1.309867
## Fixed effects:  UV ~ Age_at_sampling + MetaLocation + Disease_duration
##                       Value Std.Error  DF   t-value p-value
## (Intercept)       -175.13082 130.87983 902 -1.338104  0.1812
## Age_at_sampling     13.81551   2.30035 100  6.005818  0.0000
## MetaLocationArm    131.97649 129.87536  91  1.016178  0.3122
## MetaLocationBack     8.64571 123.59234  91  0.069953  0.9444
## MetaLocationFlank  -41.87075 123.20960  91 -0.339834  0.7348
## MetaLocationLeg     -1.31038 125.76835  91 -0.010419  0.9917
## Disease_duration    -0.79604   1.39212  91 -0.571817  0.5689
##  Correlation:
##                   (Intr) Ag_t_s MtLctA MtLctB MtLctF MtLctL
## Age_at_sampling   -0.574
## MetaLocationArm   -0.646 -0.037
## MetaLocationBack  -0.649 -0.081  0.711
## MetaLocationFlank -0.674 -0.053  0.731  0.779
## MetaLocationLeg   -0.645 -0.073  0.697  0.747  0.741
## Disease_duration  -0.008 -0.149  0.008 -0.035  0.022 -0.002
##
## Standardized Within-Group Residuals:
##        Min         Q1        Med         Q3        Max
## -6.4086116 -0.2510775 -0.0546847  0.2264474  5.7143715
##
## Number of Observations: 1100
## Number of Groups:
##               PatientID BiopsyID %in% PatientID
##                     102                     198
```

```
anova(model_UV.null,model_UV.dur, test=T)$"p-value"[2]
```

```
## [1] 0.5681442
```

```
UV.ints <- intervals(model_UV.null, which="fixed")$fixed
UV.ints
```

```
##                         lower        est.     upper
## (Intercept)       -431.644647 -175.846849  79.95095
## Age_at_sampling      9.126728   13.620392  18.11406
## MetaLocationArm   -124.080231  132.808635 389.69750
## MetaLocationBack  -238.113688    6.226904 250.56750
## MetaLocationFlank -284.089560  -40.407377 203.27481
## MetaLocationLeg   -250.249192   -1.477608 247.29398
## attr(,"label")
## [1] "Fixed effects:"
```

Again there is no significant effect of disease duration in this model.

SBS1/5 - associated mutation burden

The mutational signatures SBS1 and SBS5 are found in all normal cells at varying frequencies. They accumulate linearly with age but are accelerated in some inflamed tissues, including colonic mucosa affected by inflammatory bowel disease (see https://doi.org/10.1016/j.cell.2020.06.036 (https://doi.org/10.1016/j.cell.2020.06.036)). UV-exposure adds a lot of variance to the dataset and may mask any potential effects of psoriasis on the mutation burden. We should test if there is an effect of disease duration on the SBS1/5 mutation burden.

```
model_clock.null <- lme(fixed = SBS1.5 ~ Age_at_sampling  + MetaLocation,
                      random = list(PatientID = pdSymm(form = ~ Age_at_sampling - 1), BiopsyID = pdSymm(form = ~
Age_at_sampling - 1)),
                      weights = varIdent(form= ~ 1 | DiseaseStatus),
                      data = clone_burden[!is.na(clone_burden$Disease_duration),], method="ML")

model_clock.dur <- lme(fixed = SBS1.5 ~ Age_at_sampling  + MetaLocation + Disease_duration,
                      random = list(PatientID = pdSymm(form = ~ Age_at_sampling - 1), BiopsyID = pdSymm(form = ~
Age_at_sampling - 1)),
                      weights = varIdent(form= ~ 1 | DiseaseStatus),
                      data = clone_burden[!is.na(clone_burden$Disease_duration),], method="ML")

summary(model_clock.null)
```

```
## Linear mixed-effects model fit by maximum likelihood
##   Data: clone_burden[!is.na(clone_burden$Disease_duration), ]
##        AIC      BIC    logLik
##   9229.044 9279.075 -4604.522
##
## Random effects:
##  Formula: ~Age_at_sampling - 1 | PatientID
##         Age_at_sampling
## StdDev:        0.239352
##
##  Formula: ~Age_at_sampling - 1 | BiopsyID %in% PatientID
##         Age_at_sampling Residual
## StdDev:       0.1796447  11.3497
##
## Variance function:
##  Structure: Different standard deviations per stratum
##  Formula: ~1 | DiseaseStatus
##  Parameter estimates:
## Non-lesional     Lesional
##     1.000000     1.267614
## Fixed effects:  SBS1.5 ~ Age_at_sampling + MetaLocation
##                        Value Std.Error  DF   t-value p-value
## (Intercept)         4.076509  4.704412 902  0.866529  0.3864
## Age_at_sampling     0.690176  0.079826 100  8.646062  0.0000
## MetaLocationArm   -11.719469  4.672395  92 -2.508236  0.0139
## MetaLocationBack   -6.033234  4.457973  92 -1.353358  0.1793
## MetaLocationFlank  -5.090121  4.454521  92 -1.142687  0.2561
## MetaLocationLeg    -8.356679  4.511533  92 -1.852292  0.0672
##  Correlation:
##                   (Intr) Ag_t_s MtLctA MtLctB MtLctF
## Age_at_sampling   -0.584
## MetaLocationArm   -0.647 -0.028
## MetaLocationBack  -0.646 -0.084  0.703
## MetaLocationFlank -0.675 -0.036  0.712  0.753
## MetaLocationLeg   -0.649 -0.065  0.694  0.736  0.731
##
## Standardized Within-Group Residuals:
##        Min         Q1        Med         Q3        Max
## -5.8550103 -0.4753986 -0.1080387  0.4219794  4.2201898
##
## Number of Observations: 1100
## Number of Groups:
##               PatientID BiopsyID %in% PatientID
##                     102                     198
```

```
summary(model_clock.dur)
```

```
## Linear mixed-effects model fit by maximum likelihood
##   Data: clone_burden[!is.na(clone_burden$Disease_duration), ]
##        AIC      BIC    logLik
##   9224.689 9279.723 -4601.345
##
## Random effects:
##  Formula: ~Age_at_sampling - 1 | PatientID
##         Age_at_sampling
## StdDev:       0.2448763
##
##  Formula: ~Age_at_sampling - 1 | BiopsyID %in% PatientID
##         Age_at_sampling Residual
## StdDev:       0.1695767 11.28844
##
## Variance function:
##  Structure: Different standard deviations per stratum
##  Formula: ~1 | DiseaseStatus
##  Parameter estimates:
## Non-lesional     Lesional
##      1.00000      1.27481
## Fixed effects:  SBS1.5 ~ Age_at_sampling + MetaLocation + Disease_duration
##                        Value Std.Error  DF   t-value p-value
## (Intercept)         3.886816  4.716879 902  0.824023  0.4101
## Age_at_sampling     0.651671  0.081629 100  7.983308  0.0000
## MetaLocationArm   -11.645951  4.683731  91 -2.486469  0.0147
## MetaLocationBack   -6.402939  4.468874  91 -1.432786  0.1553
## MetaLocationFlank  -4.881964  4.462627  91 -1.093967  0.2769
## MetaLocationLeg    -8.267194  4.522802  91 -1.827892  0.0708
## Disease_duration    0.163104  0.063425  91  2.571609  0.0117
##  Correlation:
##                   (Intr) Ag_t_s MtLctA MtLctB MtLctF MtLctL
## Age_at_sampling   -0.571
## MetaLocationArm   -0.647 -0.027
## MetaLocationBack  -0.646 -0.076  0.703
## MetaLocationFlank -0.675 -0.040  0.714  0.755
## MetaLocationLeg   -0.649 -0.066  0.694  0.737  0.732
## Disease_duration  -0.011 -0.190 -0.001 -0.035  0.020  0.004
##
## Standardized Within-Group Residuals:
##        Min         Q1        Med         Q3        Max
## -5.8606464 -0.4886593 -0.1038414  0.4213027  4.2828992
##
## Number of Observations: 1100
## Number of Groups:
##               PatientID BiopsyID %in% PatientID
##                     102                     198
```

```
anova(model_clock.null,model_clock.dur, test=T)$"p-value"[2]
```

```
## [1] 0.01170614
```

```
clock.ints <- intervals(model_clock.dur, which="fixed")$fixed
clock.ints
```

```
##                          lower        est.      upper
## (Intercept)        -5.34101647   3.8868160 13.1146484
## Age_at_sampling     0.49023717   0.6516711  0.8131050
## MetaLocationArm   -20.91995779 -11.6459507 -2.3719435
## MetaLocationBack  -15.25151883  -6.4029389  2.4456410
## MetaLocationFlank -13.71817347  -4.8819642  3.9542451
## MetaLocationLeg   -17.22255446  -8.2671942  0.6881660
## Disease_duration    0.03751981   0.1631041  0.2886884
## attr(,"label")
## [1] "Fixed effects:"
```

```
summary(model_clock.dur)$tTable[,"p-value"]
```

```
##       (Intercept)   Age_at_sampling   MetaLocationArm  MetaLocationBack
##      4.101444e-01      2.468629e-12      1.472594e-02      1.553456e-01
## MetaLocationFlank   MetaLocationLeg  Disease_duration
##      2.768570e-01      7.084266e-02      1.174378e-02
```

When restricting to the mutation burden attributed to SBS1/5, the disease duration effect is (borderline) significant.

Pruning the phylogenetic trees

Some of the mutation clusters consisted of groups of mutations with VAFs too low for the pigeonhole principle to be incontrovertible. The calculations above assume that in such cases, the mutations all derive from a single sub-clone. However, there is a risk that the mutation burden represents not the burden of a single clone but the sum of the mutation burden for a collection of clones with similar cell fractions across all microbiopsies. This would lead to an over-estimation of the mutation rate for terminal branches of the phylogenetic trees. We performed pruning of the phylogenetic trees, retaining only branches representing nested clusters if the sum of the VAFs was greater than 1. For branches that represent single clusters (with no nesting), we pruned branches with VAF<0.3.

Unsurprisingly, this lowers the estimation of the total mutation rate. This new value should be thought of as a conservative lower bound.

```
## This file can be found in the Mendeley repository accompanying the manuscript.
clone_burden_after_pruning <- read.table("/nfs/users/nfs_s/so11/phd/psoriasis/bsub_jupyter_lab/psoriasis/manuscri
pt_data_and_figures/Supplementary_material/clone_mutation_burden_maxVAF03.txt", h=T)

clone_burden <- clone_burden_after_pruning
clone_burden <- merge(clone_burden, microd_meta[,c("SampleID", "BiopsyID", "MetaLocation","PatientID", "DiseaseSt
atus")], by.x="HighCellFrac_sample", by.y="SampleID")
clone_burden <- merge(clone_burden, patient_meta[,c("Patient.ID", "Age_at_sampling", "Disease_duration","Sex")],
by.x="PatientID", by.y="Patient.ID")

clone_burden$Disease_duration[clone_burden$DiseaseStatus=="Non-lesional" & !is.na(clone_burden$Disease_duration)]
<- 0

clone_burden$noPUVA <- clone_burden$TotalSBS_adj - clone_burden$PUVA

model_noPUVA.null <- lme(fixed = noPUVA ~ Age_at_sampling  + MetaLocation,
                      random = list(PatientID = pdSymm(form = ~ Age_at_sampling - 1), BiopsyID = pdSymm(form = ~
Age_at_sampling - 1)),
                      weights = varIdent(form= ~ 1 | DiseaseStatus),
                      data = clone_burden[!is.na(clone_burden$Disease_duration),], method="ML")

model_noPUVA.dur <- lme(fixed = noPUVA ~ Age_at_sampling  + MetaLocation + Disease_duration,
                      random = list(PatientID = pdSymm(form = ~ Age_at_sampling - 1), BiopsyID = pdSymm(form = ~
Age_at_sampling - 1)),
                      weights = varIdent(form= ~ 1 | DiseaseStatus),
                      data = clone_burden[!is.na(clone_burden$Disease_duration),], method="ML")

summary(model_noPUVA.null)
```

```
## Linear mixed-effects model fit by maximum likelihood
##   Data: clone_burden[!is.na(clone_burden$Disease_duration), ] 
##        AIC      BIC    logLik
##   11993.71 12040.99 -5986.854
## 
## Random effects:
##  Formula: ~Age_at_sampling - 1 | PatientID
##         Age_at_sampling
## StdDev:        4.592135
## 
##  Formula: ~Age_at_sampling - 1 | BiopsyID %in% PatientID
##         Age_at_sampling Residual
## StdDev:        3.333375  238.434
## 
## Variance function:
##  Structure: Different standard deviations per stratum
##  Formula: ~1 | DiseaseStatus 
##  Parameter estimates:
## Non-lesional     Lesional 
##      1.00000      1.14779 
## Fixed effects:  noPUVA ~ Age_at_sampling + MetaLocation 
##                      Value Std.Error  DF   t-value p-value
## (Intercept)       -99.36292  92.37722 642 -1.075621  0.2825
## Age_at_sampling     9.59962   1.57251  99  6.104640  0.0000
## MetaLocationArm   137.78163  91.09434  89  1.512516  0.1339
## MetaLocationBack   19.33995  87.07279  89  0.222112  0.8247
## MetaLocationFlank  -0.35792  87.28331  89 -0.004101  0.9967
## MetaLocationLeg    18.40436  89.15607  89  0.206429  0.8369
##  Correlation: 
##                   (Intr) Ag_t_s MtLctA MtLctB MtLctF
## Age_at_sampling   -0.589                            
## MetaLocationArm   -0.648 -0.023                     
## MetaLocationBack  -0.641 -0.087  0.705              
## MetaLocationFlank -0.672 -0.032  0.709  0.751       
## MetaLocationLeg   -0.633 -0.073  0.687  0.729  0.718
## 
## Standardized Within-Group Residuals:
##         Min          Q1         Med          Q3         Max 
## -4.86300912 -0.32063767 -0.08139216  0.23407789  7.66261854 
## 
## Number of Observations: 836
## Number of Groups: 
##               PatientID BiopsyID %in% PatientID 
##                     101                     194
```

```
noPUVA.ints <- intervals(model_noPUVA.null, which="fixed")$fixed
noPUVA.ints
```

```
##                        lower        est.     upper
## (Intercept)       -280.108801 -99.3629158  81.38297
## Age_at_sampling      6.490635   9.5996244  12.70861
## MetaLocationArm    -42.570176 137.7816330 318.13344
## MetaLocationBack  -153.049864  19.3399461 191.72976
## MetaLocationFlank -173.164513  -0.3579178 172.44868
## MetaLocationLeg   -158.109994  18.4043631 194.91872
## attr(,"label")
## [1] "Fixed effects:"
```

We should look at what effect the pruning of the trees has on the disease duration estimate. We see that the disease duration effect is much diminished and is no longer significant.

```
model_clock.null <- lme(fixed = SBS1.5 ~ Age_at_sampling  + MetaLocation,
                      random = list(PatientID = pdSymm(form = ~ Age_at_sampling - 1), BiopsyID = pdSymm(form = ~
Age_at_sampling - 1)),
                      weights = varIdent(form= ~ 1 | DiseaseStatus),
                      data = clone_burden[!is.na(clone_burden$Disease_duration),], method="ML")

model_clock.dur <- lme(fixed = SBS1.5 ~ Age_at_sampling  + MetaLocation + Disease_duration,
                      random = list(PatientID = pdSymm(form = ~ Age_at_sampling - 1), BiopsyID = pdSymm(form = ~
Age_at_sampling - 1)),
                      weights = varIdent(form= ~ 1 | DiseaseStatus),
                      data = clone_burden[!is.na(clone_burden$Disease_duration),], method="ML")

summary(model_clock.null)
```

```
## Linear mixed-effects model fit by maximum likelihood
##   Data: clone_burden[!is.na(clone_burden$Disease_duration), ]
##        AIC      BIC    logLik
##   6939.179 6986.465 -3459.589
##
## Random effects:
##  Formula: ~Age_at_sampling - 1 | PatientID
##         Age_at_sampling
## StdDev:       0.2075022
##
##  Formula: ~Age_at_sampling - 1 | BiopsyID %in% PatientID
##         Age_at_sampling Residual
## StdDev:       0.1145642 12.58384
##
## Variance function:
##  Structure: Different standard deviations per stratum
##  Formula: ~1 | DiseaseStatus
##  Parameter estimates:
## Non-lesional     Lesional
##     1.000000     1.079586
## Fixed effects:  SBS1.5 ~ Age_at_sampling + MetaLocation
##                         Value Std.Error  DF   t-value p-value
## (Intercept)          4.199626  4.148844 642  1.012240  0.3118
## Age_at_sampling      0.526600  0.069892  99  7.534455  0.0000
## MetaLocationArm     -8.566169  4.085368  89 -2.096792  0.0388
## MetaLocationBack    -4.841632  3.889280  89 -1.244866  0.2164
## MetaLocationFlank   -2.593310  3.902734  89 -0.664485  0.5081
## MetaLocationLeg     -7.317099  3.994117  89 -1.831969  0.0703
##  Correlation:
##                   (Intr) Ag_t_s MtLctA MtLctB MtLctF
## Age_at_sampling   -0.593
## MetaLocationArm   -0.647 -0.021
## MetaLocationBack  -0.641 -0.086  0.705
## MetaLocationFlank -0.675 -0.025  0.708  0.755
## MetaLocationLeg   -0.630 -0.074  0.686  0.731  0.719
##
## Standardized Within-Group Residuals:
##        Min         Q1        Med         Q3        Max
## -3.8881963 -0.5015500 -0.1786659  0.4588029  5.2659198
##
## Number of Observations: 836
## Number of Groups:
##               PatientID BiopsyID %in% PatientID
##                     101                      194
```

```
summary(model_clock.dur)
```

```
## Linear mixed-effects model fit by maximum likelihood
##   Data: clone_burden[!is.na(clone_burden$Disease_duration), ] 
##        AIC     BIC    logLik
##   6939.315 6991.33 -3458.658
## 
## Random effects:
##  Formula: ~Age_at_sampling - 1 | PatientID
##         Age_at_sampling
## StdDev:       0.2078412
## 
##  Formula: ~Age_at_sampling - 1 | BiopsyID %in% PatientID
##         Age_at_sampling Residual
## StdDev:       0.1116281 12.57851
## 
## Variance function:
##  Structure: Different standard deviations per stratum
##  Formula: ~1 | DiseaseStatus 
##  Parameter estimates:
## Non-lesional     Lesional 
##     1.000000     1.080191 
## Fixed effects:  SBS1.5 ~ Age_at_sampling + MetaLocation + Disease_duration 
##                       Value Std.Error  DF   t-value p-value
## (Intercept)        4.109724  4.143632 642  0.991817  0.3217
## Age_at_sampling    0.507873  0.071171  99  7.135978  0.0000
## MetaLocationArm   -8.611526  4.080120  88 -2.110606  0.0376
## MetaLocationBack  -5.092668  3.887480  88 -1.310018  0.1936
## MetaLocationFlank -2.505326  3.897091  88 -0.642871  0.5220
## MetaLocationLeg   -7.239487  3.988878  88 -1.814918  0.0729
## Disease_duration   0.074812  0.054693  88  1.367868  0.1748
##  Correlation: 
##                   (Intr) Ag_t_s MtLctA MtLctB MtLctF MtLctL
## Age_at_sampling   -0.579                                   
## MetaLocationArm   -0.646 -0.018                            
## MetaLocationBack  -0.640 -0.075  0.705                     
## MetaLocationFlank -0.675 -0.028  0.708  0.754              
## MetaLocationLeg   -0.630 -0.075  0.686  0.730  0.719       
## Disease_duration  -0.013 -0.196 -0.011 -0.048  0.015  0.013
## 
## Standardized Within-Group Residuals:
##        Min         Q1        Med         Q3        Max 
## -3.9160477 -0.4896735 -0.1751509  0.4563460  5.2779813 
## 
## Number of Observations: 836
## Number of Groups: 
##               PatientID BiopsyID %in% PatientID 
##                     101                     194
```

```
anova(model_clock.null,model_clock.dur, test=T)$"p-value"[2]
```

```
## [1] 0.1721982
```

```
clock.ints <- intervals(model_clock.dur, which="fixed")$fixed
clock.ints
```

```
##                         lower        est.      upper
## (Intercept)        -3.99284822  4.10972401 12.2122962
## Age_at_sampling     0.36724705  0.50787275  0.6484984
## MetaLocationArm   -16.68588828 -8.61152580 -0.5371633
## MetaLocationBack  -12.78580585 -5.09266842  2.6004690
## MetaLocationFlank -10.21748447 -2.50532616  5.2068321
## MetaLocationLeg   -15.13328578 -7.23948654  0.6543127
## Disease_duration   -0.03342182  0.07481223  0.1830463
## attr(,"label")
## [1] "Fixed effects:"
```

```
summary(model_clock.dur)$tTable[,"p-value"]
```

```
##       (Intercept)   Age_at_sampling   MetaLocationArm  MetaLocationBack 
##      3.216605e-01      1.614682e-10      3.764355e-02      1.935991e-01 
## MetaLocationFlank   MetaLocationLeg  Disease_duration 
##      5.219795e-01      7.294338e-02      1.748365e-01
```

Supplementary Note 5:

An R-markdown notebook describing the analysis of the psoralen mutational signature.

CodeS3: Characterisation of Psoralen signature

Sigurgeir Ólafsson

6/22/2022

Introduction

This document describes the various analyses carried out to characterise a mutational signature we identify and attribute to psoralen exposure.

To enable detailed characterisation of the signature, we performed whole genome sequencing of 16 microbiopsies which showed clear evidence of the signature in the whole-exome data. The terms psoralen and PUVA exposure are sometimes used interchangibly throughout this document. Originally we attributed the signature to PUVA exposure and the names of some of the directories and early files reflect that. The “PUVA-signature” and the “Psoralen-signature” are one and the same.

```
.libPaths("/lustre/scratch126/humgen/projects/psoriasis/R_packages_farm5_R4.1.0_install/")

library("TxDb.Hsapiens.UCSC.hg38.knownGene")
library(grid)
library(gridExtra)
library(MutationalPatterns)
ref_genome <- "BSgenome.Hsapiens.UCSC.hg38"
library(ref_genome, character.only = TRUE)
library(ggplot2)
library(reshape2)
library(ggsignif)
library(dplyr)
options(stringsAsFactors = F)

## Define a few variables
genes_hg38 <- genes(TxDb.Hsapiens.UCSC.hg38.knownGene)
sample_meta <- read.table("/nfs/users/nfs_s/so11/phd/psoriasis/bsub_jupyter_lab/psoriasis/sample_info/sample_meta
_wgs.txt", h=T, stringsAsFactors = F)
binomial_dir="/lustre/scratch126/humgen/projects/psoriasis/binomial_filters/"
repl_timing_dir="/nfs/users/nfs_s/so11/phd/psoriasis/bsub_jupyter_lab/psoriasis/07_signature_extraction/replicati
on_timing/"
puva_dir="/lustre/scratch126/humgen/projects/psoriasis/signature_extraction/puva_characterization/"

## Read in the data
sample_names <- sample_meta$sampleID
patient_list <- sample_meta$patient_ID
vcf_files <- paste(puva_dir, sample_names, ".vcf", sep="")
grl <- read_vcfs_as_granges(vcf_files, sample_names, ref_genome, predefined_dbs_mbs=T)
```

Mutational profiles of psoralen exposed samples

The mutational spectra of skin samples are typically dominated by the C>T mutations which characterise COSMIC signatures SBS7a and SBS7b. We can see that the samples highlighted here show evidence of some UV-exposure but their mutation spectra are dominated by T>A, T>C and T>G mutations at ApT sites.

```
type_occurrences <- mut_type_occurrences(grl, ref_genome)
plot_spectrum(type_occurrences, CT = TRUE,
              indv_points = TRUE, legend = T, by=patient_list)
```

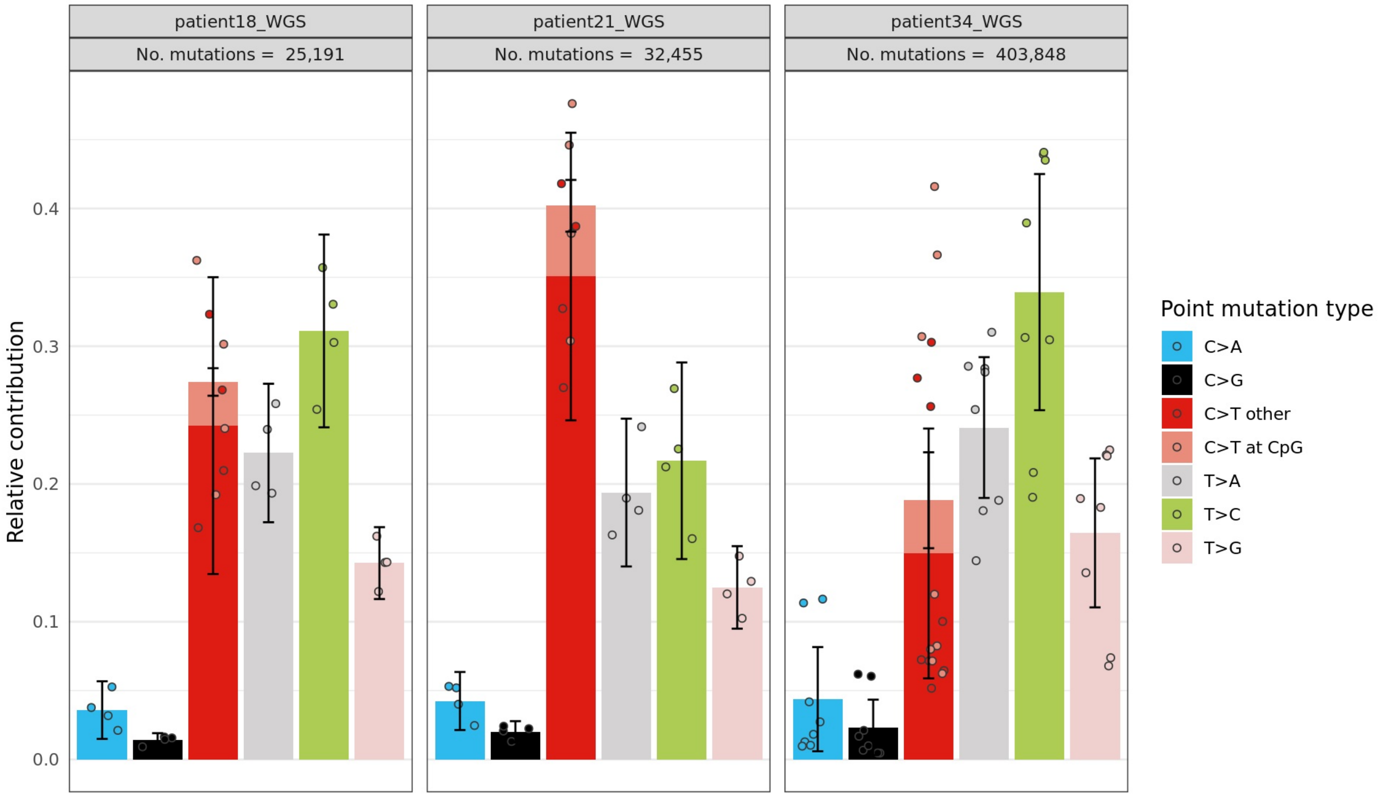


```
mut_mat <- mut_matrix(vcf_list = grl, ref_genome = ref_genome)
plot_96_profile(mut_mat[,c(1:4)])
```

```
## Warning: `guides(<scale> = FALSE)` is deprecated. Please use `guides(<scale> =
## "none")` instead.
```

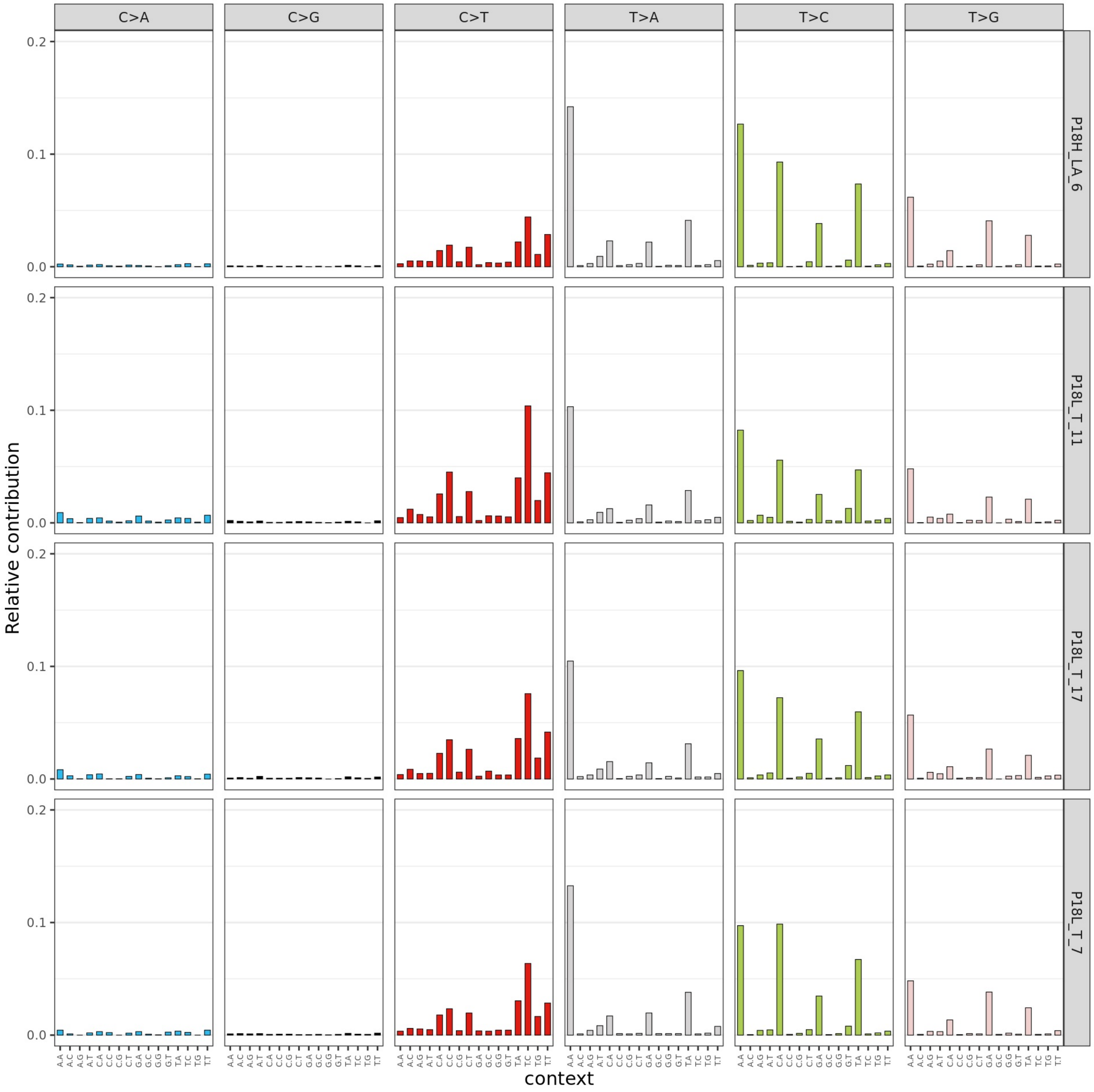


```
plot_96_profile(mut_mat[,c(5:8)])
```

```
## Warning: `guides(<scale> = FALSE)` is deprecated. Please use `guides(<scale> =
## "none")` instead.
```

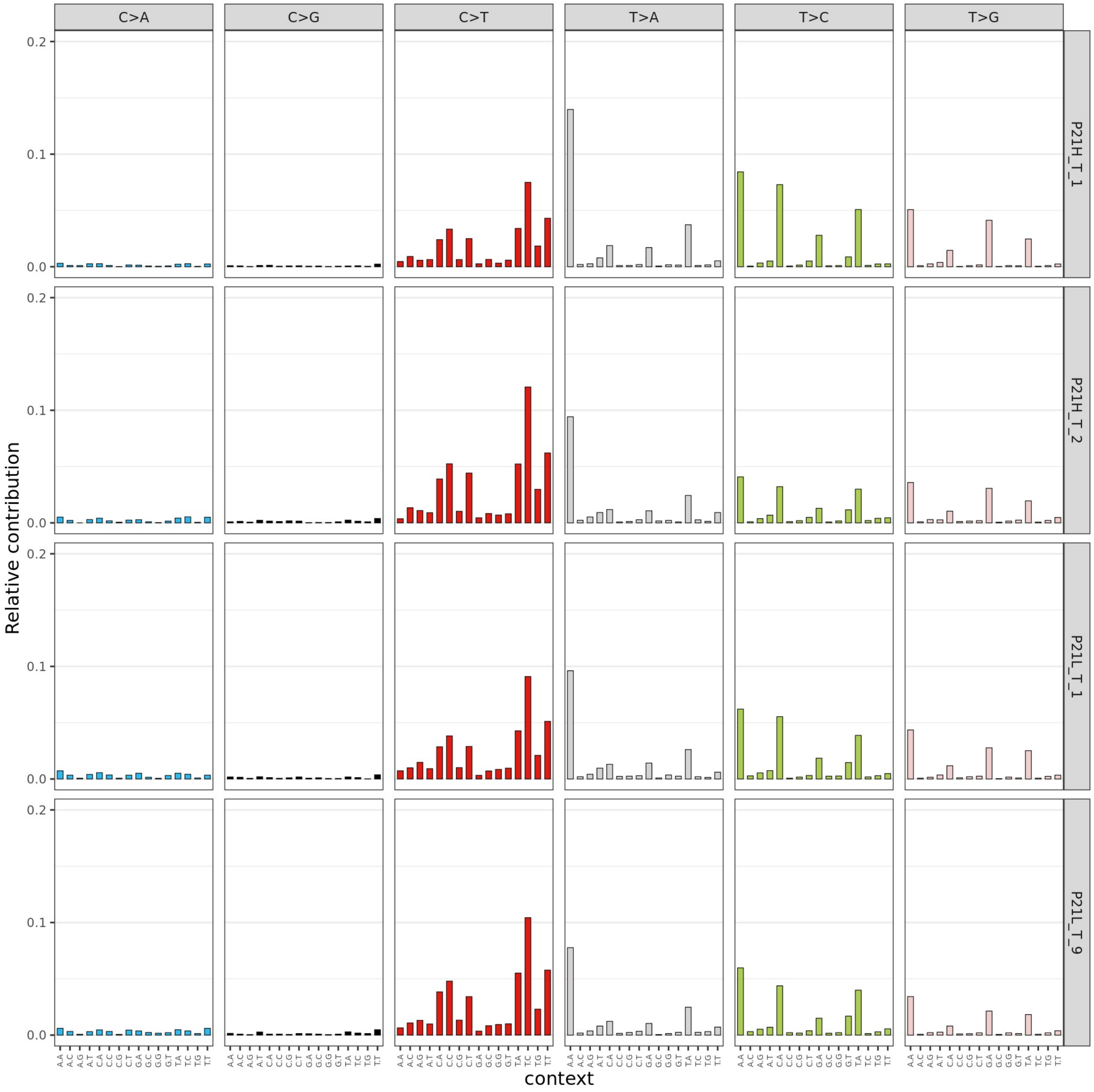


```
plot_96_profile(mut_mat[,c(9:16)])
```

```
## Warning: `guides(<scale> = FALSE)` is deprecated. Please use `guides(<scale> =
## "none")` instead.
```

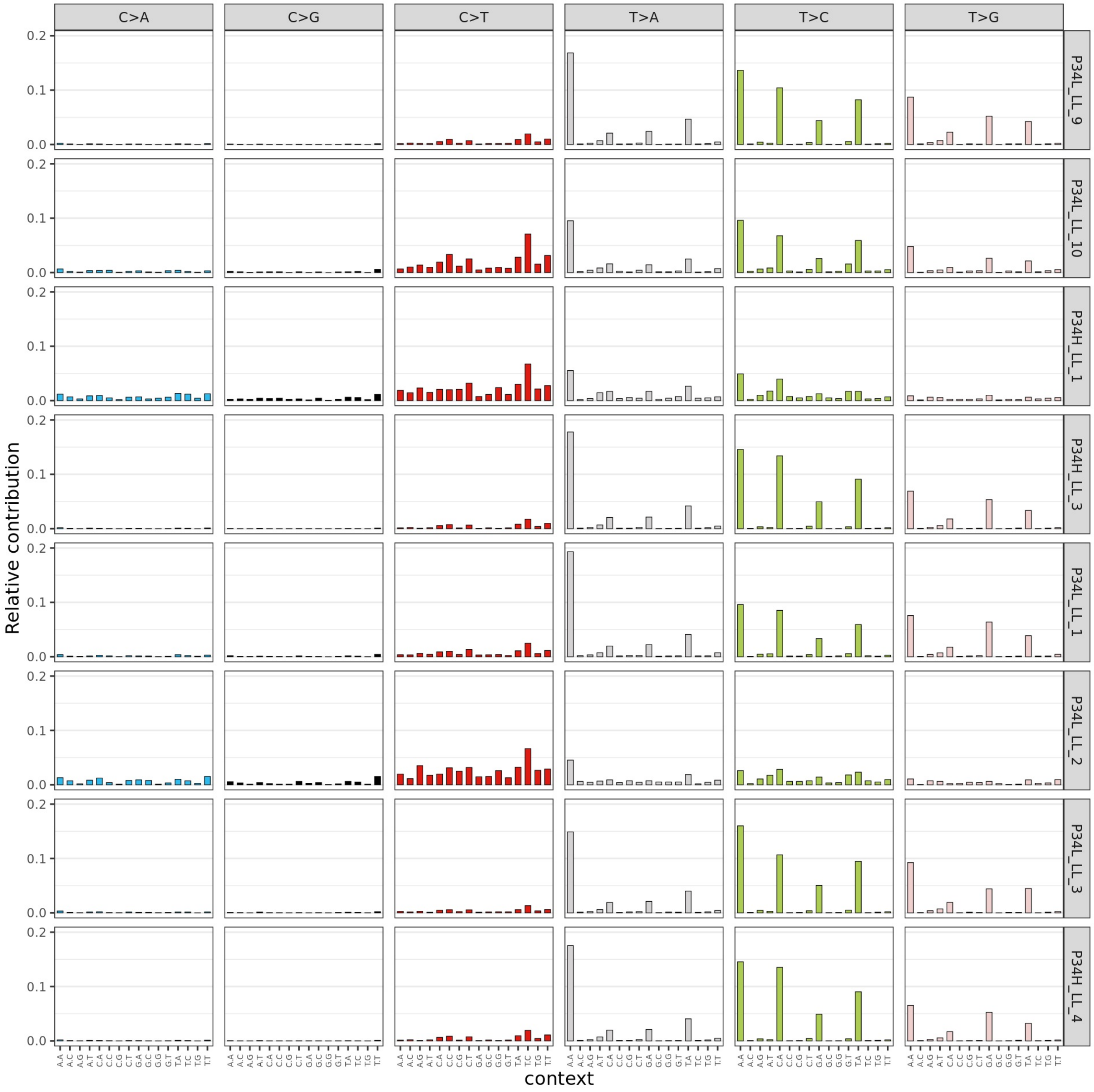

We may be interested in knowing if the trinucleotide model, which is classically used to describe mutational signatures, is sufficient to describe the context-dependence of this mutational process. We find that it is not so. The effect of sequence context extends beyond the trinucleotide model.

```
mut_mat_ext_context <- mut_matrix(grl, ref_genome, extension = 2)
plot_profile_heatmap(mut_mat_ext_context, by=patient_list)
```

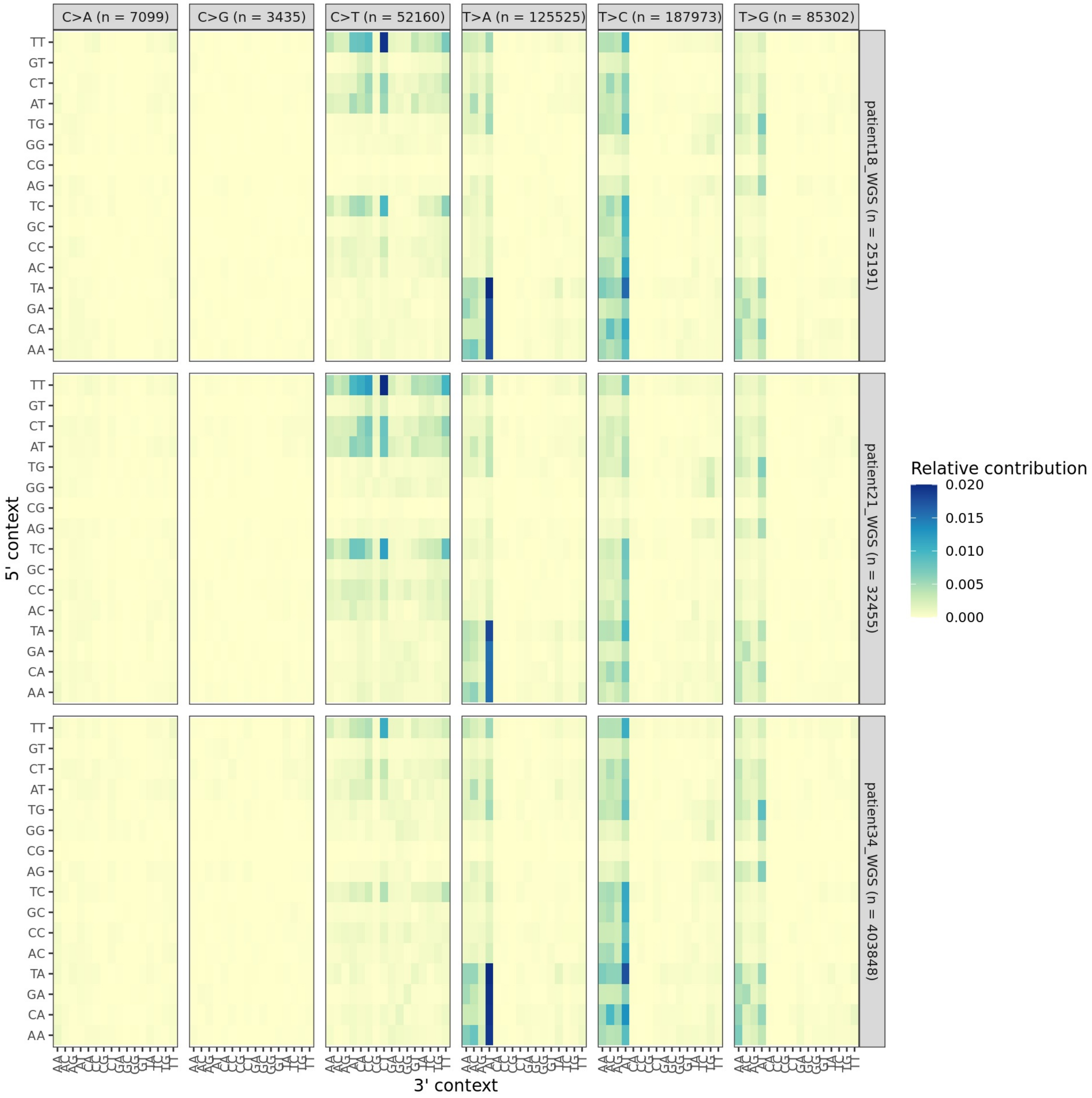

Indel spectra

We can compare the mutation spectra of indels from clones showing evidence of Psoralen exposure vs not. Because no non-exposed samples were whole-genome sequenced, this comparison will be done using the WES data.

I classify patients as psoralen-exposed if any clone from the patient has more than 100 psoralen-associated mutations. We then pool indels across patients in both types. This is because there are too few indels in individual clones (and even across individual patients) to do meaningful signature extraction.

```r
patients_wes <- read.table("/lustre/scratch126/humgen/projects/psoriasis/signature_extraction/hdp/snv/patient_lis
t.txt")
patients_wes <- patients_wes$V1[patients_wes$V1!="patient25"] ## Removed as no microbiopsies from lesional skin p
assed qc.

indels <- data.frame()
for(pat in patients_wes) {
  pat_indels <- read.table(paste(binomial_dir, pat, "/", pat, "_genotype_indels.txt", sep=""), h=T)
  pat_indels$patientID <- pat
  indels <- rbind(indels, data.frame(rownames(pat_indels), pat_indels$patientID))
}

colnames(indels) <- c("mutationID", "patientID")

indels$Chr <- unlist(strsplit(indels$mutationID, split=":"))[c(T,F,F,F)]
indels$Pos <- unlist(strsplit(indels$mutationID, split=":"))[c(F,T,F,F)]
indels$Ref <- unlist(strsplit(indels$mutationID, split=":"))[c(F,F,T,F)]
indels$Alt <- unlist(strsplit(indels$mutationID, split=":"))[c(F,F,F,T)]
indels$Pos <- as.numeric(indels$Pos)
indels$end.pos <- indels$Pos
indels$end.pos[nchar(indels$Ref)>nchar(indels$Alt)] <- indels$Pos[nchar(indels$Ref)>nchar(indels$Alt)]+nchar(inde
ls$Ref[nchar(indels$Ref)>nchar(indels$Alt)])-1

context_list <- indels  %>%
  dplyr::mutate(start = Pos - 3,
                end = Pos + 3) %>%
  dplyr::select(Chr, start,end)
this_range <- GenomicRanges::makeGRangesFromDataFrame(context_list)

this_seq <- getSeq(BSgenome.Hsapiens.UCSC.hg38, this_range)
out_seqs <- GenomicRanges::as.data.frame(this_seq)
out.table <- cbind(indels, out_seqs)

Grange_branches <- makeGRangesListFromDataFrame(indels, split.field = "patientID", keep.extra.columns = T, ignore
.strand = T, seqnames.field = "Chr",
                                                start.field = "Pos", end.field = "end.pos")
GenomeInfoDb::genome(Grange_branches) = 'hg38'

indel_grl <- get_indel_context(Grange_branches, ref_genome)
indel_counts <- count_indel_contexts(indel_grl)
indel_counts <- data.frame(indel_counts)

patient_meta <- read.table("/nfs/users/nfs_s/so11/phd/psoriasis/bsub_jupyter_lab/psoriasis/manuscript_data_and_fi
gures/Supplementary_Table1_final.txt", h=T)

exp <- indel_counts[,colnames(indel_counts) %in% patient_meta$Patient.ID[patient_meta$Shows_Psoralen_signature]]
nexp <- indel_counts[,!(colnames(indel_counts) %in% patient_meta$Patient.ID[patient_meta$Shows_Psoralen_signature
])]

sums <- data.frame(Exposed=rowSums(exp), NonExposed=rowSums(nexp))

plot_indel_contexts(sums, condensed = TRUE) +
  theme_classic() +
  theme(legend.position = "bottom") + labs(fill="") + theme(legend.key.size = unit(0.25, "cm")) +
  theme(legend.box.spacing = unit(-10, "pt")) + scale_y_continuous(expand=c(0,0))
```

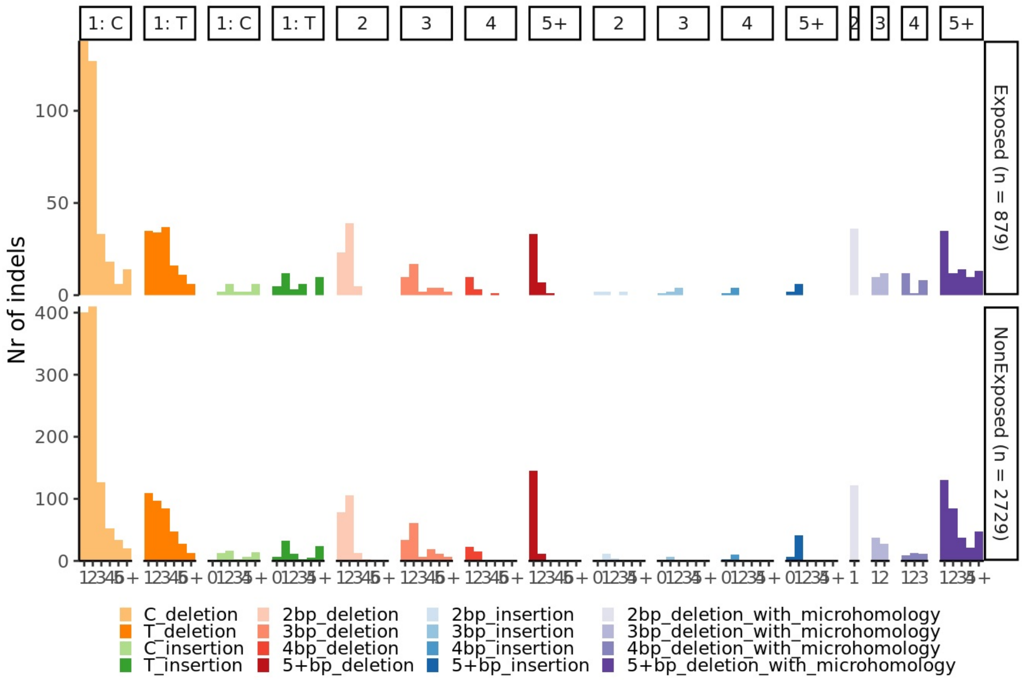

We see that the spectra are near-identical. For a formal comparison, we can calculate the cosine similarity of the probability vectors:

```
cos_sim(sums$Exposed, sums$NonExposed)
```

```
## [1] 0.9879375
```

Strand bias analyses

Transcriptional strand bias

We can start by plotting the mutation spectra of the samples as before but now with strand information. From the plot, it is obvious that there are many more T>[ACG] mutations on the untranscribed strand. This effect is much more pronounced than the known strand bias of UV-light exposure (C>T mutations below).

```
genes_hg38 <- genes(TxDb.Hsapiens.UCSC.hg38.knownGene)
mut_mat_s <- mut_matrix_stranded(grl, ref_genome, genes_hg38)
plot_192_profile(mut_mat_s[,1:4]) + theme_classic(base_size = 14) + theme(legend.position="top", axis.text.x = el
ement_text(angle=90, size=8))
```

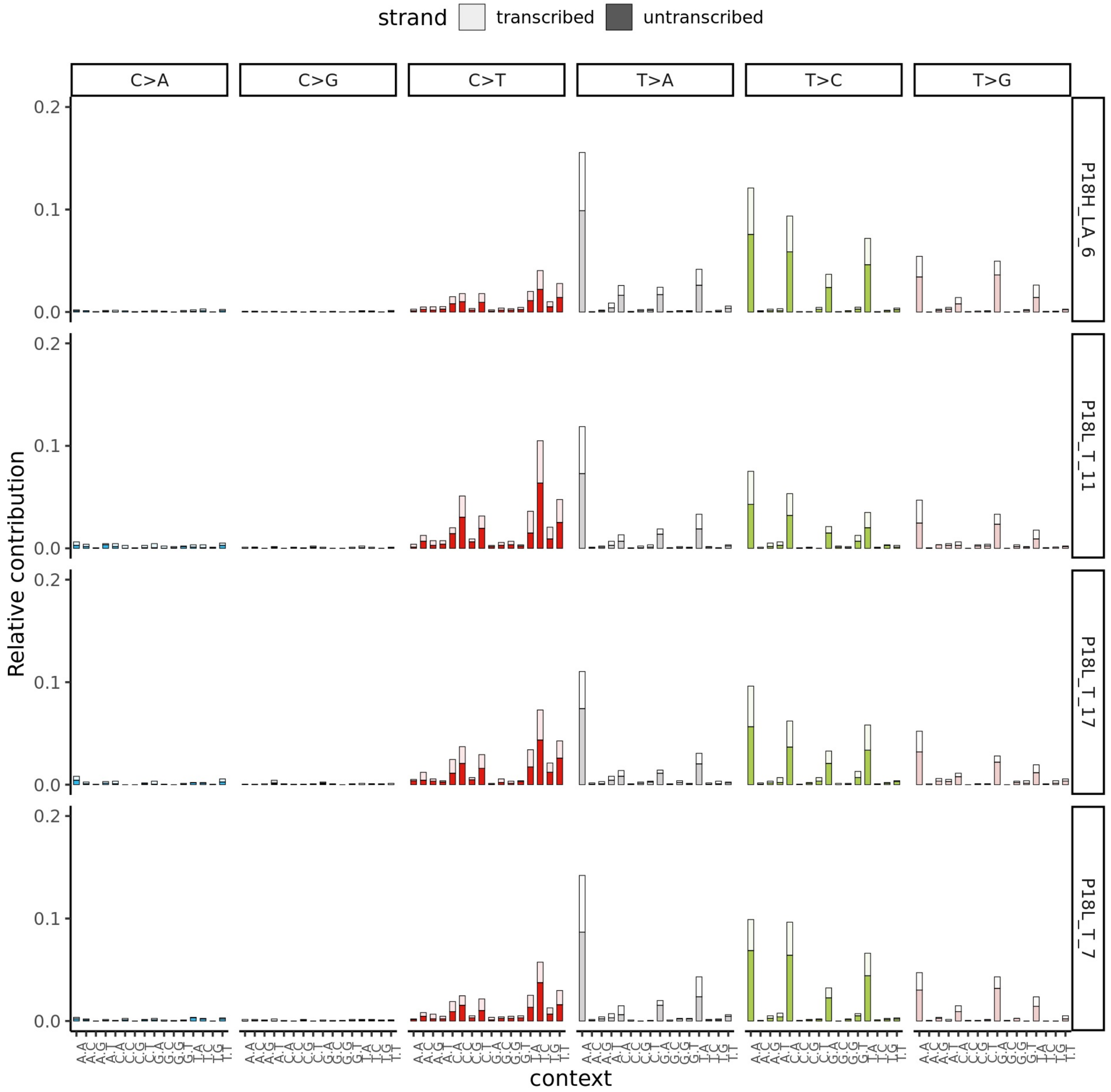


```
plot_192_profile(mut_mat_s[,5:8]) + theme_classic(base_size = 14) + theme(legend.position="top", axis.text.x = el
ement_text(angle=90, size=8))
```

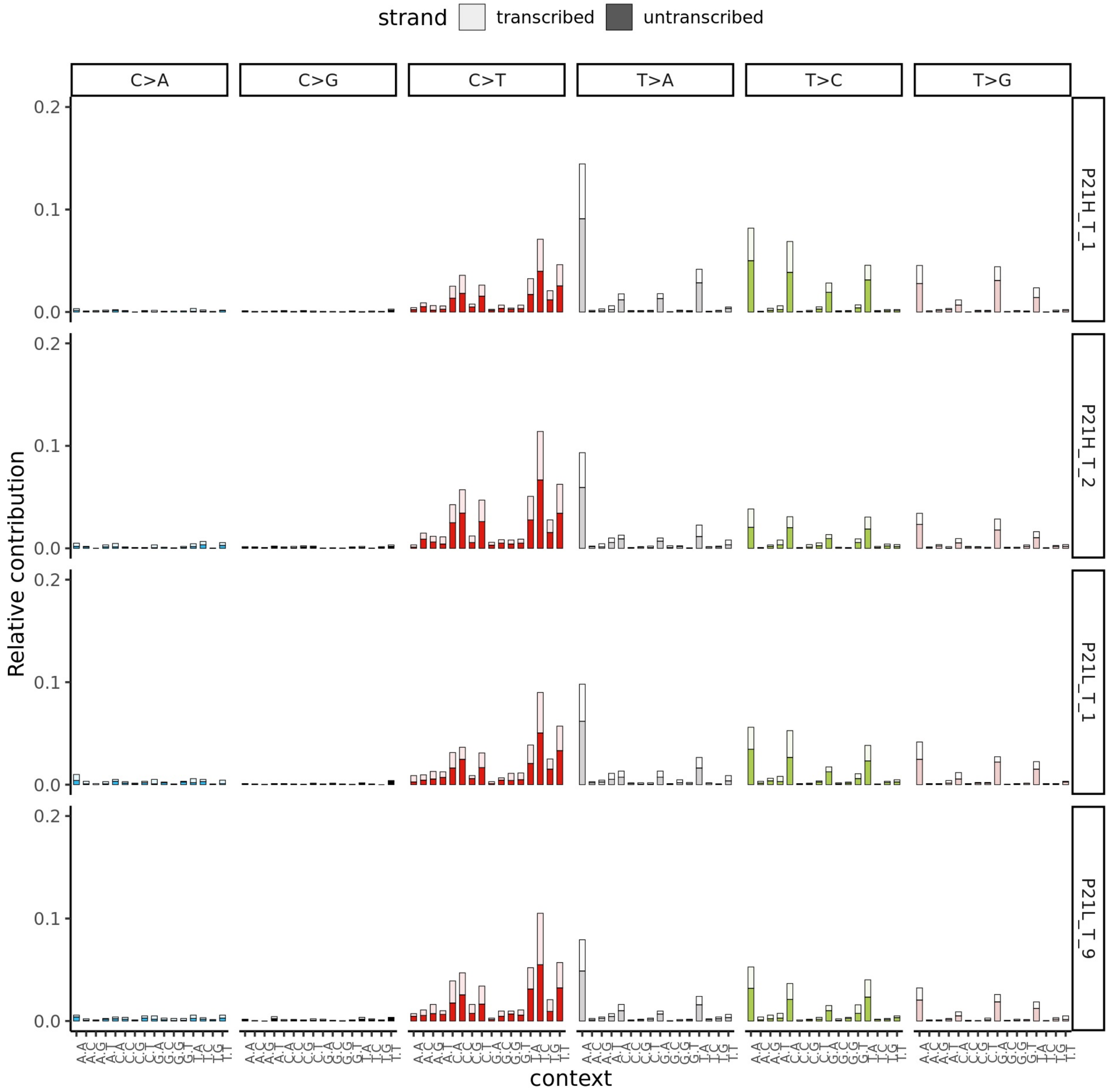


```
plot_192_profile(mut_mat_s[,9:16]) + theme_classic(base_size = 14) + theme(legend.position="top", axis.text.x = e
lement_text(angle=90, size=8))
```

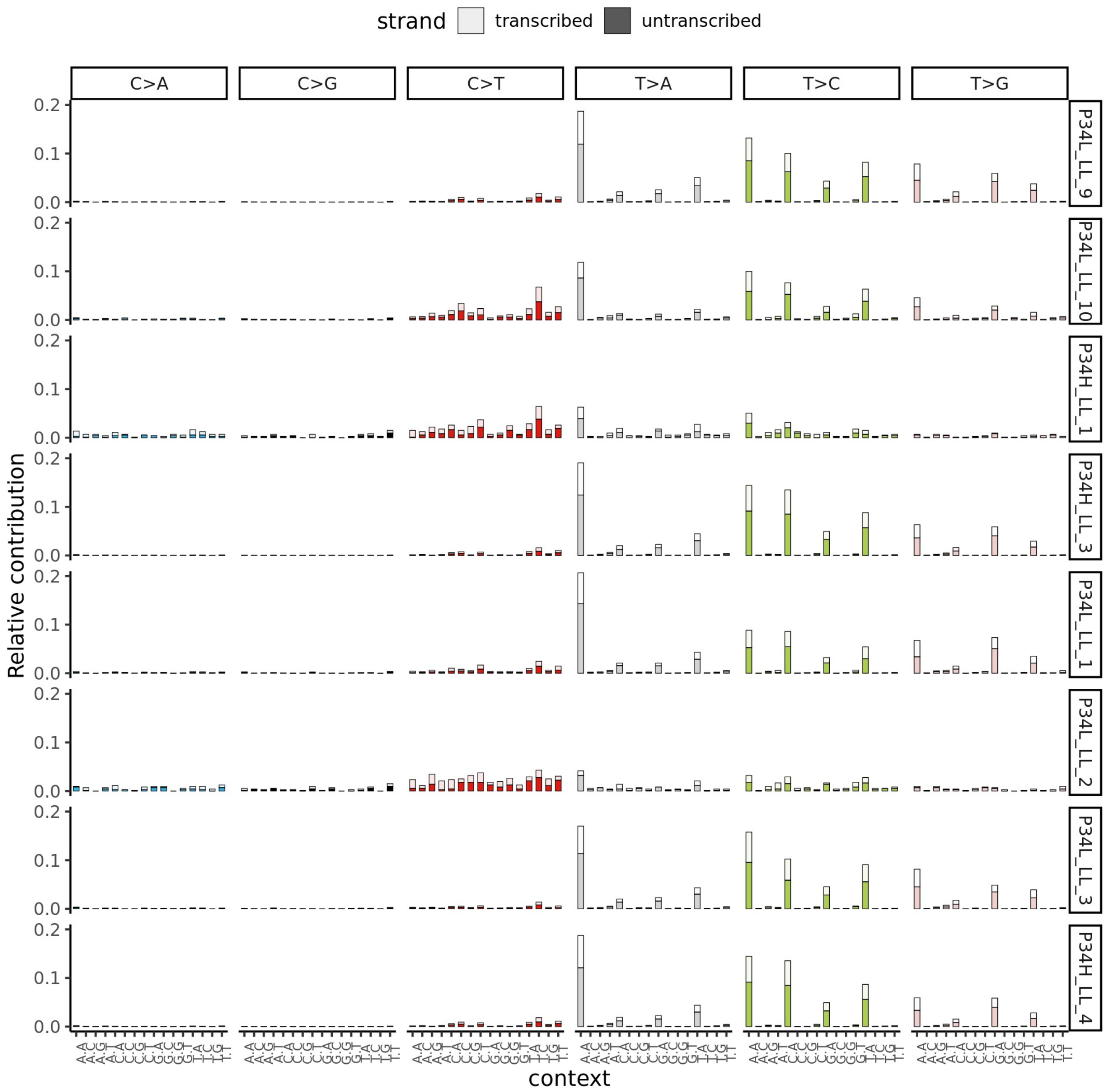

More formally, the strand_bias_test() function performs a two-sided Poisson test for the ratio between the number of mutation mapped on each strand. The difference is highly significant!

```
strand_counts <- strand_occurrences(mut_mat_s)
strand_bias <- strand_bias_test(strand_counts)
strand_bias
```

```
## # A tibble: 6 × 10
##   group type  transcribed untranscribed total ratio   p_poisson significant
##   <fct> <chr>       <dbl>         <dbl> <dbl> <dbl>       <dbl> <chr>
## 1 all   C>A          1680          1389  3069 1.21    1.62e-  7 *
## 2 all   C>G           725           804  1529 0.902   4.60e-  2 *
## 3 all   C>T         10478         12156 22634 0.862   7.01e- 29 *
## 4 all   T>A         20411         37874 58285 0.539 Inf.  e-324 *
## 5 all   T>C         30076         51325 81401 0.586 Inf.  e-324 *
## 6 all   T>G         14026         21998 36024 0.638 Inf.  e-324 *
## # … with 2 more variables: fdr <dbl>, significant_fdr <chr>
```

```
plot_strand(strand_counts, mode = "absolute")
```

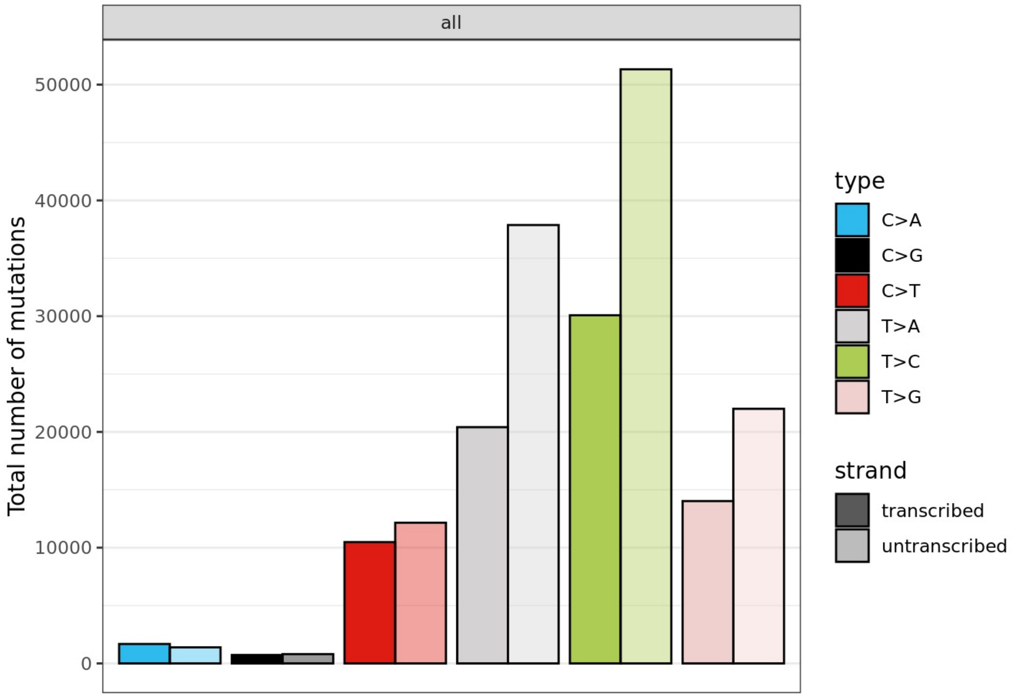


```
plot_strand_bias(strand_bias, sig_type = "p")
```

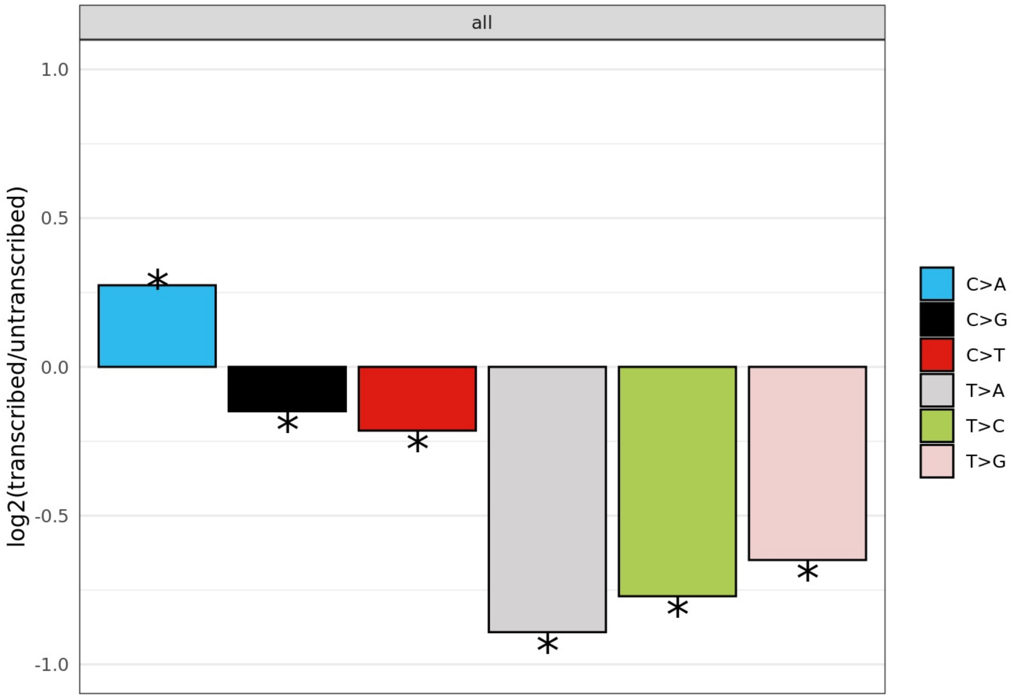

Psoralen and transcription

To assess the relationship between psoralen-related mutagenesis and gene expression, we used gene expression data from sun-exposed skin from the GTEx project (https://gtexportal.org/home/%5D). The transcription start site of each gene we got from Gencode.

```
## First read in GTEx results and merge with gencode
gtex <- read.csv("/lustre/scratch126/humgen/projects/psoriasis/resources/GTEx_skin_expression.csv", h=T)
colnames(gtex) <- c("TranscriptID", "Gene", "Skin_no_sun", "Skin_sun")

tss<-read.table("/lustre/scratch126/humgen/projects/psoriasis/resources/gencode.v27.annotation_chr_pos_strand_gen
eid_gene_name.gtf",h=F)
colnames(tss)<-c("Chromosome_phe","START_GENE","END_GENE","STRAND","Phenotype_ID","HGNC")
tss$TSS<-ifelse(tss$STRAND=="+",tss$START_GENE-1,tss$END_GENE-1)
```

We next extracted only protein coding genes from the nuclear genome and divided them up into 10 equally sized bins in order of ascending expression.

```
comb <- merge(gtex, tss, by.x="Gene", by.y="HGNC")
comb <- comb[order(comb$Skin_sun, decreasing = T),]
comb <- comb[comb$Chromosome_phe!="chrM",]
d <- duplicated(comb$Gene)
comb <- comb[!d,]  ## Keeps the highest expressed transcript

## Create expression bins to check the mutation rate in bins of ascending
## expression
bins <- comb[,c("Chromosome_phe", "START_GENE", "END_GENE", "Gene", "Skin_sun")]

sel_cv <- read.table("/nfs/users/nfs_s/so11/phd/psoriasis/bsub_jupyter_lab/psoriasis/manuscript_data_and_figures/
sel_cv_dNdS_results.txt", h=T)
bins <- bins[bins$Gene %in% sel_cv$gene_name,]  ## Only consider protein coding genes.

bins$nr <- 1:nrow(bins)
bins$expr_bin <- cut(bins$nr, breaks=10)
bins$expr_bin <- factor(bins$expr_bin, labels=c("Bin10","Bin9","Bin8","Bin7","Bin6","Bin5","Bin4","Bin3","Bin2","
Bin1"))
bins$nr <- NULL
#write.table(bins, file="/lustre/scratch126/humgen/projects/psoriasis/signature_extraction/puva_characterization/
GTex_expression_bins.bed", sep="\t", col.names = F, row.names = F, quote = F)
```

To calculate the mutation rate in each bin, we used the script Psoralen_expression.sh, which is available in the GitHub repository which accompanies this manuscript (see the main text). Here, I read in and plot the results.

```
expression_res <- read.table("/lustre/scratch126/humgen/projects/psoriasis/signature_extraction/puva_characteriza
tion/Mutation_rate_pr_expr_bin.txt", h=T)
expression_res$scaled <- expression_res$Mutation_Rate/expression_res$Mutation_Rate[1]

ggplot(expression_res, aes(x=Bin, y=scaled)) + geom_bar(position="dodge",stat="identity", fill="#E24E1B", colour=
"black") +
  scale_y_continuous(expand=c(0,0), limits = c(0,1.2)) + theme_bw(base_size = 14) +
  labs(y="Scaled mutation rate \n at TpA sites", x="Genes binned by increasing expression") +
  theme(axis.text.x = element_blank(), axis.ticks.x = element_blank()) +
  geom_hline(yintercept = 1, linetype=2)
```

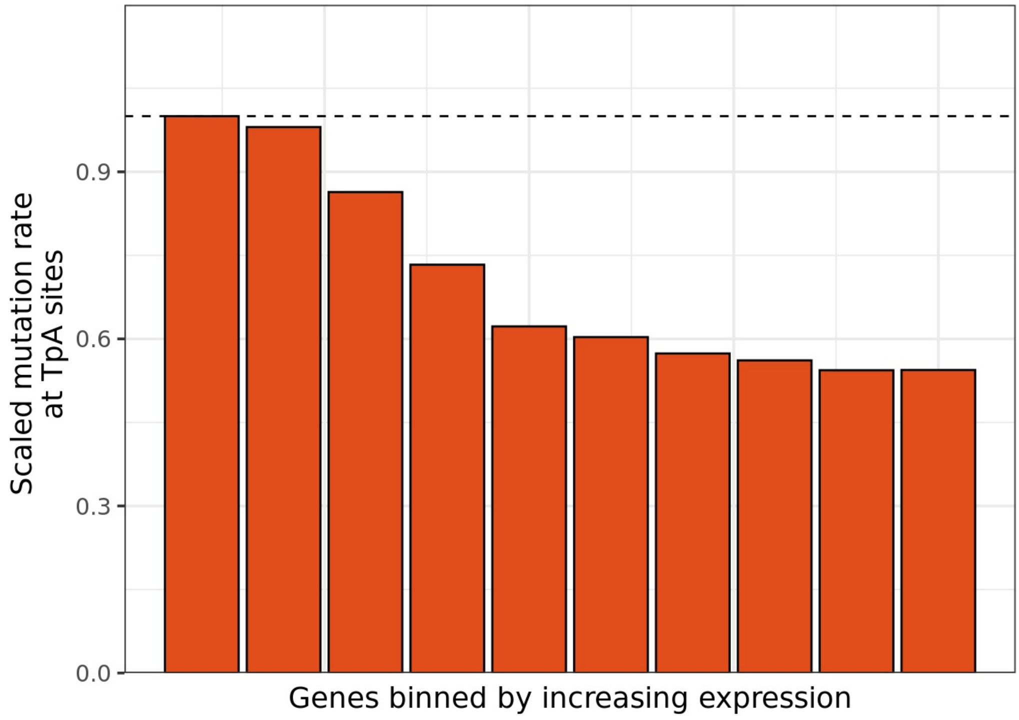

Transcription coupled repair and damage

To check for evidence of transcription coupled repair and damage, we'll continue using the GTEx data above. The procedure is as follows:

i. Compile a list of genes highly expressed in the skin. Include strand information. ii) Create 1kb bins 10kb up and downstream of the TSSs. iii) Count the number of T>A, T>C and T>G mutations at TpA sites on the transcribed strand and the number of A>T, A>G and A>C mutations at ApT sites on the untranscribed strand. If the gene is on the (+) strand, the transcribed strand is the complement of the reference strand.

```
## Create expression quintiles:
expr_quintile1 <-comb[comb$Gene %in% bins$Gene[bins$expr_bin=="Bin1" | bins$expr_bin=="Bin2"],]
expr_quintile2 <-comb[comb$Gene %in% bins$Gene[bins$expr_bin=="Bin3" | bins$expr_bin=="Bin4"],]
expr_quintile3 <-comb[comb$Gene %in% bins$Gene[bins$expr_bin=="Bin5" | bins$expr_bin=="Bin6"],]
expr_quintile4 <-comb[comb$Gene %in% bins$Gene[bins$expr_bin=="Bin7" | bins$expr_bin=="Bin8"],]
expr_quintile5 <-comb[comb$Gene %in% bins$Gene[bins$expr_bin=="Bin9" | bins$expr_bin=="Bin10"],]
```

```r
## Make a bed file for each quintile with all the information necessary for the analysis.
make_bed_file <- function(geneList) {

  toUse <- geneList
  ## For each of these genes, I want to write 20 entries to a bed file,
  ## ten 1kb segments upstream and downstream of the TSS.
  chr_vector <- as.character()
  start <- as.numeric()
  end <- as.numeric()
  tss <- as.character()
  strand <- as.character()
  label <- as.character()
  t <- 1
  for(i in 1:nrow(toUse)) {


    if(toUse$STRAND[i]=="-") {
      # Create 1kb bins upstream of the TSS
      for(j in 1:10) {
        chr_vector[t] <- toUse$Chromosome_phe[i]
        start[t] <- toUse$END_GENE[i] + (j-1)*1000
        end[t] <- toUse$END_GENE[i] + (j)*1000
        tss[t] <- "upstream"
        strand[t] <- "-"
        label[t] <- paste("-",j,"kb", sep="")
        t <- t+1
      }
      # Create 1kb bins downstream of the TSS
      for(k in 1:10) {
        chr_vector[t] <- toUse$Chromosome_phe[i]
        start[t] <- toUse$END_GENE[i] - (k)*1000
        end[t] <- toUse$END_GENE[i] - (k-1)*1000
        tss[t] <- "downstream"
        strand[t] <- "-"
        label[t] <- paste("+",k,"kb", sep="")
        t <- t+1
      }
    } else if(toUse$STRAND[i]=="+") {
      # Create 1kb bins upstream of the TSS
      for(j in 1:10) {
        chr_vector[t] <- toUse$Chromosome_phe[i]
        start[t] <- toUse$START_GENE[i] - (j)*1000
        end[t] <- toUse$START_GENE[i] - (j-1)*1000
        tss[t] <- "upstream"
        strand[t] <- "+"
        label[t] <- paste("-",j,"kb", sep="")
        t <- t+1
      }
      # Create 1kb bins downstream of the TSS
      for(k in 1:10) {
        chr_vector[t] <- toUse$Chromosome_phe[i]
        start[t] <- toUse$START_GENE[i] + (k-1)*1000
        end[t] <- toUse$START_GENE[i] + (k)*1000
        tss[t] <- "downstream"
        strand[t] <- "+"
        label[t] <- paste("+",k,"kb", sep="")
        t <- t+1
      }
    } else {
      stop("Strand info missing")
    }
  }

  bed_file <- data.frame(chr_vector, start, end, tss, strand, label)
  return(bed_file)
}

bed_q1 <- make_bed_file(expr_quintile1)
bed_q2 <- make_bed_file(expr_quintile2)
bed_q3 <- make_bed_file(expr_quintile3)
bed_q4 <- make_bed_file(expr_quintile4)
bed_q5 <- make_bed_file(expr_quintile5)

#write.table(bed_q5,file = "/lustre/scratch126/humgen/projects/psoriasis/signature_extraction/puva_characterizati
on/quintile5_expr_genes_skin.bed",  sep="\t", quote = F, row.names = F, col.names = F)
```

I next use the psoralen_tcd.sh script, available on the Github page accompanying the manuscript (see main text), to count the number of mutations in each bin.

We can then read in and plot the results:

```
for(q in c(1:5)) {
  assign(paste("quintile", q, "_Tmut_transcribed", sep=""),
         read.table(paste("/lustre/scratch126/humgen/projects/psoriasis/signature_extraction/puva_characterizatio
n/quintile", q, "_Tmut_transc.bed", sep="")))

  assign(paste("quintile", q, "_Amut_transcribed", sep=""),
         read.table(paste("/lustre/scratch126/humgen/projects/psoriasis/signature_extraction/puva_characterizatio
n/quintile", q, "_Amut_transc.bed", sep="")))

}

quintile1_Amut_transcribed$quintile=quintile1_Tmut_transcribed$quintile <- "Expression quintile1"
quintile2_Amut_transcribed$quintile=quintile2_Tmut_transcribed$quintile <- "Expression quintile2"
quintile3_Amut_transcribed$quintile=quintile3_Tmut_transcribed$quintile <- "Expression quintile3"
quintile4_Amut_transcribed$quintile=quintile4_Tmut_transcribed$quintile <- "Expression quintile4"
quintile5_Amut_transcribed$quintile=quintile5_Tmut_transcribed$quintile <- "Expression quintile5"

quintile1_Amut_transcribed$type=quintile2_Amut_transcribed$type=quintile3_Amut_transcribed$type=quintile4_Amut_tr
anscribed$type=quintile5_Amut_transcribed$type <- "A>[CGT] transcribed,\nT>[ACG] non-transcribed"
quintile1_Tmut_transcribed$type=quintile2_Tmut_transcribed$type=quintile3_Tmut_transcribed$type=quintile4_Tmut_tr
anscribed$type=quintile5_Tmut_transcribed$type <- "T>[ACG] transcribed,\nA>[CGT] non-transcribed"

comb <- rbind(quintile1_Amut_transcribed,quintile1_Tmut_transcribed,quintile2_Amut_transcribed,quintile2_Tmut_tra
nscribed,
              quintile3_Amut_transcribed,quintile3_Tmut_transcribed,quintile4_Amut_transcribed,
              quintile4_Tmut_transcribed,quintile5_Amut_transcribed,quintile5_Tmut_transcribed)

comb$V6 <- factor(comb$V6, levels=c("-10kb", "-9kb", "-8kb", "-7kb", "-6kb", "-5kb", "-4kb", "-3kb", "-2kb", "-1k
b",
                                    "+1kb", "+2kb", "+3kb", "+4kb", "+5kb", "+6kb", "+7kb", "+8kb", "+9kb", "+10k
b"))

m <- melt(data.frame(table(comb$V6, comb$quintile, comb$type)))
```

```
## Using Var1, Var2, Var3 as id variables
```

```
m$variable <- NULL
colnames(m) <- c("DistanceFromTSS","expression", "MutationType", "MutationCount")

standardized <- as.numeric()
for(i in 1:nrow(m)) {
  denom <- m$MutationCount[m$DistanceFromTSS=="-10kb" & m$MutationType==m$MutationType[i] & m$expression==m$expre
ssion[i]]
  standardized[i] <- m$MutationCount[i]/denom
}
m$standardized <- standardized

m$class <- paste(m$expression, m$MutationType)

#write.table(m, "/nfs/users/nfs_s/so11/phd/psoriasis/bsub_jupyter_lab/psoriasis/manuscript_data_and_figures/puva_
tcd.txt", row.names = F, sep="\t")

ggplot(m, aes(x=DistanceFromTSS, y=standardized, colour=expression)) + geom_point() + theme_bw(base_size = 14) +
  geom_line(aes(x=DistanceFromTSS,y=standardized, color = expression, group = class, linetype=MutationType)) +
  labs(x="Distance from the TSS", y="Mutation rate relative to intergenic regions", colour="", linetype="") +
  scale_y_continuous(trans="log2") + scale_colour_manual(values=c("#F79256","#FBD1A2", "#7DCFB6","#00B2CA","#1D4E
89"))
```

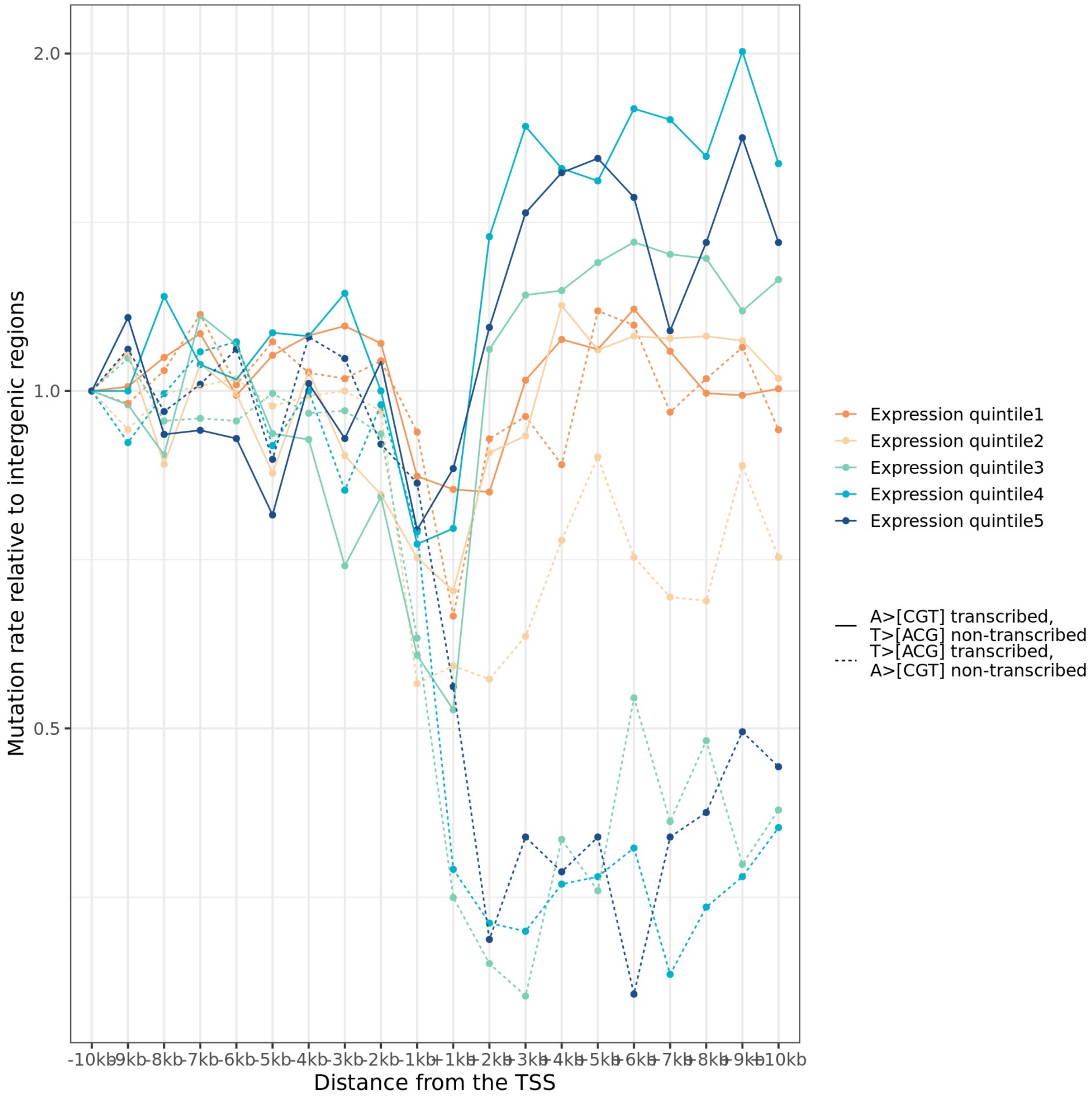

To test if the lines significantly depart from a straight line, we'll use a step function. We see that the effect of transcription coupled repair is highly significant from quintile 2 onwards and the effect of transcription coupled damage is highly significant from quintile 3 onwards.

```
counts <- rbind(data.frame(table(quintile1_Amut_transcribed$V6), quintile="Q1"),
                data.frame(table(quintile2_Amut_transcribed$V6), quintile="Q2"),
                data.frame(table(quintile3_Amut_transcribed$V6), quintile="Q3"),
                data.frame(table(quintile4_Amut_transcribed$V6), quintile="Q4"),
                data.frame(table(quintile5_Amut_transcribed$V6), quintile="Q5"))

counts$upstream_TSS <- 1
counts$upstream_TSS[grep("-", as.character(counts$Var1))] <- 0

P_vector_tcd <- as.numeric()
for(i in 1:5) {
  null_model <- lm(Freq ~ 1, data=counts[counts$quintile==paste("Q",i, sep=""),])
  alt_model <- lm(Freq ~ I(counts$upstream_TSS[counts$quintile==paste("Q",i, sep="")]), data=counts[counts$quinti
le==paste("Q",i, sep=""),])

  P_vector_tcd[i] <- anova(null_model, alt_model)$"Pr(>F)"[2]
}

counts <- rbind(data.frame(table(quintile1_Tmut_transcribed$V6), quintile="Q1"),
                data.frame(table(quintile2_Tmut_transcribed$V6), quintile="Q2"),
                data.frame(table(quintile3_Tmut_transcribed$V6), quintile="Q3"),
                data.frame(table(quintile4_Tmut_transcribed$V6), quintile="Q4"),
                data.frame(table(quintile5_Tmut_transcribed$V6), quintile="Q5"))

counts$upstream_TSS <- 1
counts$upstream_TSS[grep("-", as.character(counts$Var1))] <- 0

P_vector_tcr <- as.numeric()
for(i in 1:5) {
  null_model <- lm(Freq ~ 1, data=counts[counts$quintile==paste("Q",i, sep=""),])
  alt_model <- lm(Freq ~ I(counts$upstream_TSS[counts$quintile==paste("Q",i, sep="")]), data=counts[counts$quinti
le==paste("Q",i, sep=""),])

  P_vector_tcr[i] <- anova(null_model, alt_model)$"Pr(>F)"[2]
}
P_vector_tcr
```

```
## [1] 2.272361e-01 3.287565e-04 7.139100e-10 4.139985e-12 2.352863e-11
```

```
data.frame(quintile=paste("Q", c(1:5), sep=""), P_tcd=P_vector_tcd, P_tcr=P_vector_tcr)
```

```
##   quintile        P_tcd        P_tcr
## 1       Q1 0.4522413698 2.272361e-01
## 2       Q2 0.1555378104 3.287565e-04
## 3       Q3 0.0095397381 7.139100e-10
## 4       Q4 0.0002376581 4.139985e-12
## 5       Q5 0.0001896520 2.352863e-11
```

Replication strand bias

Next I want to see if there is evidence of replication strand bias. This is a bit trickier than the transcription strand bias, as the replication origins of the human genome are incompletely known. Nevertheless, there are some regions which are known to be conserved (see Methods).

Here I use a file containing replication time data from http://www.cell.com/abstract/S0092-8674(15)01714-6 (http://www.cell.com/abstract/S0092-8674(15)01714-6). In the file, left is synonymous to the leading strand because we are always using the reference as a point of reference. As shown below, there is an enrichment of Psoralen related mutations on the leading (left) strand.

```
repli_timing <- read.table("/nfs/users/nfs_s/so11/phd/psoriasis/bsub_jupyter_lab/psoriasis/07_signature_extractio
n/strand_asymmetries/replication_timing.repdirhg38.txt")
repli_timing$Class <- NA
repli_timing$Class[repli_timing$V5==1] <- "left"
repli_timing$Class[repli_timing$V6==1] <- "right"

repli_strand <- repli_timing[!is.na(repli_timing$Class), c("V1", "V2", "V3", "Class")]
repli_strand$Ratio <- 0
colnames(repli_strand)=c("Chr", "Start", "Stop", "Class", "Ratio")

# Store in GRanges object
repli_strand_granges <- GRanges(
  seqnames = repli_strand$Chr,
  ranges = IRanges(
    start = repli_strand$Start + 1,
    end = repli_strand$Stop
  ),
  strand_info = repli_strand$Class
)
# UCSC seqlevelsstyle
seqlevelsStyle(repli_strand_granges) <- "UCSC"
repli_strand_granges
```

```
## GRanges object with 10611 ranges and 1 metadata column:
##           seqnames            ranges strand | strand_info
##              <Rle>         <IRanges>  <Rle> | <character>
##       [1]     chr1   1164622-1264620      * |        left
##       [2]     chr1   1464622-1564620      * |       right
##       [3]     chr1   2368563-2468561      * |       right
##       [4]     chr1   2468563-2568561      * |       right
##       [5]     chr1   2568563-2668561      * |       right
##       ...      ...               ...    ... .         ...
##   [10607]    chr22 49406353-49506351      * |        left
##   [10608]    chr22 49506353-49606352      * |        left
##   [10609]    chr22 49606354-49706352      * |        left
##   [10610]    chr22 49706354-49806352      * |        left
##   [10611]    chr22 49806354-49906352      * |        left
##   -------
##   seqinfo: 23 sequences from an unspecified genome; no seqlengths
```

```
repli_strand_granges$strand_info <- factor(repli_strand_granges$strand_info,
                                           levels = c("right", "left")
)


mut_mat_s_rep <- mut_matrix_stranded(grl, ref_genome, repli_strand_granges,
                                     mode = "replication"
)
```

```
## Warning in .Seqinfo.mergexy(x, y): Each of the 2 combined objects has sequence levels not in the other:
##   - in 'x': chrX, chrY
##   - in 'y': chr7_KI270803v1_alt
##   Make sure to always combine/compare objects based on the same reference
##   genome (use suppressWarnings() to suppress this warning).
```

```
## Warning in mut_strand(gr, ranges, mode = mode): Some variants overlap with multiple genomic regions in the GRa
nges object.
##
##                These variants are assigned '-', as the strand cannot be determined.
##
##                To avoid this, make sure no genomic regions are overlapping in your GRanges object.
```

```
strand_counts_rep <- strand_occurrences(mut_mat_s_rep)
strand_bias_rep <- strand_bias_test(strand_counts_rep)
strand_bias_rep
```

```
## # A tibble: 6 × 10
##   group type   left right total ratio p_poisson significant      fdr
##   <fct> <chr> <dbl> <dbl> <dbl> <dbl>     <dbl> <chr>          <dbl>
## 1 all   C>A    1204  1294  2498 0.930  7.49e- 2 ""          1.12e- 1
## 2 all   C>G     600   598  1198 1.00   9.77e- 1 ""          9.78e- 1
## 3 all   C>T   10298 10293 20591 1.00   9.78e- 1 ""          9.78e- 1
## 4 all   T>A   26150 23232 49382 1.13   2.21e-39 "*"         1.32e-38
## 5 all   T>C   38220 35583 73803 1.07   2.90e-22 "*"         8.69e-22
## 6 all   T>G   17399 16338 33737 1.06   7.86e- 9 "*"         1.57e- 8
## # … with 1 more variable: significant_fdr <chr>
```

```
ps1 <- plot_strand(strand_counts_rep, mode = "relative")
ps2 <- plot_strand_bias(strand_bias_rep)
grid.arrange(ps1, ps2)
```

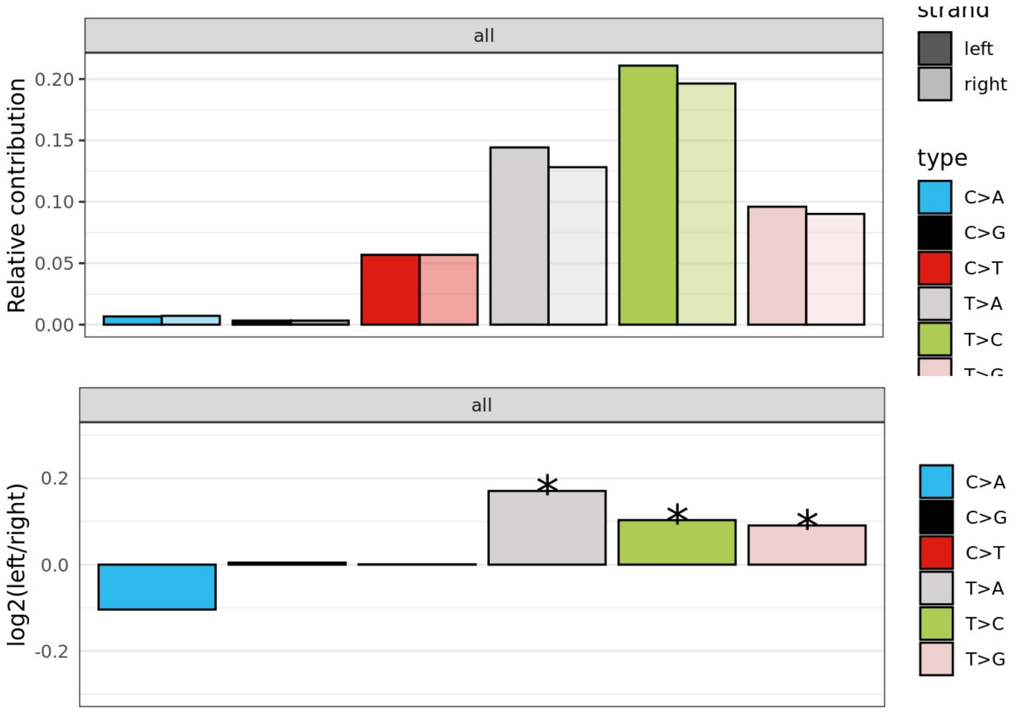

As an additional sanity check, we can use a different replication origin file, the one that comes with the MutationalPatterns package. This file is in hg19 so I first did a liftOver to convert the coordinates to hg38. We see broadly consistent results using this second source of replication origin information.

```
repli_strand <- read.table("/nfs/users/nfs_s/so11/phd/psoriasis/bsub_jupyter_lab/psoriasis/07_signature_extractio
n/strand_asymmetries/MutationalPatterns_repli_strandhg38.txt", h=T)


# Store in GRanges object
repli_strand_granges <- GRanges(
  seqnames = repli_strand$Chr,
  ranges = IRanges(
    start = repli_strand$Start + 1,
    end = repli_strand$Stop
  ),
  strand_info = repli_strand$Class
)
# UCSC seqlevelsstyle
seqlevelsStyle(repli_strand_granges) <- "UCSC"
repli_strand_granges
```

```
## GRanges object with 1936 ranges and 1 metadata column:
##          seqnames            ranges strand | strand_info
##             <Rle>         <IRanges>  <Rle> | <character>
##      [1]     chr1   3172437-3580436      * |        left
##      [2]     chr1   5162941-6367940      * |        left
##      [3]     chr1   6367941-7263940      * |       right
##      [4]     chr1   7263941-9551942      * |        left
##      [5]     chr1 11434944-12196943      * |        left
##      ...      ...               ...    ... .         ...
##   [1932]     chrY 21577115-21659114      * |       right
##   [1933]     chrY 21850854-22277853      * |       right
##   [1934]     chrY 22277854-26489853      * |        left
##   [1935]     chrY 26489854-26539853      * |       right
##   [1936]     chrY 26539854-26613853      * |        left
##   -------
##   seqinfo: 24 sequences from an unspecified genome; no seqlengths
```

```
repli_strand_granges$strand_info <- factor(repli_strand_granges$strand_info,
                                           levels = c("right", "left")
)


mut_mat_s_rep <- mut_matrix_stranded(grl, ref_genome, repli_strand_granges,
                                     mode = "replication"
)
strand_counts_rep <- strand_occurrences(mut_mat_s_rep)
strand_bias_rep <- strand_bias_test(strand_counts_rep)
strand_bias_rep
```

```
## # A tibble: 6 × 10
##   group type   left right total ratio p_poisson significant      fdr
##   <fct> <chr> <dbl> <dbl> <dbl> <dbl>     <dbl> <chr>          <dbl>
## 1 all   C>A    1247  1288  2535 0.968  4.27e- 1 ""          5.12e- 1
## 2 all   C>G     618   595  1213 1.04   5.28e- 1 ""          5.28e- 1
## 3 all   C>T   10901 10512 21413 1.04   8.01e- 3 "*"         1.20e- 2
## 4 all   T>A   26847 24938 51785 1.08   5.05e-17 "*"         3.03e-16
## 5 all   T>C   37389 35325 72714 1.06   1.99e-14 "*"         5.98e-14
## 6 all   T>G   16480 15860 32340 1.04   5.77e- 4 "*"         1.15e- 3
## # … with 1 more variable: significant_fdr <chr>
```

```
ps1 <- plot_strand(strand_counts_rep, mode = "relative")
ps2 <- plot_strand_bias(strand_bias_rep)
grid.arrange(ps1, ps2)
```

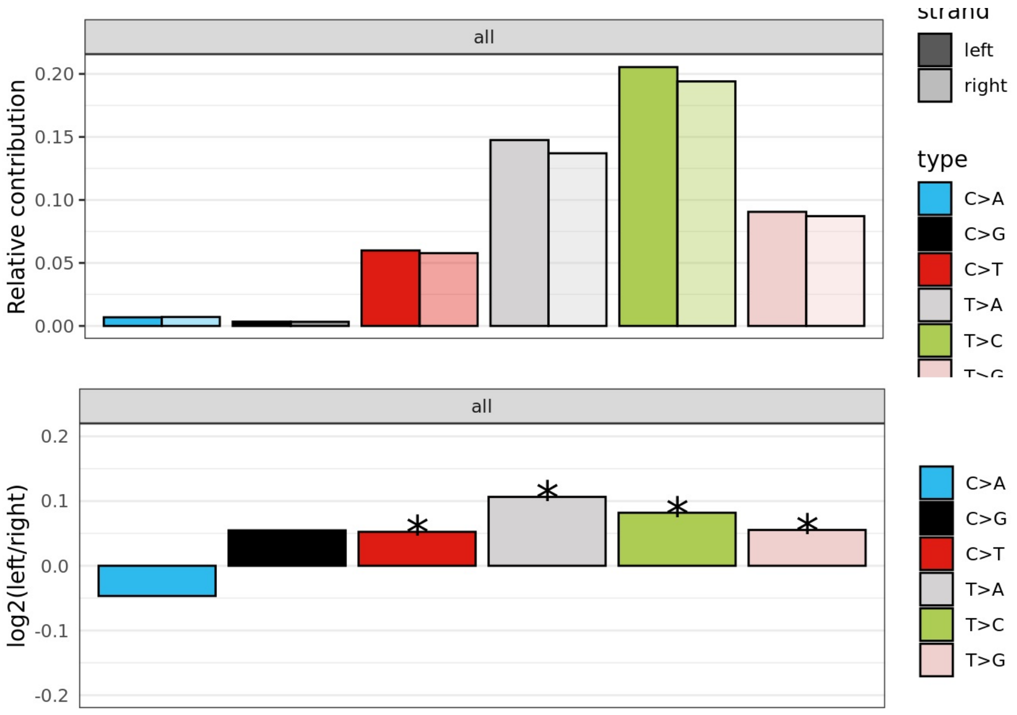

Replication timing analysis

To see if replication timing affects the mutation rate of psoralen exposure, we estimate the mutation rate after dividing the genome up into four bins of ascending replication timing. The code for doing this analysis is available in puva_replication_timing.sh in the github repository that accompanies this manuscript (see main text). Here I just read in and plot the results.

```
rt_results <- data.frame()
for(sample in sample_names) {
  rt <- read.table(paste(puva_dir, sample, "_RT_results.txt", sep=""))
  rt$sample <- sample
  rt_results <- rbind(rt_results, rt)
}

ggplot(rt_results, aes(x=V1, y=log10(V2))) +geom_point() +
  geom_line(aes(group=sample, colour=sample)) + theme_classic(base_size = 14) +
  labs(y="log10(Mutations per TA/AT site)", x="")
```

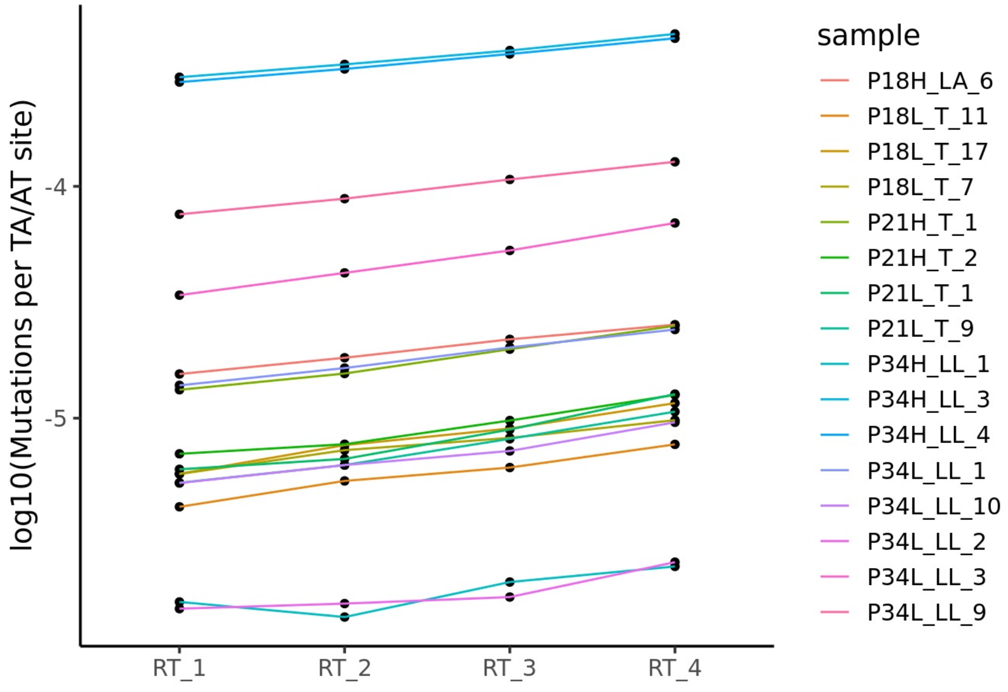

Effect of psoralen exposure on the clonal structure of the tissue

Psoralen exposure (and PUVA treatment in particular) results in interstrand cross links that are toxic to cells. It is possible that high exposure may result in differences in the clonal composition of the tissue and possibly different selection pressures compared with unexposed skin.

We test for a difference in clonality first on the level of individual microbiopsies.

```
puva_colours <- c("#FCBF49", "#40476D")
clone_sigs <- read.table("/nfs/users/nfs_s/so11/phd/psoriasis/bsub_jupyter_lab/psoriasis/manuscript_data_and_figu
res/Supplementary_material/Supplementary_Table3_clone_mutationBurden.txt", h=T)
microd_meta <- read.table("/nfs/users/nfs_s/so11/phd/psoriasis/bsub_jupyter_lab/psoriasis/manuscript_data_and_fig
ures/Supplementary_material/Supplementary_Table2_microdissection_metadata.txt", h=T)
x <- clone_sigs[clone_sigs$PUVA>100,]
puva_exp_patients <- unique(unlist(strsplit(x$CloneID, spli="_"))[c(T,F)])
microd_meta$PUVA_exposed <- F
microd_meta$PUVA_exposed[microd_meta$PatientID %in% puva_exp_patients] <- T

puva_vaf_test <- wilcox.test(microd_meta$MedianVAF~microd_meta$PUVA_exposed)
puva_vaf_test
```

```
##
##  Wilcoxon rank sum test with continuity correction
##
## data:  microd_meta$MedianVAF by microd_meta$PUVA_exposed
## W = 66641, p-value = 2.88e-09
## alternative hypothesis: true location shift is not equal to 0
```

```
ggplot(microd_meta, aes(x=PUVA_exposed, y=MedianVAF)) + geom_boxplot(aes(fill=PUVA_exposed)) +
  theme_classic(base_size = 14) + labs(x="", y="Median VAF of microbiopsy") +
  theme(legend.position = "none") +
  scale_x_discrete(labels=c("Not exposed", "Psoralen Exposed")) +
  scale_fill_manual(values=puva_colours) + scale_y_continuous(limits = c(0,0.6)) +
  geom_signif(annotation = paste("P = ", formatC(puva_vaf_test$p.value, digits=2), sep=""),
              y_position = c(0.55), xmin = c(1), xmax = c(2),
              tip_length = c(0.03, 0.03))
```

```
## Warning: Removed 8 rows containing non-finite values (stat_boxplot).
```

```
## Warning: Removed 8 rows containing non-finite values (stat_signif).
```

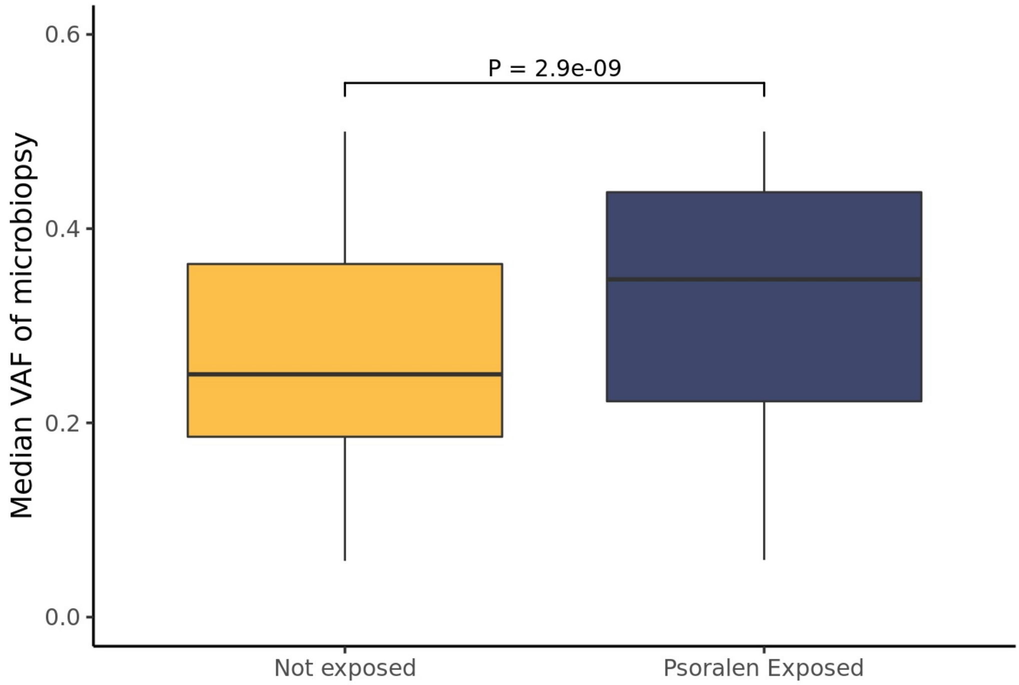

However, as pointed out by one of the reviewers, the patients who have received PUVA treatment are likely to be older than those who have not. As clones increase in size with age, it is important to take the age of the patient into account. We test if including a binary variable for the presence/absence of the psoralen signature significantly improves the fit of a linear model between mVAF and age.

```
microd_meta <- read.table("/nfs/users/nfs_s/so11/phd/psoriasis/bsub_jupyter_lab/psoriasis/manuscript_data_and_fig
ures/Microdissection_metaData.txt", h=T)

mVAFs <- data.frame(tapply(microd_meta$MedianVAF, microd_meta$PatientID, median, na.rm=T))
mVAFs$PatientID <- rownames(mVAFs)
colnames(mVAFs)[1] <- "MedianVAF"
patient_meta <- merge(patient_meta, mVAFs, by.x="Patient.ID", by.y="PatientID")
patient_meta$labels <- ifelse(patient_meta$Shows_Psoralen_signature, "Psoralen signature seen", "Signature not se
en")

puva_colours <- c("#FCBF49", "#40476D")

ggplot(patient_meta, aes(x=Age_at_sampling, y=MedianVAF, colour=labels)) + geom_point() +
  geom_smooth(method="lm") + theme_classic() +
  labs(y="Median VAF \n of microbiopsies", x="Patient Age", colour="") +
  theme(legend.position = "top") + scale_colour_manual(values=rev(puva_colours)) +
  guides(colour=guide_legend(nrow=2,byrow=TRUE))
```

```
## `geom_smooth()` using formula 'y ~ x'
```

```
## Warning: Removed 5 rows containing non-finite values (stat_smooth).
```

```
## Warning: Removed 5 rows containing missing values (geom_point).
```

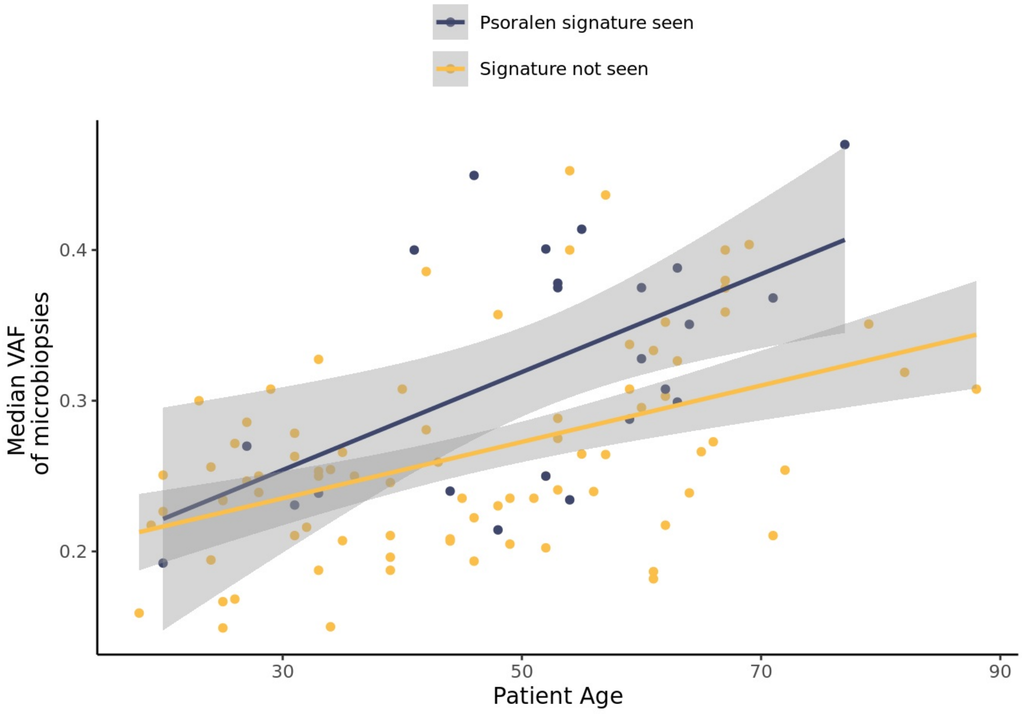


```
## Do the likelihood ratio test:
model_null <- lm(MedianVAF~Age_at_sampling, data=patient_meta)
model_null_psoralen <- lm(MedianVAF~Age_at_sampling + Shows_Psoralen_signature, data=patient_meta)
anova(model_null,model_null_psoralen)
```

```
## Analysis of Variance Table
##
## Model 1: MedianVAF ~ Age_at_sampling
## Model 2: MedianVAF ~ Age_at_sampling + Shows_Psoralen_signature
##   Res.Df    RSS Df Sum of Sq      F   Pr(>F)
## 1    104 0.4322
## 2    103 0.3928  1  0.039402 10.332 0.001746 **
## ---
## Signif. codes:  0 '***' 0.001 '**' 0.01 '*' 0.05 '.' 0.1 ' ' 1
```

Signature potential damage analysis

We would like to know if the psoralen signature is especially likely to cause any particular type of mutation in genes that are known drivers in squamous cell carcinomas or in normal skin.

Start by defining a list of entrez-gene IDs for the genes of interest.

```
## Genes mutated in SCCs and Normal skin:
# ARID2, ASXL1, CASP8, CDKN2A, FAT1, KMT2D, NOTCH1, NOTCH2, PPM1D, RB1, RBM10, TP53, TP63, AJUBA, NOTCH3
gene_ids <- c(196528,171023,841,1029,2195,8085,4851,4853,8493,5925,8241,7157,8626,84962,4854)
txdb <- TxDb.Hsapiens.UCSC.hg38.knownGene

all_sigs <- read.csv("/lustre/scratch126/humgen/projects/psoriasis/resources/sigProfiler_SBS_signatures_summing_t
o_one.csv", h=T,row.names = 1, stringsAsFactors = F)
psoralen_component <- read.table("/nfs/users/nfs_s/so11/phd/psoriasis/bsub_jupyter_lab/psoriasis/manuscript_data_
and_figures/Supplementary_material/Supplementary_Table5_Psoralen_signature_hdp_component.txt", h=T)
colnames(psoralen_component) <- "Psoralen"
signatures <- data.frame(cbind(psoralen_component, all_sigs))
```

The signature_potential_damage_analysis() function in MutationalPatterns gives an estimate of the enrichment of particular mutations relative to a uniform mutational process. However, the mutational spectrum of the skin is not uniform and I would also like to give this relative to SBS7b, which is the dominant mutational process in our study. Need to make minor changes to the function.

```
signature_potential_damage_analysis_sbs7b_ref <- function (signatures, contexts, context_mismatches)
{
  context <- ratio <- contribution <- sig <- flat <- n <- NULL
  ratio_by_background <- flat_ratio <- flat_blosum62 <- NULL
  blosum62 <- blosum62_min_background <- NULL
  signatures <- as.data.frame(signatures)
  signatures$context <- contexts
  nr_features <- nrow(signatures)
  signatures$flat_background <- rep(1/nr_features, nr_features)
  sig_context_mismatch <- signatures %>% dplyr::full_join(context_mismatches,
                                                          by = "context") %>% tidyr::pivot_longer(cols = c(-conte
xt,
                                                                                                              -type,
-ratio, -n, -blosum62), names_to = "sig", values_to = "contribution")
  sig_mismatch <- sig_context_mismatch %>% dplyr::mutate(ratio = contribution *
                                                           ratio, n = contribution * n, blosum62 = contribution *
                                                           blosum62) %>% dplyr::group_by(type, sig) %>% dplyr::su
mmarise(n = sum(n),

ratio = sum(ratio), blosum62 = sum(blosum62), .groups = "drop_last") %>%
    dplyr::ungroup()

  sbs7b_sig <- sig_mismatch %>% dplyr::filter(sig == "SBS7b") %>%
    dplyr::select(type, flat_ratio = ratio, flat_blosum62 = blosum62)

  norm_sig_mismatch <- sig_mismatch %>% dplyr::full_join(sbs7b_sig,
                                                         by = "type") %>% dplyr::mutate(ratio_by_background = rat
io/flat_ratio,
                                                                                        blosum62_min_background =
blosum62 - flat_blosum62) %>%
    dplyr::select(type, sig, ratio, ratio_by_background,
                  n, blosum62, blosum62_min_background) %>% dplyr::filter(sig !=
                                                                            "flat_background") %>% dplyr::arrange
(sig)
  return(norm_sig_mismatch)
}
```

Now we can look at the major signatures in the skin and plot the results.

```
contexts <- rownames(mut_mat)
context_mismatches <- context_potential_damage_analysis(contexts, txdb, ref_genome, gene_ids)
```

```
## Warning in .set_group_names(grl, use.names, txdb, by): some group names are NAs
## or duplicated
```

```
sig_damage_uniform <- signature_potential_damage_analysis(signatures[, c("SBS1", "SBS5","SBS7b","SBS7a","Psoralen
")], contexts, context_mismatches)
sig_damage_sbs7b <- signature_potential_damage_analysis_sbs7b_ref(signatures[, c("SBS1", "SBS5","SBS7b","SBS7a","
Psoralen")], contexts, context_mismatches)



sig_damage_uniform$Reference <- "Uniform mutation rate"
sig_damage_sbs7b$Reference <- "SBS7b"
sig_damage <- rbind(sig_damage_uniform, sig_damage_sbs7b)
sig_damage$sig[sig_damage$sig=="PUVA"] <- "Psoralen"

sig_damage$type <- factor(sig_damage$type,
                          levels=c("Synonymous", "Missense", "Stop_gain", "splice_site"),
                          labels=c("Synonymous", "Missense", "Nonsense", "Splice Site"))

sig_damage$Reference <- factor(sig_damage$Reference, levels=c("Uniform mutation rate", "SBS7b"))

write.table(sig_damage, "/nfs/users/nfs_s/so11/phd/psoriasis/bsub_jupyter_lab/psoriasis/manuscript_data_and_figur
es/signature_potential_damage.txt", sep="\t", quote=F)

## Nonsense,Splice, Missense, Indels, synonymous
annotation_cols <- c("#5D378E","#7155A5","#6A9A9E","#C0843E","#A9ACAB")

ggplot(sig_damage, aes(x=sig, y=ratio_by_background, fill=type)) +
  geom_bar(position="dodge", stat="identity") + theme_classic()+
  geom_hline(yintercept = 1, linetype="dashed") +
  scale_y_continuous(expand=c(0,0), limits = c(0,1.8)) + labs(x="", y="Ratio of mutation type \n caused by signat
ure \n vs background ", fill="") + facet_wrap(~Reference) +
  scale_fill_manual(values=annotation_cols) + theme(legend.position = "bottom")
```

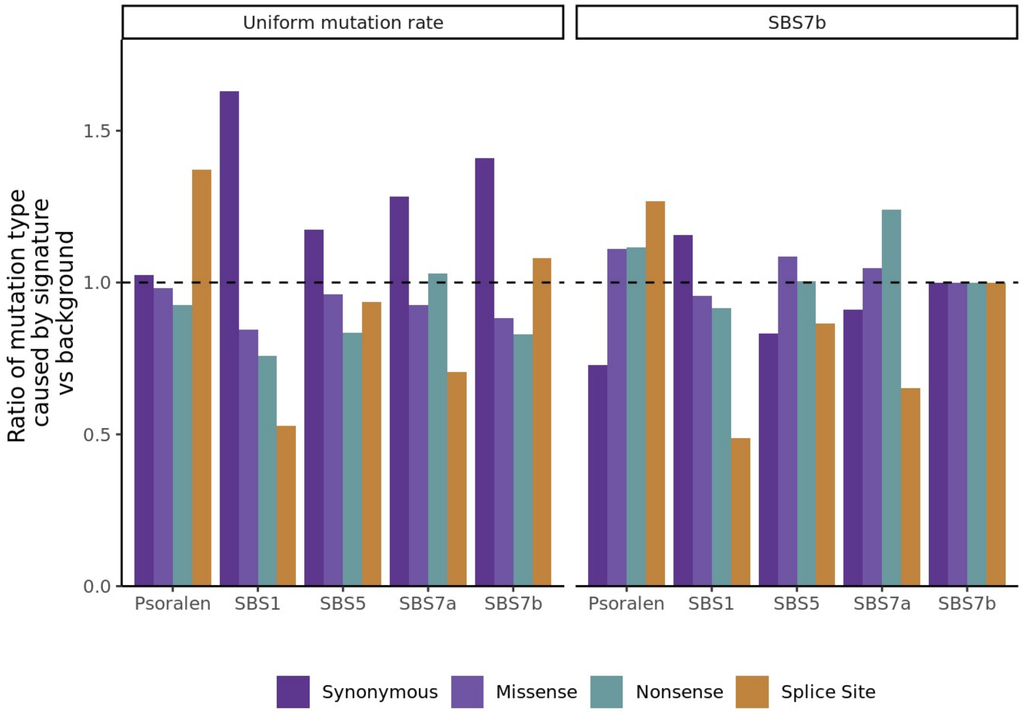

Uniform mutation rate
SBS7b
Ratio of mutation type caused by signature vs background
1.5
1.0
0.5
0.0
Psoralen
SBS1
SBS5
SBS7a
SBS7b
Psoralen
SBS1
SBS5
SBS7a
SBS7b
Synonymous
Missense
Nonsense
Splice Site

Supplementary Note 6:

An R-markdown notebook describing the selection analyses.

Code S4: Selection analyses

Sigurgeir Olafsson

2/17/2022

Introduction

This document describes the selection analyses carried out as part of the manuscript "Effects of psoriasis and phototreatment on the somatic mutation landscape of the skin" by Sigurgeir Ólafsson et al.

For selection analyses, we used the dNdScv software. Please see https://doi.org/10.1016/j.cell.2017.09.042 (https://doi.org/10.1016/j.cell.2017.09.042) and https://github.com/im3sanger/dndscv (https://github.com/im3sanger/dndscv)

The analyses use mutation calls provided in Supplementary Table 4 of the manuscript. The raw sequencing data has been made publicly available, please see the manuscript for details.

```
.libPaths("/lustre/scratch126/humgen/projects/psoriasis/R_packages_farm5_R4.1.0_install/")

library("seqinr")
library("Biostrings")
library("MASS")
library("GenomicRanges")
library("dndscv")
```

```
all_mutations <- read.table("/nfs/users/nfs_s/so11/phd/psoriasis/bsub_jupyter_lab/psoriasis/manuscript_data_and_f
igures/Supplementary_Table4_all_mutations.txt", h=T)
head(all_mutations)
```

```
##   PatientID         MicrobiopsyID    ClusterID             MutationID  Chr
## 1 patient01    P01L_LL_10,P01L_17 patient01_36  P01:chr1:10018498:A:T chr1
## 2 patient01               P01L_16  patient01_4 P01:chr1:100268476:A:C chr1
## 3 patient01            P01L_LL_13 patient01_28 P01:chr1:100348186:G:A chr1
## 4 patient01               P01L_20 patient01_38 P01:chr1:100743353:G:A chr1
## 5 patient01             P01H_LL_8 patient01_20   P01:chr1:1007997:C:T chr1
## 6 patient01 P01L_LL_11,P01L_LL_14 patient01_33 P01:chr1:100941476:C:T chr1
##    Pos_hg38 Ref Alt type
## 1  10018498   A   T  SBS
## 2 100268476   A   C  SBS
## 3 100348186   G   A  SBS
## 4 100743353   G   A  SBS
## 5   1007997   C   T  SBS
## 6 100941476   C   T  SBS
```

```
table(all_mutations$type)
```

```
##
##    DBS  Indel    SBS
##  54537   3670 582754
```

Exome wide test for selection on the gene-level

I will first conduct an unbiased test for positive selection on the level of individual genes. dNdScv does not annotate double-base mutations (DBSs) but lumps them together with indels under a "no-SNV" mutation class. Many datasets have few DBS mutations so this is not a big issue. As DBS mutations commonly occur as a result of UV-exposure, the current dataset has many such mutations and these need to be accounted for in the modeling.

DBS mutations are much more numerous than indels. I will run a separate negative binomial model for each mutation class. This involves running dNdScv twice, once excluding indels and once excluding DBS mutations. The results for single-base substitutions (SBSs) are unaffected. The P-values from each model can then be combined using Fisher's method.

```
## Format the data a little
all_mutations$Chr <- gsub("chr", "", all_mutations$Chr)
dbs_only <- all_mutations[all_mutations$type!="Indel",c("MicrobiopsyID", "Chr", "Pos_hg38", "Ref", "Alt")]
indel_only <- all_mutations[all_mutations$type!="DBS",c("MicrobiopsyID", "Chr", "Pos_hg38", "Ref", "Alt")]
colnames(dbs_only) = colnames(indel_only) = c("SampleID", "Chr", "Pos", "Ref", "Alt")

## Read in the covariates for dNdScv
covs = "/lustre/scratch126/humgen/projects/psoriasis/resources/covariates_20pc_GRCh37-38.altogether_withoutepiout
.Rdat"
load(covs) # it loads an object called scores

refcds_38 = "/lustre/scratch126/humgen/projects/psoriasis/resources/refcds_GRCh38-GencodeV18+Appris.rda"
```

There are a few samples that truly have a very high mutation burden. I will overwrite the default parameters for `max_muts_per_gene_per_sample` and `max_coding_muts_per_sample` to include all mutations. Below, I show that the inclusion of hypermutators does not affect the results.

```
d38_dbs_only = dndscv(dbs_only, refdb=refcds_38, cv=scores,max_muts_per_gene_per_sample = Inf, max_coding_muts_pe
r_sample = Inf)
d38_indel_only = dndscv(indel_only, refdb=refcds_38, cv=scores,max_muts_per_gene_per_sample = Inf, max_coding_mut
s_per_sample = Inf)


sel_cv_dbs = d38_dbs_only$sel_cv
sel_cv_indel = d38_indel_only$sel_cv
# Results are sorted by significance - sort the dataframes in the same way:
sel_cv_indel = sel_cv_indel[order(sel_cv_indel$gene_name),]
sel_cv_dbs = sel_cv_dbs[order(sel_cv_dbs$gene_name),]

# Fisher combined p-values (single base substitutions, double base substitutions and indels)
p_global <- 1-pchisq(-2 * (log(sel_cv_indel$pallsubs_cv) + log(sel_cv_indel$pind_cv) + log(sel_cv_dbs$pind_cv)),
df = 6)
q_global <- p.adjust(p_global, method="BH")

sel_cv_indel$pdbs_cv <- sel_cv_dbs$pind_cv
sel_cv_indel$wdbs_cv <- sel_cv_dbs$wind_cv
sel_cv_indel$pglobal_cv <- p_global
sel_cv_indel$qglobal_cv <- q_global
sel_cv_indel$n_dbs <- sel_cv_dbs$n_ind

sel_cv_indel = sel_cv_indel[order(sel_cv_indel$pglobal_cv, sel_cv_indel$pallsubs_cv, sel_cv_indel$pmis_cv, sel_cv
_indel$ptrunc_cv, -sel_cv_indel$wmis_cv),] # Sorting genes in the output file

signif_genes <- sel_cv_indel[sel_cv_indel$qglobal_cv < 0.05,]
signif_genes
```

```
##       gene_name n_syn n_mis n_non n_spl n_ind  wmis_cv   wnon_cv   wspl_cv
## 10643    NOTCH1    14   108    33    28    14 7.079368 54.401364 54.401364
## 5513       FAT1    14    53    48    15    31 1.729644 31.579216 31.579216
## 12602     PPM1D     1     0    14     0     2 0.000000 54.060797 54.060797
## 17180      TP53     1    23     5     0     3 8.888560 19.577054 19.577054
## 10644    NOTCH2     7    33     8     2     6 3.140914  9.542485  9.542485
## 2999      CHEK2     2    15     3     3     0 5.782366 16.531743 16.531743
## 6776     GXYLT1     0     7     5     2     1 3.927487 26.464145 26.464145
## 18571   ZFP36L2     6    14     1     0     4 4.244585 12.068969 12.068969
## 4736     EEF1A1     4    11     1     1     1 4.556871  8.637449  8.637449
##         wind_cv      pmis_cv    ptrunc_cv  pallsubs_cv      pind_cv
## 10643 19.795603 0.000000e+00 0.000000e+00 0.000000e+00 1.731752e-08
## 5513  22.792296 1.926672e-02 0.000000e+00 0.000000e+00 4.599866e-13
## 12602  8.771611 3.468602e-02 0.000000e+00 0.000000e+00 2.791332e-02
## 17180 20.968633 2.600571e-10 1.531432e-05 5.375256e-12 9.622535e-04
## 10644 10.378967 4.881740e-05 1.150806e-06 7.853484e-08 4.733263e-04
## 2999   0.000000 7.143712e-06 6.336257e-06 2.075037e-08 1.000000e+00
## 6776   5.703350 8.533551e-03 6.659677e-08 7.329042e-08 1.560063e-01
## 18571 16.934491 1.504069e-04 7.826796e-02 2.304162e-04 4.381137e-04
## 4736   5.472396 3.756794e-04 2.688436e-02 2.879537e-04 1.618246e-01
##            qmis_cv    qtrunc_cv  qallsubs_cv   pglobal_cv   qglobal_cv
## 10643 0.000000e+00 0.0000000000 0.000000e+00 0.000000e+00 0.000000e+00
## 5513  7.862315e-01 0.0000000000 0.000000e+00 0.000000e+00 0.000000e+00
## 12602 7.862315e-01 0.0000000000 0.000000e+00 0.000000e+00 0.000000e+00
## 17180 2.498628e-06 0.0403587599 2.582273e-08 0.000000e+00 0.000000e+00
## 10644 1.595753e-01 0.0044227774 2.155894e-04 4.004908e-12 1.539166e-08
## 2999  4.575785e-02 0.0202929194 7.974781e-05 1.390695e-07 4.453933e-04
## 6776  7.862315e-01 0.0003199309 2.155894e-04 2.132264e-06 5.853369e-03
## 18571 3.211355e-01 0.8213585315 2.951785e-01 3.188282e-06 7.658252e-03
## 4736  5.508156e-01 0.8213585315 3.254894e-01 1.207730e-05 2.578639e-02
##            pdbs_cv   wdbs_cv n_dbs
## 10643 6.807617e-11  7.058805    36
## 5513  2.362045e-04  3.346236    23
## 12602 4.740368e-04  9.110472     5
## 17180 1.545748e-10 17.314951    13
## 10644 1.909303e-04  5.289073    10
## 2999  2.688338e-02  4.978598     3
## 6776  1.000000e+00  0.000000     0
## 18571 1.775103e-01  2.650694     2
## 4736  1.718188e-03  8.876233     4
```

```
# No need to re-write the file every time the document is knitted.
#write.table(sel_cv_indel, file="/nfs/users/nfs_s/so11/phd/psoriasis/bsub_jupyter_lab/psoriasis/manuscript_data_a
nd_figures/sel_cv_dNdS_results.txt", sep="\t", quote=F, row.names = F)
```

Test the effect of excluding the covariates

It's worth checking what effect the covariates have on the above analysis. This is done simply by setting the cv parameter to NULL in dndscv(). The covariates have been extensively tested in TCGA and they generally give superior results. I report the selection analysis *with* covariates in the paper.

```
dbs_noCov = dndscv(dbs_only, refdb=refcds_38, cv=NULL,max_muts_per_gene_per_sample = Inf, max_coding_muts_per_sam
ple = Inf)
indel_noCov = dndscv(indel_only, refdb=refcds_38, cv=NULL,max_muts_per_gene_per_sample = Inf, max_coding_muts_per
_sample = Inf)

sel_cv_dbs_noCov = dbs_noCov$sel_cv
sel_cv_indel_noCov = indel_noCov$sel_cv
# Sort the dataframes in the same way
sel_cv_indel_noCov = sel_cv_indel_noCov[order(sel_cv_indel_noCov$gene_name),]
sel_cv_dbs_noCov = sel_cv_dbs_noCov[order(sel_cv_dbs_noCov$gene_name),]

p_global <- 1-pchisq(-2 * (log(sel_cv_indel_noCov$pallsubs_cv) + log(sel_cv_indel_noCov$pind_cv) + log(sel_cv_dbs
_noCov$pind_cv)), df = 6)
q_global <- p.adjust(p_global, method="BH")

sel_cv_indel_noCov$pdbs_cv <- sel_cv_dbs_noCov$pind_cv
sel_cv_indel_noCov$wdbs_cv <- sel_cv_dbs_noCov$wind_cv
sel_cv_indel_noCov$pglobal_cv <- p_global
sel_cv_indel_noCov$qglobal_cv <- q_global
sel_cv_indel_noCov$n_dbs <- sel_cv_dbs_noCov$n_ind

sel_cv_indel_noCov = sel_cv_indel_noCov[order(sel_cv_indel_noCov$pglobal_cv, sel_cv_indel_noCov$pallsubs_cv, sel_
cv_indel_noCov$pmis_cv, sel_cv_indel_noCov$ptrunc_cv, -sel_cv_indel_noCov$wmis_cv),] # Sorting genes in the outpu
t file

signif_genes_noCov <- sel_cv_indel_noCov[sel_cv_indel_noCov$qglobal_cv < 0.05,]
# Print out genes that are significant in either analysis.
sel_cv_indel_noCov[sel_cv_indel_noCov$gene_name %in% unique(c(signif_genes$gene_name),signif_genes_noCov$gene_nam
e),]
```

```
##       gene_name n_syn n_mis n_non n_spl n_ind   wmis_cv   wnon_cv   wspl_cv
## 10643    NOTCH1    14   108    33    28    14  5.882644 45.205142 45.205142
## 5513       FAT1    14    53    48    15    31  1.941906 35.454616 35.454616
## 12602     PPM1D     1     0    14     0     2  0.000000 42.958067 42.958067
## 17180      TP53     1    23     5     0     3 10.523903 23.178898 23.178898
## 10644    NOTCH2     7    33     8     2     6  2.478091  7.528747  7.528747
## 6776     GXYLT1     0     7     5     2     1  3.846491 25.918381 25.918381
## 18571   ZFP36L2     6    14     1     0     4  3.005842  8.546752  8.546752
## 2999      CHEK2     2    15     3     3     0  4.918302 14.061392 14.061392
## 4736     EEF1A1     4    11     1     1     1  3.523855  6.679390  6.679390
##         wind_cv      pmis_cv    ptrunc_cv  pallsubs_cv      pind_cv
## 10643 18.086176 0.000000e+00 0.000000e+00 0.000000e+00 1.066297e-07
## 5513  22.306077 8.792866e-03 0.000000e+00 0.000000e+00 5.120150e-12
## 12602 10.897753 2.492705e-02 8.437695e-15 0.000000e+00 1.956446e-02
## 17180 25.142277 1.802082e-09 1.261253e-05 8.893708e-11 6.374008e-04
## 10644  8.014609 3.891458e-03 1.721945e-05 2.899751e-05 1.884998e-03
## 6776   7.487571 1.913497e-02 2.983930e-07 9.874783e-07 1.216245e-01
## 18571 26.682982 6.000522e-03 1.167973e-01 9.690697e-03 9.891691e-05
## 2999   0.000000 2.119866e-04 3.105269e-05 3.455001e-06 1.000000e+00
## 4736   7.131791 5.137191e-03 4.782408e-02 5.643228e-03 1.271187e-01
##            qmis_cv    qtrunc_cv  qallsubs_cv   pglobal_cv   qglobal_cv
## 10643 0.0000000000 0.000000e+00 0.000000e+00 0.000000e+00 0.0000000000
## 5513  0.7610978496 0.000000e+00 0.000000e+00 0.000000e+00 0.0000000000
## 12602 0.7695392807 5.404625e-11 0.000000e+00 0.000000e+00 0.0000000000
## 17180 0.0000173144 4.847246e-02 4.272537e-07 0.000000e+00 0.0000000000
## 10644 0.6083139263 5.514816e-02 6.965202e-02 1.645490e-07 0.0006323949
## 6776  0.7695392807 1.433480e-03 3.795077e-03 1.728211e-05 0.0394374564
## 18571 0.7030855804 8.124141e-01 9.291924e-01 1.873004e-05 0.0394374564
## 2999  0.2017609298 7.458856e-02 1.106522e-02 2.052324e-05 0.0394374564
## 4736  0.6969008351 8.124141e-01 8.421045e-01 3.776899e-04 0.5200043269
##            pdbs_cv    wdbs_cv n_dbs
## 10643 1.790345e-11 11.113408    36
## 5513  5.228709e-04  3.954717    23
## 12602 3.173290e-03  6.510330     5
## 17180 3.630909e-11 26.034710    13
## 10644 1.227763e-02  3.191958    10
## 6776  1.000000e+00  0.000000     0
## 18571 1.372586e-01  3.188089     2
## 2999  4.224463e-02  4.351390     3
## 4736  5.847797e-03  6.816864     4
```

Test the effect of hypermutators

Hypermutators can have an effect on selection analyses as they contribute many passengers but relatively few drivers. I want to test the effect of excluding hypermutators. This involves doing two things: First, remove the samples from P34H, which have an incredibly high mutation burden. Second, account for other possible hypermutators by using the default settings for max_muts_per_gene and sample.

```
dbs_noHype <- dbs_only[grep("P34H", dbs_only$SampleID, invert=T),]
indel_noHype <- indel_only[grep("P34H", indel_only$SampleID, invert=T),]

dbs_hype = dndscv(dbs_noHype, refdb=refcds_38, cv=scores)
indel_hype = dndscv(indel_noHype, refdb=refcds_38, cv=scores)

sel_cv_dbs_hype = dbs_hype$sel_cv
sel_cv_indel_hype = indel_hype$sel_cv

sel_cv_indel_hype = sel_cv_indel_hype[order(sel_cv_indel_hype$gene_name),]
sel_cv_dbs_hype = sel_cv_dbs_hype[order(sel_cv_dbs_hype$gene_name),]

p_global <- 1-pchisq(-2 * (log(sel_cv_indel_hype$pallsubs_cv) + log(sel_cv_indel_hype$pind_cv) + log(sel_cv_dbs_h
ype$pind_cv)), df = 6)
q_global <- p.adjust(p_global, method="BH")

sel_cv_indel_hype$pdbs_cv <- sel_cv_dbs_hype$pind_cv
sel_cv_indel_hype$wdbs_cv <- sel_cv_dbs_hype$wind_cv
sel_cv_indel_hype$pglobal_cv <- p_global
sel_cv_indel_hype$qglobal_cv <- q_global
sel_cv_indel_hype$n_dbs <- sel_cv_dbs_hype$n_ind

sel_cv_indel_hype = sel_cv_indel_hype[order(sel_cv_indel_hype$pglobal_cv, sel_cv_indel_hype$pallsubs_cv, sel_cv_i
ndel_hype$pmis_cv, sel_cv_indel_hype$ptrunc_cv, -sel_cv_indel_hype$wmis_cv),] # Sorting genes in the output file

# Print out genes that are significant in either analysis.
signif_genes_hype <- sel_cv_indel_hype[sel_cv_indel_hype$qglobal_cv<0.05,]
sel_cv_indel_hype[sel_cv_indel_hype$gene_name %in% unique(c(signif_genes$gene_name),signif_genes_hype$gene_name),
]
```

```
##       gene_name n_syn n_mis n_non n_spl n_ind  wmis_cv   wnon_cv   wspl_cv
## 10643    NOTCH1    14   108    32    28    14 7.120927 54.003918 54.003918
## 5513       FAT1    14    50    48    14    31 1.650095 31.557558 31.557558
## 12602     PPM1D     1     0    13     0     2 0.000000 50.994060 50.994060
## 17180      TP53     1    23     5     0     3 8.954853 19.897862 19.897862
## 10644    NOTCH2     7    33     8     2     6 3.171931  9.709661  9.709661
## 2999      CHEK2     2    15     3     3     0 5.878076 17.067400 17.067400
## 6776     GXYLT1     0     7     5     2     1 4.016087 27.125340 27.125340
## 18571   ZFP36L2     6    14     1     0     4 4.304309 12.275375 12.275375
## 4736     EEF1A1     4    11     1     1     1 4.625159  8.836645  8.836645
##         wind_cv      pmis_cv    ptrunc_cv  pallsubs_cv      pind_cv
## 10643 19.924305 0.000000e+00 0.000000e+00 0.000000e+00 1.524526e-08
## 5513  22.959330 3.482043e-02 0.000000e+00 0.000000e+00 3.484356e-13
## 12602  8.849431 3.559521e-02 2.220446e-16 0.000000e+00 2.743666e-02
## 17180 21.413216 2.260823e-10 1.412898e-05 4.376943e-12 9.023988e-04
## 10644 10.470245 4.183010e-05 9.855678e-07 6.065391e-08 4.472788e-04
## 2999   0.000000 5.943054e-06 5.267843e-06 1.486805e-08 1.000000e+00
## 6776   5.755292 7.607241e-03 5.615772e-08 5.716063e-08 1.547987e-01
## 18571 16.992137 1.312106e-04 7.679084e-02 1.999518e-04 4.286992e-04
## 4736   5.503878 3.329499e-04 2.567056e-02 2.483905e-04 1.610536e-01
##            qmis_cv    qtrunc_cv  qallsubs_cv   pglobal_cv   qglobal_cv
## 10643 0.000000e+00 0.000000e+00 0.000000e+00 0.000000e+00 0.000000e+00
## 5513  7.874679e-01 0.000000e+00 0.000000e+00 0.000000e+00 0.000000e+00
## 12602 7.874679e-01 1.422270e-12 0.000000e+00 0.000000e+00 0.000000e+00
## 17180 2.172198e-06 3.807097e-02 2.102684e-08 0.000000e+00 0.000000e+00
## 10644 1.607615e-01 3.787734e-03 1.665037e-04 2.923328e-12 1.123494e-08
## 2999  3.806724e-02 1.687115e-02 5.714088e-05 1.022645e-07 3.275192e-04
## 6776  7.874679e-01 2.697817e-04 1.665037e-04 1.694158e-06 4.650704e-03
## 18571 2.801492e-01 8.234364e-01 2.708333e-01 2.757423e-06 6.623330e-03
## 4736  4.921512e-01 8.234364e-01 2.983170e-01 1.051551e-05 2.245179e-02
##            pdbs_cv   wdbs_cv n_dbs
## 10643 6.551400e-11  7.059927    36
## 5513  2.293538e-04  3.352013    23
## 12602 4.731561e-04  9.110320     5
## 17180 1.516628e-10 17.324706    13
## 10644 1.872143e-04  5.299507    10
## 2999  2.677542e-02  4.985733     3
## 6776  1.000000e+00  0.000000     0
## 18571 1.777303e-01  2.648388     2
## 4736  1.711674e-03  8.882732     4
```

Restricting the analysis to lesional skin

We may wonder if pooling samples from lesional and non-lesional skin impacts the analysis. The following analysis shows that no recurrently mutated genes are missed by pooling samples but as the sample size is smaller and there are fewer mutations, some genes no longer reach significance.

```
dbs_lesional <- dbs_only[!grepl("H", dbs_only$SampleID),]
d38_dbs_lesional = dndscv(dbs_lesional, refdb=refcds_38, cv=scores,max_muts_per_gene_per_sample = Inf, max_coding
_muts_per_sample = Inf)
sel_cv_dbs = d38_dbs_lesional$sel_cv

indel_lesional <- indel_only[!grepl("H", indel_only$SampleID),]
d38_indel_lesional = dndscv(indel_lesional, refdb=refcds_38, cv=scores,max_muts_per_gene_per_sample = Inf, max_co
ding_muts_per_sample = Inf)
sel_cv_indel_lesional = d38_indel_lesional$sel_cv


sel_cv_indel_lesional = sel_cv_indel_lesional[order(sel_cv_indel_lesional$gene_name),]
sel_cv_dbs = sel_cv_dbs[order(sel_cv_dbs$gene_name),]

p_global <- 1-pchisq(-2 * (log(sel_cv_indel_lesional$pallsubs_cv) + log(sel_cv_indel_lesional$pind_cv) + log(sel_
cv_dbs$pind_cv)), df = 6)
q_global <- p.adjust(p_global, method="BH")

sel_cv_indel_lesional$pdbs_cv <- sel_cv_dbs$pind_cv
sel_cv_indel_lesional$wdbs_cv <- sel_cv_dbs$wind_cv
sel_cv_indel_lesional$pglobal_cv <- p_global
sel_cv_indel_lesional$qglobal_cv <- q_global
sel_cv_indel_lesional$n_dbs <- sel_cv_dbs$n_ind

sel_cv_indel_lesional = sel_cv_indel_lesional[order(sel_cv_indel_lesional$pglobal_cv, sel_cv_indel_lesional$palls
ubs_cv, sel_cv_indel_lesional$pmis_cv, sel_cv_indel_lesional$ptrunc_cv, -sel_cv_indel_lesional$wmis_cv),] # Sorti
ng genes in the output file

signif_genes_lesional <- sel_cv_indel_lesional[sel_cv_indel_lesional$qglobal_cv < 0.05,]

sel_cv_indel_lesional[sel_cv_indel_lesional$gene_name %in% unique(c(signif_genes$gene_name),signif_genes_lesional
$gene_name),]
```

```
##       gene_name n_syn n_mis n_non n_spl n_ind  wmis_cv  wnon_cv  wspl_cv
## 10643    NOTCH1    13    84    27    18    11 7.019856 51.51734 51.51734
## 5513       FAT1     9    36    38    12    19 1.690002 36.58948 36.58948
## 17180      TP53     1    16     4     0     1 8.099085 20.79898 20.79898
## 12602     PPM1D     1     0    10     0     2 0.000000 51.12939 51.12939
## 10644    NOTCH2     5    22     6     2     4 2.949814 10.89947 10.89947
## 2999      CHEK2     2    12     3     3     0 6.078629 22.15425 22.15425
## 4736     EEF1A1     3     8     1     1     1 4.581142 12.10417 12.10417
## 18571   ZFP36L2     4    10     1     0     3 4.393832 17.65646 17.65646
## 6776     GXYLT1     0     4     3     2     1 2.989310 25.40303 25.40303
##         wind_cv      pmis_cv    ptrunc_cv  pallsubs_cv      pind_cv
## 10643 20.663841 0.000000e+00 0.000000e+00 0.000000e+00 2.979685e-08
## 5513  17.795820 5.082614e-02 0.000000e+00 0.000000e+00 3.882490e-10
## 17180  9.683031 8.124996e-08 7.504527e-05 1.903884e-09 9.673791e-02
## 12602 11.805702 6.162730e-02 2.944311e-13 1.342260e-13 1.559047e-02
## 10644  8.947100 7.827596e-04 4.379900e-06 1.152512e-06 3.101438e-03
## 2999   0.000000 2.127829e-05 1.116898e-06 8.017294e-09 1.000000e+00
## 4736   7.534266 1.755221e-03 1.355400e-02 6.004712e-04 1.220910e-01
## 18571 18.396571 7.680201e-04 5.118730e-02 7.126999e-04 1.184621e-03
## 6776   7.661010 8.596480e-02 4.364987e-06 1.159419e-05 1.202329e-01
##            qmis_cv    qtrunc_cv  qallsubs_cv   pglobal_cv   qglobal_cv
## 10643 0.0000000000 0.000000e+00 0.000000e+00 0.000000e+00 0.000000e+00
## 5513  0.8027364762 0.000000e+00 0.000000e+00 0.000000e+00 0.000000e+00
## 17180 0.0007806496 1.442070e-01 9.146260e-06 1.332268e-15 8.533618e-12
## 12602 0.8027364762 1.885930e-09 8.597620e-10 1.023626e-13 4.917498e-10
## 10644 0.6267295131 1.402736e-02 3.691112e-03 2.628387e-09 1.010142e-05
## 2999  0.0798764075 5.365578e-03 3.081206e-05 1.618688e-07 5.184117e-04
## 4736  0.7847006786 8.459965e-01 5.357220e-01 5.848092e-06 1.605385e-02
## 18571 0.6267295131 8.459965e-01 5.629391e-01 4.214596e-05 8.998630e-02
## 6776  0.8027364762 1.402736e-02 3.182770e-02 1.469048e-04 2.566293e-01
##            pdbs_cv   wdbs_cv n_dbs
## 10643 3.983118e-11  8.026094    31
## 5513  8.123731e-04  3.320814    17
## 17180 8.442670e-09 17.785926    10
## 12602 6.963941e-02  4.770846     2
## 10644 2.071016e-03  4.762052     7
## 2999  8.220890e-02  4.321640     2
## 4736  4.824407e-04 12.667308     4
## 18571 3.923593e-01  1.962829     1
## 6776  1.000000e+00  0.000000     0
```

Restricting the analysis to non-lesional skin

We find positive selection in a few genes that have not been previously reported for normal skin. The question we want to answer is if there is evidence for mutations in these genes being positively selected in non-lesional skin. If there is then that is evidence that these mutations have little to do with psoriasis itself.

```
dbs_nonLes <- dbs_only[grepl("H", dbs_only$SampleID),]
d38_dbs_nonLes = dndscv(dbs_nonLes, refdb=refcds_38, cv=scores,max_muts_per_gene_per_sample = Inf, max_coding_mut
s_per_sample = Inf)
sel_cv_dbs = d38_dbs_nonLes$sel_cv

indel_nonLes <- indel_only[grepl("H", indel_only$SampleID),]
d38_indel_nonLes = dndscv(indel_nonLes, refdb=refcds_38, cv=scores,max_muts_per_gene_per_sample = Inf, max_coding
_muts_per_sample = Inf)
sel_cv_indel_nonLes = d38_indel_nonLes$sel_cv


sel_cv_indel_nonLes = sel_cv_indel_nonLes[order(sel_cv_indel_nonLes$gene_name),]
sel_cv_dbs = sel_cv_dbs[order(sel_cv_dbs$gene_name),]

p_global <- 1-pchisq(-2 * (log(sel_cv_indel_nonLes$pallsubs_cv) + log(sel_cv_indel_nonLes$pind_cv) + log(sel_cv_d
bs$pind_cv)), df = 6)
q_global <- p.adjust(p_global, method="BH")

sel_cv_indel_nonLes$pdbs_cv <- sel_cv_dbs$pind_cv
sel_cv_indel_nonLes$wdbs_cv <- sel_cv_dbs$wind_cv
sel_cv_indel_nonLes$pglobal_cv <- p_global
sel_cv_indel_nonLes$qglobal_cv <- q_global
sel_cv_indel_nonLes$n_dbs <- sel_cv_dbs$n_ind

sel_cv_indel_nonLes = sel_cv_indel_nonLes[order(sel_cv_indel_nonLes$pglobal_cv, sel_cv_indel_nonLes$pallsubs_cv,
sel_cv_indel_nonLes$pmis_cv, sel_cv_indel_nonLes$ptrunc_cv, -sel_cv_indel_nonLes$wmis_cv),] # Sorting genes in th
e output file

signif_genes_nonLes <- sel_cv_indel_nonLes[sel_cv_indel_nonLes$qglobal_cv < 0.05,]

sel_cv_indel_nonLes[sel_cv_indel_nonLes$gene_name %in% c("CHEK2", "GXYLT1", "ZFP36L2", "EEF1A1"),]
```

```
##       gene_name n_syn n_mis n_non n_spl n_ind  wmis_cv wnon_cv wspl_cv  wind_cv
## 18571   ZFP36L2     2     4     0     0     1 5.727376  0.0000  0.0000 13.69636
## 6776     GXYLT1     0     3     2     0     0 6.131248 26.8669 26.8669  0.00000
## 2999      CHEK2     0     3     0     0     0 5.137455  0.0000  0.0000  0.00000
## 4736     EEF1A1     1     3     0     0     0 6.081441  0.0000  0.0000  0.00000
##          pmis_cv   ptrunc_cv pallsubs_cv    pind_cv   qmis_cv qtrunc_cv
## 18571 0.01088410 0.849444047 0.038185310 0.06792245 0.7711319 0.9213464
## 6776  0.02221642 0.002823397 0.001278334 1.00000000 0.7711319 0.9213464
## 2999  0.03578235 0.678473337 0.099061031 1.00000000 0.7711319 0.9213464
## 4736  0.02198161 0.754013666 0.068235954 1.00000000 0.7711319 0.9213464
##       qallsubs_cv pglobal_cv qglobal_cv   pdbs_cv  wdbs_cv n_dbs
## 18571   0.9396631 0.02054961   0.999999 0.2172717 4.044568     1
## 6776    0.9396631 0.03816420   0.999999 1.0000000 0.000000     0
## 2999    0.9396631 0.19297844   0.999999 0.1322319 7.013037     1
## 4736    0.9396631 0.49735927   0.999999 1.0000000 0.000000     0
```

Restricted hypothesis testing

Not all genes are equally likely to be under positive selection in the skin. The above analysis assumes we know nothing of what to expect, but in reality we do. Past studies of skin and oesophagous have identified a number of recurrently mutated genes and we may be interested in seeing if there is evidence of positive selection of mutations in those genes in the current dataset.

I have compiled a list of genes from Fowler et a. https://doi.org/10.1158/2159-8290.CD-20-1092 (https://doi.org/10.1158/2159-8290.CD-20-1092), Martincorena et al https://doi.org/10.1126/science.aau3879 (https://doi.org/10.1126/science.aau3879) and Yokoyama et al https://doi.org/10.1038/s41586-018-0811-x (https://doi.org/10.1038/s41586-018-0811-x).

The restricted hypothesis testing basically just involves only changing the q-values to reflect less multiple testing. Even in with much reduced burden of multiple testing, no evidence for selection in these genes is found.

```
rht_genes <- read.table("/nfs/users/nfs_s/so11/phd/psoriasis/bsub_jupyter_lab/psoriasis/08_selection_analyses/rht
_genes.txt")

sel_cv_rht <- sel_cv_indel[sel_cv_indel$gene_name %in% rht_genes$V1, ]
sel_cv_rht$qrht <- p.adjust(sel_cv_rht$pglobal_cv, method = "BH")
sel_cv_rht <- sel_cv_rht[order(sel_cv_rht$qrht),]
sel_cv_rht
```

```
##      gene_name n_syn n_mis n_non n_spl n_ind   wmis_cv   wnon_cv   wspl_cv
## 8302     KMT2D    17    30     7     3     0 0.8112327 3.8393209 3.8393209
## 1004     ARID2     1    12     5     0     1 1.5816826 6.8840196 6.8840196
```

```
## 13047     PTCH1     4    11     1     1     1 1.5675698 3.0423208 3.0423208
## 17197      TP63     1    12     0     2     0 3.5057851 5.2269680 5.2269680
## 10649    NOTCH3     6    25     3     3     1 1.7705010 5.5652371 5.5652371
## 3583     CREBBP     1    18     1     0     2 2.1309025 1.1990655 1.1990655
## 8089       KDM6A     1     2     0     0     2 0.4237191 0.0000000 0.0000000
## 12140    PIK3CA     4    14     0     0     0 2.2702234 0.0000000 0.0000000
## 1002     ARID1A     6     8     0     0     1 0.6832892 0.0000000 0.0000000
## 2487      CCND1     0     0     0     0     0 0.0000000 0.0000000 0.0000000
## 5001      EP300     6    21     1     0     1 1.7900089 0.8194030 0.8194030
## 12371    PLXNB2    10    10     0     0     2 1.1013011 0.0000000 0.0000000
## 4299     DICER1     2    15     0     0     0 1.7617321 0.0000000 0.0000000
## 10650    NOTCH4     4    13     0     0     1 1.1851198 0.0000000 0.0000000
## 490       AJUBA     1     5     1     0     0 1.9671710 6.6530024 6.6530024
## 16569    TGFBR2     3     1     0     1     0 0.3610332 3.8449175 3.8449175
## 19070    ZNF750     1     5     0     0     0 1.3716952 0.0000000 0.0000000
## 3822        CUL3     2     5     0     0     0 1.4922922 0.0000000 0.0000000
## 5596      FBXW7     0     2     0     0     0 0.7533226 0.0000000 0.0000000
## 10793      NSD1    11    11     1     0     0 0.5969390 0.6359945 0.6359945
## 13431       RB1     0     5     1     1     0 1.3947360 3.8404894 3.8404894
## 10448    NFE2L2     0     2     1     0     0 0.9031943 5.9928554 5.9928554
## 11693      PAX9     0     3     0     0     0 1.9836328 0.0000000 0.0000000
##        wind_cv     pmis_cv   ptrunc_cv pallsubs_cv    pind_cv   qmis_cv
## 8302  0.000000 0.423747277 0.001199005 0.001157984 1.00000000 0.8687378
## 1004  1.423183 0.257761127 0.002043402 0.007654828 0.46265517 0.8078884
## 13047 2.160820 0.260966230 0.197869932 0.278123848 0.34656500 0.8078884
## 17197 0.000000 0.002647366 0.074156242 0.004005285 1.00000000 0.6909816
## 10649 1.132877 0.063313739 0.001983061 0.004174063 0.53174567 0.7862315
## 3583  2.716761 0.037013443 0.864723967 0.111506158 0.17703278 0.7862315
## 8089  4.576859 0.212106319 0.266448198 0.271713827 0.08261487 0.8077863
## 12140 0.000000 0.028949064 0.164511967 0.024510496 1.00000000 0.7862315
## 1002  1.328253 0.361009861 0.188262736 0.306972773 0.48327636 0.8404194
## 2487  0.000000 0.167781695 0.687645375 0.358486997 1.00000000 0.8001252
## 5001  1.126011 0.070785906 0.841741268 0.172128507 0.53361609 0.7862315
## 12371 2.693266 0.801855841 0.203629160 0.419686169 0.17910967 0.9753646
## 4299  0.000000 0.130536871 0.185651615 0.101434727 1.00000000 0.7862315
## 10650 1.844645 0.651615512 0.162683133 0.312238304 0.38851229 0.9467775
## 490   0.000000 0.224889286 0.153632455 0.194397853 1.00000000 0.8077863
## 16569 0.000000 0.251218596 0.277827268 0.265169546 1.00000000 0.8077863
## 19070 0.000000 0.558988410 0.564925155 0.704452851 1.00000000 0.9170269
## 3822  0.000000 0.458455231 0.369679601 0.488034486 1.00000000 0.8832641
## 5596  0.000000 0.701960137 0.388635400 0.651215126 1.00000000 0.9589213
## 10793 0.000000 0.149819003 0.634541079 0.342711928 1.00000000 0.7864592
## 13431 0.000000 0.543112939 0.133456394 0.298083414 1.00000000 0.9130652
## 10448 0.000000 0.892865787 0.172885461 0.384900735 1.00000000 0.9916753
## 11693 0.000000 0.323956512 0.654613139 0.550002454 1.00000000 0.8265948
##       qtrunc_cv qallsubs_cv pglobal_cv qglobal_cv    pdbs_cv   wdbs_cv n_dbs
## 8302  0.8213585   0.7178008 0.01021164          1 0.19822676 1.3771606    16
## 1004  0.8213585   0.9297071 0.02758551          1 0.23416021 1.8222291     3
## 13047 0.8213585   0.9297071 0.03379491          1 0.01126764 3.3536965     7
## 17197 0.8213585   0.9297071 0.04614439          1 0.41265083 1.8363818     1
## 10649 0.8213585   0.9297071 0.04771649          1 0.77942867 0.6645653     3
## 3583  0.8824501   0.9297071 0.07193081          1 0.15463631 1.7608003     6
## 8089  0.8213585   0.9297071 0.11508058          1 0.26682758 1.9842640     2
## 12140 0.8213585   0.9297071 0.28397151          1 1.00000000 0.0000000     0
## 1002  0.8213585   0.9297071 0.31735009          1 0.19972340 1.6754952     5
## 2487  0.8213585   0.9297071 0.32699631          1 0.08706286 4.1714187     2
## 5001  0.8653844   0.9297071 0.48469877          1 0.70537923 0.7617713     2
## 12371 0.8213585   0.9297071 0.51350321          1 0.96883266 0.2473016     1
## 4299  0.8213585   0.9297071 0.55880531          1 0.85766380 0.4709775     1
## 10650 0.8213585   0.9297071 0.62488659          1 0.92082930 0.3527044     1
## 490   0.8213585   0.9297071 0.62698658          1 0.57913019 1.1128618     1
## 16569 0.8213585   0.9297071 0.62807571          1 0.42629012 1.7569310     1
## 19070 0.8213585   0.9297071 0.63630646          1 0.16545076 2.2013777     3
## 3822  0.8213585   0.9297071 0.81817569          1 0.47462644 1.5108812     1
## 5596  0.8213585   0.9297071 0.83875448          1 0.38703941 2.0002260     1
## 10793 0.8213585   0.9297071 0.86603150          1 0.82697257 0.5711352     2
## 13431 0.8213585   0.9297071 0.87722792          1 1.00000000 0.0000000     0
## 10448 0.8213585   0.9297071 0.92782728          1 1.00000000 0.0000000     0
## 11693 0.8213585   0.9297071 0.97709829          1 1.00000000 0.0000000     0
##            qrht
## 8302  0.2194959
## 1004  0.2194959
## 13047 0.2194959
## 17197 0.2194959
## 10649 0.2194959
## 3583  0.2757348
## 8089  0.3781219
## 12140 0.7520915
## 1002  0.7520915
```

```
## 2487  0.7520915
## 5001  0.8608852
## 12371 0.8608852
## 4299  0.8608852
## 10650 0.8608852
## 490   0.8608852
## 16569 0.8608852
## 19070 0.8608852
## 3822  0.9607734
## 5596  0.9607734
## 10793 0.9607734
## 13431 0.9607734
## 10448 0.9700012
## 11693 0.9770983
```

Site- dN/dS

I want to run site-wise dN/dS to identify mutation hotspots that may be found in genes that do not reach significance on the gene level. The following analysis shows that only NOTCH1 E455K reaches significance.

```
d38_sites = dndscv(dbs_only, refdb=refcds_38, cv=scores,max_muts_per_gene_per_sample = Inf, max_coding_muts_per_s
ample = Inf, outmats=T)
sites=sitednds(d38_sites)
head(sites$recursites)
```

```
##   chr       pos ref mut      gene aachange     impact ref3_cod mut3_cod freq
## 1   9 136517830   C   T    NOTCH1    E455K   Missense      CGA      CAA    7
## 2   9 136518238   G   A    NOTCH1    S385F   Missense      TCC      TTC    5
## 3  20  33663730   G   A C20orf144    E109K   Missense      AGA      AAA    4
## 4  12 120738875   G   A     ACADS    R330H   Missense      CGC      CAC    3
## 5   2 179483369   C   T   ZNF385B    T206T Synonymous      CGA      CAA    5
## 6  12  85980280   C   T    MGAT4C    R149H   Missense      CGT      CAT    4
##            mu      dnds         pval         qval
## 1 0.010021673  698.4861 1.344934e-13 1.376329e-05
## 2 0.012524985  399.2021 1.508268e-09 6.454599e-02
## 3 0.004917534  813.4159 1.892209e-09 6.454599e-02
## 4 0.001163337 2578.7883 3.302796e-09 7.516383e-02
## 5 0.015244463  327.9879 3.941409e-09 7.516383e-02
## 6 0.006086329  657.2106 4.406956e-09 7.516383e-02
```

Pathway-level dN/dS

In our recent manuscript "Somatic evolution in non-neoplastic IBD-affected colon" https://doi.org/10.1016/j.cell.2020.06.036 (https://doi.org/10.1016/j.cell.2020.06.036), we found that genes in the IL-17 and TLR pathways were enriched in somatic mutations in the colonic mucosa, even though no individual genes reached significance. We are interested in seeing if we can see evidence of positive selection on the pathway-level.

The mutation spectra for both UV-light and psoralen exposure extend beyond the trinucleotide model. This doesn't make too much of a difference on a gene level but it makes sense to implement a pentanucleotide model when considering genes in aggregate.

Please see the R-script pathway_dnds_pentanuc_model.r for details on how this was done. The script can be found in the Github repository accompanying this manuscript (see main text).

```
geneLists <- read.table("/lustre/scratch126/humgen/projects/psoriasis/selection_analyses/pathway_dNdS_geneLists.t
xt")

df <- data.frame()
for(geneL in geneLists$V1) {
  results <- read.table(paste("/lustre/scratch126/humgen/projects/psoriasis/selection_analyses/pathway_pentamodel
/", geneL, "_Full3075_1x2w_model_dNdSvals.txt", sep=""),h=T)
  results$pathway <- geneL
  df <- rbind(df, results)
}

df <- df[df$omega %in% c("wmis_driv", "wnon_driv"),]
df <- df[df$omega!="r_drivpass",]
df$q <- p.adjust(df$P, method="BH")
df
```

```
##        omega       MLEs      lowbd     highbd            P         pathway
## 1  wmis_driv  2.2122135  1.7260880  2.8352486 3.574584e-10 Normal_skin_pos
## 2  wnon_driv 15.1478793 11.4118277 20.1070551 6.369498e-79 Normal_skin_pos
## 8  wmis_driv  1.3521823  0.9481951  1.9282920 9.567306e-02             BCC
## 9  wnon_driv  2.0523151  1.0579729  3.9811958 3.344857e-02             BCC
## 15 wmis_driv  1.3442398  1.0276268  1.7584015 3.086267e-02  GWAS_psoriasis
## 16 wnon_driv  0.9190429  0.4436065  1.9040295 8.202964e-01  GWAS_psoriasis
## 22 wmis_driv  0.9146156  0.7195190  1.1626124 4.659323e-01            IL17
## 23 wnon_driv  0.7706908  0.4032517  1.4729367 4.306066e-01            IL17
## 29 wmis_driv  1.0720342  0.7882336  1.4580160 6.575299e-01         IL12_23
## 30 wnon_driv  1.2761316  0.6541418  2.4895396 4.745208e-01         IL12_23
## 36 wmis_driv  0.9657801  0.7262132  1.2843767 8.108145e-01             TNF
## 37 wnon_driv  0.9268954  0.4638567  1.8521561 8.298205e-01             TNF
## 43 wmis_driv  0.7897253  0.6364000  0.9799907 3.207389e-02      IL36_MyD88
## 44 wnon_driv  0.6972013  0.3866221  1.2572733 2.305544e-01      IL36_MyD88
## 50 wmis_driv  1.1340048  0.9251650  1.3899865 2.259126e-01            IFNg
## 51 wnon_driv  0.9961984  0.6026683  1.6466954 9.881488e-01            IFNg
## 57 wmis_driv  1.0128463  0.9191600  1.1160817 7.965931e-01      MHC_classI
## 58 wnon_driv  1.2348756  1.0040527  1.5187627 4.568710e-02      MHC_classI
## 64 wmis_driv  0.9012028  0.7783051  1.0435065 1.643321e-01             TLR
## 65 wnon_driv  0.6657081  0.4388242  1.0098971 5.566522e-02             TLR
## 71 wmis_driv  1.2560263  0.8493946  1.8573253 2.534041e-01      IBD_mucosa
## 72 wnon_driv  0.9814744  0.3492549  2.7581346 9.717045e-01      IBD_mucosa
##               q
## 1  3.932042e-09
## 2  1.401290e-77
## 8  2.631009e-01
## 9  1.471737e-01
## 15 1.471737e-01
## 16 9.128025e-01
## 22 6.959638e-01
## 23 6.959638e-01
## 29 9.041037e-01
## 30 6.959638e-01
## 36 9.128025e-01
## 37 9.128025e-01
## 43 1.471737e-01
## 44 4.611087e-01
## 50 4.611087e-01
## 51 9.881488e-01
## 57 9.128025e-01
## 58 1.675194e-01
## 64 4.017008e-01
## 65 1.749478e-01
## 71 4.645741e-01
## 72 9.881488e-01
```

Effects of Psoralen exposure

There is some literature (https://www.cell.com/cell-stem-cell/pdfExtended/S1934-5909(18)30402-8) out there to suggest that the selection of TP53 in particular is affected by UV-light exposure. We find no evidence that TP53 or any other gene is particulary selected for in the Psoralen-exposed skin. Please remember that the absence of evidence is not evidence of absence and we may simply lack power to detect differences in selection between psoralen-exposed and non-exposed skin.

```
cluster_burden <- read.table("/nfs/users/nfs_s/so11/phd/psoriasis/bsub_jupyter_lab/psoriasis/manuscript_data_and_
figures/Supplementary_material/Supplementary_Table3_clone_mutationBurden.txt", h=T)

## Mutations in samples with high PUVA exposure
puva_muts <- all_mutations[all_mutations$ClusterID %in% cluster_burden$CloneID[cluster_burden$PUVA>100],c("Microb
iopsyID", "Chr", "Pos_hg38", "Ref", "Alt")]

colnames(puva_muts) = c("SampleID", "Chr", "Pos", "Ref", "Alt")

puva_dnds = dndscv(puva_muts, refdb=refcds_38, cv=scores,max_muts_per_gene_per_sample = Inf, max_coding_muts_per_
sample = Inf)

sel_cv_puva <- puva_dnds$sel_cv
sel_cv_puva[sel_cv_puva$gene_name=="TP53",]
```

```
##       gene_name n_syn n_mis n_non n_spl  wmis_cv wnon_cv wspl_cv   pmis_cv
## 17180      TP53     0     2     0     0 10.46806       0       0 0.0182516
##       ptrunc_cv pallsubs_cv   qmis_cv qtrunc_cv qallsubs_cv
## 17180 0.8279757  0.06006747 0.8080215 0.9628665   0.9805273
```

```
sel_cv_indel[sel_cv_indel$gene_name=="TP53",]
```

```
##       gene_name n_syn n_mis n_non n_spl n_ind wmis_cv  wnon_cv  wspl_cv
## 17180      TP53     1    23     5     0     3 8.88856 19.57705 19.57705
##        wind_cv      pmis_cv    ptrunc_cv  pallsubs_cv      pind_cv      qmis_cv
## 17180 20.96863 2.600571e-10 1.531432e-05 5.375256e-12 0.0009622535 2.498628e-06
##        qtrunc_cv  qallsubs_cv pglobal_cv qglobal_cv      pdbs_cv  wdbs_cv n_dbs
## 17180 0.04035876 2.582273e-08          0          0 1.545748e-10 17.31495    13
```
