# Supplementary material for: Effects of psoriasis and psoralen exposure on the somatic mutation landscape of the skin
Source: Nat Genet. 2023 Oct 26;55(11):1892–900. doi: 10.1038/s41588-023-01545-1 (PMC10632143; doi:10.1038/s41588-023-01545-1)
Supplement: Supplementary file 2 — Reporting Summary [file 41588_2023_1545_MOESM2_ESM.pdf]

nature portfolio

Corresponding author(s): Carl A. Anderson

Last updated by author(s): Jul 17, 2023

Reporting Summary

Nature Portfolio wishes to improve the reproducibility of the work that we publish. This form provides structure for consistency and transparency in reporting. For further information on Nature Portfolio policies, see our Editorial Policies and the Editorial Policy Checklist.

Statistics

For all statistical analyses, confirm that the following items are present in the figure legend, table legend, main text, or Methods section.

n/a	Confirmed	
☐	☒	The exact sample size (*n*) for each experimental group/condition, given as a discrete number and unit of measurement
☐	☒	A statement on whether measurements were taken from distinct samples or whether the same sample was measured repeatedly
☐	☒	The statistical test(s) used AND whether they are one- or two-sided<br>*Only common tests should be described solely by name; describe more complex techniques in the Methods section.*
☐	☒	A description of all covariates tested
☐	☒	A description of any assumptions or corrections, such as tests of normality and adjustment for multiple comparisons
☐	☒	A full description of the statistical parameters including central tendency (e.g. means) or other basic estimates (e.g. regression coefficient) AND variation (e.g. standard deviation) or associated estimates of uncertainty (e.g. confidence intervals)
☐	☒	For null hypothesis testing, the test statistic (e.g. *F*, *t*, *r*) with confidence intervals, effect sizes, degrees of freedom and *P* value noted<br>*Give P values as exact values whenever suitable.*
☐	☒	For Bayesian analysis, information on the choice of priors and Markov chain Monte Carlo settings
☒	☐	For hierarchical and complex designs, identification of the appropriate level for tests and full reporting of outcomes
☐	☒	Estimates of effect sizes (e.g. Cohen's *d*, Pearson's *r*), indicating how they were calculated

Our web collection on statistics for biologists contains articles on many of the points above.

Software and code

Policy information about availability of computer code

Data collection

No software was used to collect the data.

Data analysis

Code supporting the main analyses of the manuscript is provided as R-markdown supplementary code files. Custom scripts documenting the mutation filtering and clustering pipelines, signature extraction and more are publicly available at https://github.com/Solafsson/somaticPsoriasis.
The following open source software tools were used as part of our analyses:

VerifyBamID (v1.1.3)
Picard (v.1.131)
samtools (v.1.11)
CaVEMan (v.1.15.1)
cgpPindel (v.3.5.0)
Bedtools (v. 2.18)
dNdScv (v. 0.0.1.0)

R-packages:
label.switching (v1.8)
hdp (v.0.1.5)
MutationalPatterns (v. 3.4.0)
BSgenome (v.1.60.0)
TxDb.Hsapiens.UCSC.hg38.knownGene (v.3.13.0)

TCGAmutations (v.0.3.0)

For manuscripts utilizing custom algorithms or software that are central to the research but not yet described in published literature, software must be made available to editors and reviewers. We strongly encourage code deposition in a community repository (e.g. GitHub). See the Nature Portfolio guidelines for submitting code & software for further information.

Data

Policy information about availability of data

All manuscripts must include a data availability statement. This statement should provide the following information, where applicable:

- Accession codes, unique identifiers, or web links for publicly available datasets
- A description of any restrictions on data availability
- For clinical datasets or third party data, please ensure that the statement adheres to our policy

Raw sequencing data are available in the European Genome-phenome Archive (EGA) using study ID EGAS00001004882 and dataset ID EGAD00001011265. Intermediary and supporting files, including mutation calls, mutational cluster assignments, phylogenetic trees, histological images, spatial relationship matrices and more are available in a Mendeley data repository (doi: 10.17632/phvh82vd9g.1). The mutation calls from the TCGA project can be obtained by using the TCGAmutations package in R (https://github.com/PoisonAlien/TCGAmutations). Access to the mutation calls of the HMF cohort can be obtained using the request forms found at https://www.hartwigmedicalfoundation.nl/en/.

Research involving human participants, their data, or biological material

Policy information about studies with human participants or human data. See also policy information about sex, gender (identity/presentation), and sexual orientation and race, ethnicity and racism.

Reporting on sex and gender	We report the sex of all participants in Supplementary Table 1. Data on gender was not collected. Participants were not stratified by sex in any of the analyses reported in the manuscript.
Reporting on race, ethnicity, or other socially relevant groupings	All participants are of white-European ancestry.
Population characteristics	All participants have been diagnosed with psoriasis vulgaris. The age, sex, disease duration and PUVA treatment history is provided in Supplementary table 1. The age of the participants varied from 18 to 88 and the disease duration varied from 0 to 67 years.
Recruitment	We recruited psoriasis patients presenting to the Department of Dermatology, UKSH Kiel. We note that the recruitment is biased in favor of men. The prevalence of psoriasis is thought to be the same in men and women but women only make up 19% of our cohort. It is possible that psoriasis affects men more severely than women, causing them to present to dermatologists more often and/or to be more eager to consent to participate in research into the disease.
Ethics oversight	The study was approved by the research ethics committee of Christian-Albrechts University in Kiel (A100/12), the National Health Service (NHS) Research Ethics Committee (Yorkshire & The Humber - South Yorkshire Research Ethics Committee, REC ID 20/YH/0244, IRAS ID 286843) and by the Wellcome Trust Sanger Institute Human Materials and Data Management Committee (approval number 20/0085).

Note that full information on the approval of the study protocol must also be provided in the manuscript.

Field-specific reporting

Please select the one below that is the best fit for your research. If you are not sure, read the appropriate sections before making your selection.

☒ Life sciences ☐ Behavioural & social sciences ☐ Ecological, evolutionary & environmental sciences

For a reference copy of the document with all sections, see nature.com/documents/nr-reporting-summary-flat.pdf

Life sciences study design

All studies must disclose on these points even when the disclosure is negative.

Sample size	This is an exploratory study and sample size was not pre-determined. We included 111 patients from whom we had samples available at the time of study initiation.
Data exclusions	We excluded from the whole study 6 microbiopsies suspected of being sample swaps and/or contaminated with external DNA. 101 microbiopsies were excluded only from the analysis of structural variants either due to the inability of the algorithm to find an optimal solution or if the goodness-of-fit for the optimal solution was <90%.
Replication	The results presented in this manuscript were not replicated in an independent cohort. We did carry out whole-exome sequencing of 18 near-duplicate samples that represent the same histological features on adjacent sections to quantify the sensitivity of our mutation calling pipeline. From this, we estimated a sensitivity of 89% of our mutation calling pipeline as described in the main text.

Randomization	This is a descriptive study with no interventions that recruited only patients and no controls. There was no need for randomization.
Blinding	This is a descriptive study with no interventions. The researchers were not blinded to the phenotypes of the participants.

Reporting for specific materials, systems and methods

We require information from authors about some types of materials, experimental systems and methods used in many studies. Here, indicate whether each material, system or method listed is relevant to your study. If you are not sure if a list item applies to your research, read the appropriate section before selecting a response.

Materials & experimental systems

n/a	Involved in the study
☒	☐ Antibodies
☒	☐ Eukaryotic cell lines
☒	☐ Palaeontology and archaeology
☒	☐ Animals and other organisms
☒	☐ Clinical data
☒	☐ Dual use research of concern
☒	☐ Plants

Methods

n/a	Involved in the study
☒	☐ ChIP-seq
☒	☐ Flow cytometry
☒	☐ MRI-based neuroimaging
